# Functional polysaccharide-based biosorbents as efficient green alternatives for radionuclide removal from radioactive waste: a review

**DOI:** 10.1039/d6ra02524b

**Published:** 2026-08-03

**Authors:** Mohamed Mohamady Ghobashy, Huda Yousif Sharef, Muneer Baata, Mohammed S. Almoiqli, Ahmed Siddiq, Muhamed Azlzul Haque, Faisal K. Algethami, Mohamed S. Attia

**Affiliations:** a Radiation Research of Polymer Department, National Center for Radiation Research and Technology (NCRRT), Atomic Energy Authority P. O. Box. 29, Nasr City Cairo Egypt Mohamed_ghobashy@yahoo.com Mohamed.ghobashy@eaea.org.eg; b Department of Chemistry, College of Education, Salahaddin University-Erbil Erbil Iraq; c College of Health Sciences, Catholic University in Erbil Kurdistan Region Iraq; d Desalination Technologies Institute (DTI), King Abdulaziz City for Science and Technology Riyadh 11442 Saudi Arabia; e Department of Chemistry, Faculty of Science, Al-Azhar University Assiut 71524 Egypt; f Biotechnology Department, University of Yeungnam Gyeongbouk 35841 Republic of Korea; g Chemistry Department, College of Science, Imam Mohammad Ibn Saud Islamic University (IMSIU) Riyadh 11623 Saudi Arabia mgaber@imamu.edu.sa

## Abstract

Radioactive waste presents persistent environmental and health challenges, creating an urgent need for remediation materials that are not only effective and selective but also sustainable. Conventional treatment methods often suffer from high cost, limited selectivity, and secondary waste generation, prompting growing interest in biopolymer-based alternatives. Polysaccharides including chitosan, alginate, cellulose, starch, carrageenan, and pectin have emerged as highly promising platforms for radioactive ion removal due to their natural abundance, biodegradability, and rich surface chemistry. Their functional groups (hydroxyl, amino, carboxyl, and sulfate) enable strong coordination with diverse radionuclides, including actinides, fission products, and activation products. This review critically examines the structure–property relationships governing radionuclide adsorption by polysaccharide-based materials, with particular attention to chemical modification strategies, including grafting, cross-linking, phosphorylation, and hybridization. These approaches significantly enhance adsorption capacity, selectivity, and stability, with reported Uranium(vi) uptake exceeding 600 mg g^−1^ for engineered systems. Advanced composites, including polysaccharide–polysaccharide hybrids and polysaccharide-based metal–organic frameworks, are highlighted for their potential in treating complex radioactive waste streams. Future perspectives focus on multifunctional material design, regeneration efficiency, and scalability to advance polysaccharide-based adsorbents toward practical nuclear waste management applications.

## Abstract 2

Natural polysaccharides such as chitosan, alginate, cellulose, starch, carrageenan, and pectin are sustainable adsorbents for radionuclide removal. Engineered modifications enhance selectivity and capacity, achieving U(vi) uptake above 600 mg g^−1^.

## Graphical abstract

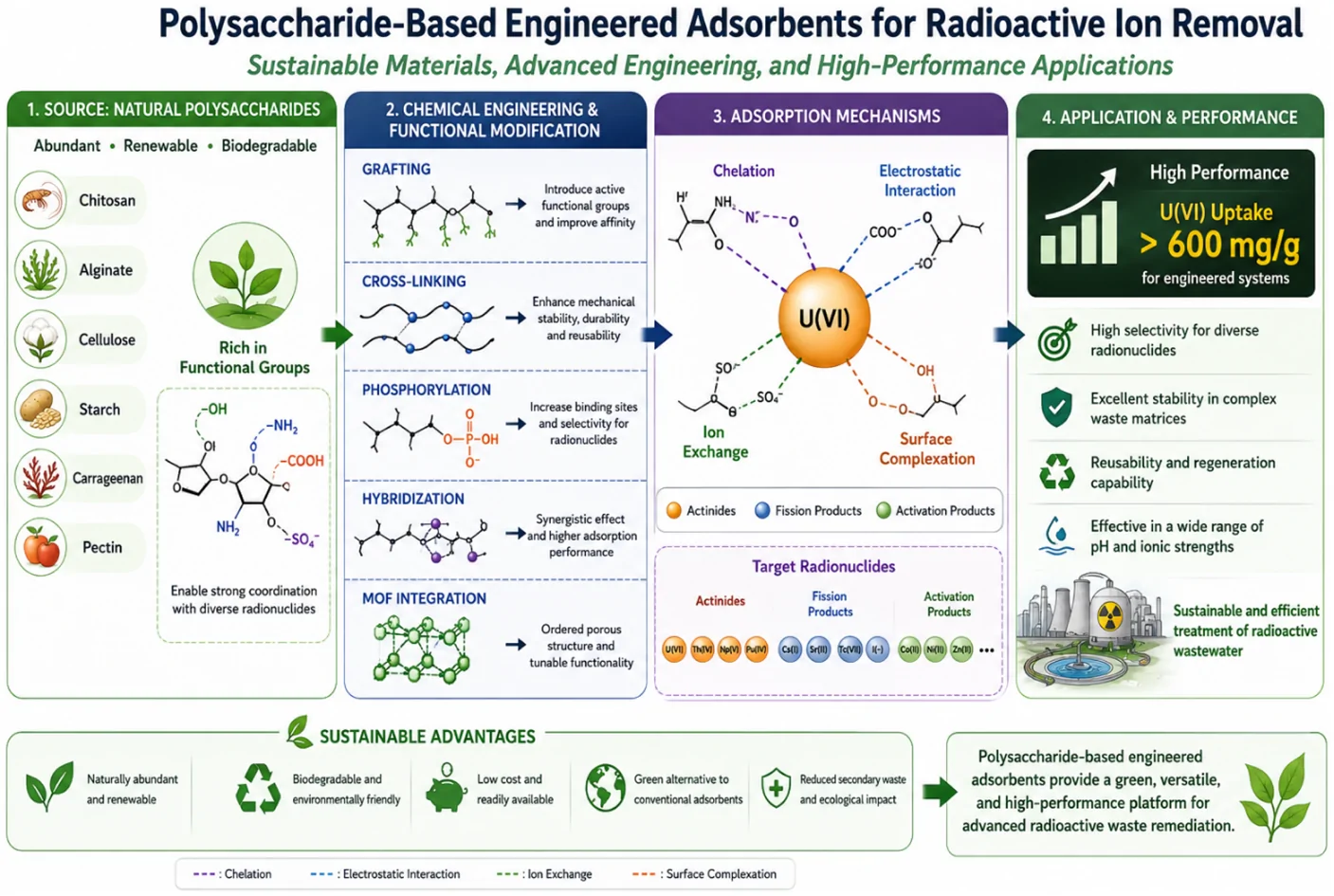

## 1. Introduction

The global expansion of nuclear energy, coupled with the growing use of medical isotopes and the ongoing reprocessing of nuclear fuel, has led to the continuous production of radioactive waste. This challenge extends far beyond the confines of nuclear facilities. The waste introduce radionuclides such as uranium, plutonium, cesium, strontium, technetium, iodine, and cobalt into the environment, many of which have long half-lives, high radiotoxicity, and the capacity to migrate through aquatic systems. Once released, these radioactive species can persist for decades to millennia, infiltrate food webs, and inflict significant biological harm even at trace concentrations. The long-lasting and pervasive nature of these contaminants highlights the urgent need for innovative, efficient, and sustainable waste management strategies.^1,2^ The environmental and health implications of radioactive waste make its management one of the most pressing challenges of our time. Globally, over 250 000 tons of high-level nuclear waste are currently in storage globally and, with continuous production from nuclear power plants, the need for effective treatment solutions has never been more urgent.^3^ Radioactive contaminants such as uranium (U), technetium (Tc), strontium (Sr), and cesium (Cs) pose significant threats to ecosystems and human health due to their long half-lives and radiotoxicity.^4^ For instance, Sr-90, with a half-life of 28 years, mimics calcium in biological systems and accumulates in bones, potentially causing bone cancer and leukemia.^5^ Similarly, Tc-99, a long-lived fission product (*t*_1/2_ = 2.13 × 10^5^ years), exists as the highly mobile pertechnetate anion (TcO_4_^−^) in aqueous systems, making its removal particularly challenging.^6^

Conventional treatment methods, such as chemical precipitation, ion exchange, and solvent extraction, have several limitations. While chemical precipitation generates large volumes of secondary waste, ion exchange resins often lack selectivity for specific radionuclides.^7^ Synthetic adsorbents like activated carbon and zeolites, though widely used, are non-renewable and can be costly to produce at scale.^8^ Moreover, these methods frequently require harsh chemical conditions, increasing operational costs and environmental impacts.^9^ These challenges have driven research toward the development of sustainable, selective, and cost-effective alternatives for radionuclide removal, with particular focus on bio-based materials that offer environmental compatibility and renewability.^10^

In recent years, natural polysaccharides have emerged as highly promising green adsorbents for radioactive waste treatment due to their biodegradability, renewability, and abundance of functional groups.^11^ Unlike synthetic polymers derived from petrochemical sources, polysaccharides such as chitosan, alginate, cellulose, and pectin are sourced from renewable natural materials, including crustacean shells, seaweed, plant biomass, and agricultural residues, making them both environmentally sustainable and cost-effective.^12^ Their molecular structures are rich in hydroxyl, amino, and carboxyl groups, which readily participate in coordination, electrostatic interactions, and ion-exchange processes with radionuclides.^13^ These functional groups are key to their affinity for both cationic and anionic species, positioning polysaccharides as attractive candidates for the selective recovery of radioactive elements from contaminated waters and complex waste matrices. Biopolymers such as chitosan, cellulose, and alginate offer clear sustainability advantages over conventional synthetic adsorbents, combining environmental compatibility with competitive performance.^14,15^ These materials are derived from abundant feedstocks crustacean shell waste for chitosan, lignocellulosic biomass for cellulose, and marine algae for alginate ensuring a low environmental footprint.^16^ However, native or unmodified polysaccharides often show limitations, including moderate adsorption capacities, poor resistance to chemical or radiolytic degradation, and reduced mechanical stability under harsh processing conditions.^17^ These drawbacks can restrict their direct applicability to nuclear waste treatment scenarios, which often involve acidic or alkaline environments, high ionic strength, and mixed contaminants.

To overcome these shortcomings, extensive research has focused on chemical and physical modification strategies to enhance the sorption properties of polysaccharides. Common approaches include cross-linking, graft copolymerization, and functional group grafting, which can improve adsorption performance by increasing surface area, introducing high-affinity binding sites, and enhancing structural stability.^18,19^ Chitosan, in particular, has been widely studied due to its abundance of amino and hydroxyl groups, which can be selectively modified to optimize binding to specific radionuclides.^15^

**Fig. 1:**
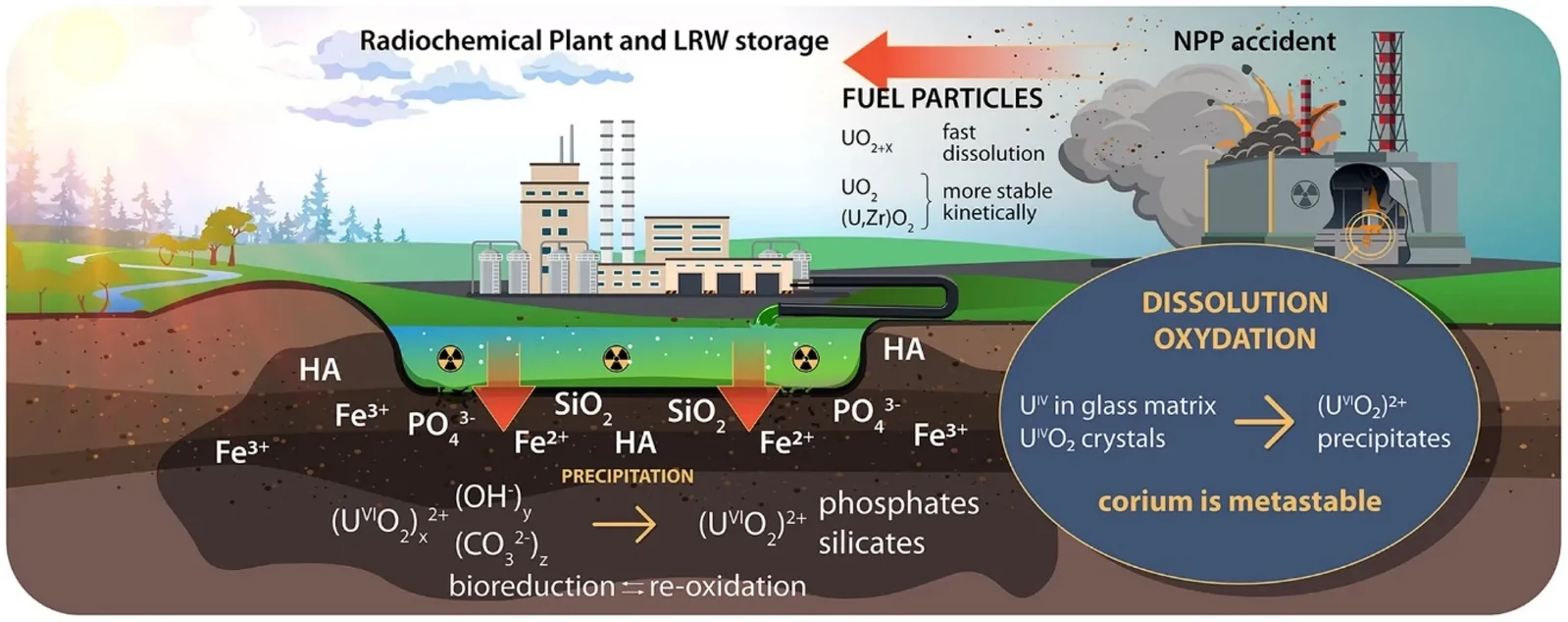
Schematic of uranium speciation, redox transformations, and mobility pathways in natural and contaminated environments. Shows U(iv), U(v), and U(vi) interconversion, complexation with carbonate, phosphate, and organic matter, and interactions with minerals and microorganisms. Includes anthropogenic sources, “hot” particles, and immobilization strategies such as phosphate precipitation and alkaline treatment, this figure has been adapted from ref. 20 with permission from Frontiers Media SA, copyright 2020.

Fig. 1 presents an integrated schematic illustrating the key forms, transformation pathways, and environmental factors influencing uranium speciation, mobility, and immobilization under both natural geochemical conditions and contamination scenarios. The diagram is organized to show uranium three relevant oxidation states in the environment U(iv), U(v), and U(vi) and the primary processes that control their interconversion. U(iv), typically occurring as crystalline UO_2_ or in mixed phases such as (U, Zr)O_2_ from nuclear fuel particles, is positioned in the reducing, low-oxygen sector of the figure. It is depicted as a relatively immobile form, stable in anoxic environments such as deep geological disposals or within “hot” particles from nuclear accidents. Arrows indicate that U(iv) can be oxidized to U(vi) under oxygen-rich conditions or with specific oxidants, thereby increasing mobility.

In contrast, U(vi) is shown at the center of the aqueous mobility domain, mainly occurring as the uranyl cation (UO_2_^2+^) in solution. This highly mobile species forms complexes with hydroxide, carbonate, phosphate, and sulfate ions, as well as with dissolved silica and organic matter. The figure highlights the ternary Ca–U(vi)–CO_3_ system, emphasizing the formation of calcium–uranyl–carbonate complexes [*e.g.*, Ca_2_UO_2_(CO_3_)_3_(aq)], which reduce sorption onto mineral surfaces and enhance transport in groundwater. Lines branching from the U(vi) domain connect to sorption, precipitation, and incorporation processes involving calcite, clays, iron oxides, and other environmental minerals. The presence of Fe(ii)/Fe(iii) oxides and oxyhydroxides is shown as a critical pathway for partial reduction of U(vi) to U(v), with arrows indicating that U(v) may either disproportionate back to U(iv) and U(vi) or be stabilized within mineral matrices.

Given the urgent need for environmentally sustainable nuclear waste management, this review critically examines the role of polysaccharide-based adsorbents in radionuclide removal. The scope encompasses (i) the structural features and intrinsic properties of key polysaccharides that underpin their adsorption behavior, (ii) chemical modification strategies including crosslinking, grafting, and ligand functionalization used to enhance performance, (iii) hybrid materials that integrate polysaccharides with other functional phases for improved selectivity and stability, and (iv) mechanistic insights into the adsorption processes.^21^

Special attention is devoted to studies that connect structural modifications with adsorption outcomes, including the use of spectroscopic and computational methods to unravel binding mechanisms at the molecular level. Additionally, this review identifies current challenges, including stability under radiation, performance in complex ionic environments, and regeneration efficiency. It outlines future directions, including the design of multifunctional composites, integration with membrane technologies, and life-cycle assessments to evaluate environmental and economic impacts.

By systematically comparing different polysaccharide platforms and functionalization approaches, this work aims to provide a roadmap for developing next-generation green adsorbents capable of addressing the dual challenges of effective radionuclide recovery and sustainable nuclear waste management.^21^

The review also critically assesses real-world applications of polysaccharide adsorbents, discussing their performance in simulated and actual radioactive waste streams. Challenges such as competitive ion effects, pH dependence, and material regeneration are examined, along with potential solutions. Recent advances in hybrid materials (*e.g.*, polysaccharide-MOF composites) and process intensification (*e.g.*, membrane filtration systems) are highlighted as promising directions for future research.

Finally, the review identifies key knowledge gaps and proposes future research directions. These include standardized testing protocols, life-cycle assessments of polysaccharide adsorbents, and scale-up studies for industrial implementation. By providing this comprehensive perspective, the review aims to accelerate the development of effective, sustainable, and economically viable polysaccharide-based technologies for nuclear waste remediation.

## 2. Target radioactive species

### 2.1. Actinides: uranium (U^3+^, UO_2_^2+^), plutonium (Pu^4+^), neptunium (Np^4+^)

Wang*et al.*^22^ conducted comprehensive studies on uranium speciation in aqueous solutions, demonstrating that uranium exists primarily in two oxidation states under environmental conditions: the trivalent U^3+^ form and the more prevalent uranyl ion UO_2_^2+^. The uranyl ion represents the most thermodynamically stable form of uranium in oxidizing aqueous environments. It poses the most significant environmental concern due to its high solubility and mobility in groundwater systems. Their research revealed that UO_2_^2+^ exhibits strong complexation with various ligands commonly found in nuclear waste streams, including carbonate, phosphate, and organic acids, thereby significantly complicating separation processes by reducing the availability of free uranyl ions for direct adsorption.

Müller *et al.*^23^‏, Maher *et al.*,^24^ and Romanchuk *et al.*^20^ investigated the hydrolysis behavior of actinides in nuclear waste solutions, focusing on the pH-dependent speciation of uranium, plutonium, and neptunium. Their findings indicated that uranium hydrolysis begins at pH 3.5, leading to the formation of polynuclear species such as (UO_2_)_2_(OH)_2_^2+^ and (UO_2_)_3_(OH)_5_^+^ at intermediate pH values, while complete precipitation as uranyl hydroxide occurs at pH values exceeding 6.5. This complex speciation behavior directly impacts the effectiveness of various separation technologies, as different uranium species exhibit dramatically different adsorption affinities and kinetics. The study also highlighted that plutonium exists in multiple oxidation states simultaneously (Pu^3+^, Pu^4+^, PuO_2_^+^, and PuO_2_^2+^) even under controlled conditions, making selective separation extremely challenging.

Shcherbina *et al.*^25^ ‏examined the environmental behavior and transport mechanisms of neptunium in subsurface environments, demonstrating that Np^4+^ represents the most stable oxidation state under reducing conditions commonly found in deep geological repositories. Their research showed that neptunium exhibits unique sorption behavior compared to other actinides, with significantly lower distribution coefficients on common mineral surfaces, resulting in enhanced mobility and increased potential for groundwater contamination. The study revealed that Np^4+^ forms strong complexes with humic substances and other natural organic matter, which can either enhance or inhibit its removal, depending on the specific treatment approach.

Knopp *et al.*^26^‏ conducted systematic studies on plutonium chemistry in high-level waste solutions, revealing that Pu^4+^ demonstrates the strongest tendency toward hydrolysis and polymerization among all actinides. Their investigation showed that Pu^4+^ readily forms colloidal species and polynuclear complexes even at relatively low concentrations and acidic pH conditions, leading to unpredictable behavior during separation processes. The research highlighted that plutonium's ability to exist in four oxidation states simultaneously creates significant analytical and separation challenges, as each state exhibits distinct chemical behavior and requires distinct treatment approaches for effective removal.

### 2.2. Fission products: cesium (^137^Cs^+^), strontium (^90^Sr^2+^), iodine (^129^I^−^)

Nuclear fission generates a wide array of radioactive isotopes, among which cesium-137 (^137^Cs^+^), strontium-90 (^90^Sr^2+^), and iodine-129 (^129^I^−^) are of particular concern due to their environmental persistence, biological uptake, and radiotoxicity.^27^ These radionuclides are released during nuclear reactor accidents, weapons testing, and fuel reprocessing, leading to long-term ecological and public health challenges that continue to shape our understanding of radiological risk assessment and environmental monitoring.

Cesium-137 is a beta- and gamma-emitter with a half-life of 30.17 years, making it one of the most hazardous fission products encountered in environmental contamination scenarios.^27^ Due to its chemical similarity to potassium, cesium-137 is readily absorbed by plants through their root systems and subsequently bioaccumulates throughout the food chain, posing significant risks to both human and ecological health. The Chernobyl disaster in 1986 released approximately 85 PBq of ^137^Cs^+^ into the atmosphere, contaminating vast areas across Europe and creating a legacy of environmental contamination that persists to this day.^28^

In the aftermath of Chornobyl, agricultural lands in Belarus, Russia, and Ukraine remained restricted for decades due to elevated levels of cesium-137 in crops, livestock, and dairy products.^29^ The contamination was particularly severe in areas with acidic soils and forest ecosystems, where cesium-137 remained bioavailable for extended periods. Similarly, the Fukushima Daiichi accident in 2011 dispersed cesium-137 across eastern Japan, with significant contamination found in rice paddies, tea plantations, and marine environments.^30^ Long-term monitoring studies have revealed that wild animals, particularly wild boar in Fukushima Prefecture, continue to exhibit elevated cesium-137 levels due to persistent soil contamination and their foraging behavior in contaminated forest areas.^31^

Strontium-90 is a pure beta emitter with a half-life of 28.8 years that behaves similarly to calcium in biological systems, leading to preferential accumulation in bones and teeth upon ingestion.^32^ Its radiological impact was first recognized during the era of atmospheric nuclear weapons testing in the 1950s and 1960s, when global fallout led to widespread strontium-90 deposition across the planet. The famous “Baby Tooth Survey” conducted in the United States during this period demonstrated that children born during peak nuclear testing periods had significantly higher strontium-90 levels in their teeth, with epidemiological studies later correlating these exposures with increased risks of leukemia and bone cancer.^33^

The Chornobyl accident released approximately 10 PBq of strontium-90, creating localized hotspots of contamination, particularly near the reactor site and in areas where rainfall occurred during the initial plume passage.^34^ Long-term studies in Ukraine have shown that strontium-90 remains a persistent contaminant in the food chain, with elevated levels continuing to be detected in milk and bone tissues of local populations decades after the accident.^35^ Additionally, nuclear weapons production facilities such as the Mayak Production Association in Russia have contributed to environmental strontium-90 contamination through historical waste disposal practices, with radioactive materials leaking into nearby rivers and affecting downstream ecosystems for hundreds of kilometers.^36^

Iodine-129 poses a unique challenge among fission products due to its extraordinarily long half-life of 15.7 million years, making it a permanent environmental contaminant once released.^37^ While it is less immediately hazardous than its short-lived counterpart iodine-131 (half-life of 8 days), iodine-129 poses significant long-term risks due to its mobility in aquatic environments and its potential for thyroid uptake in both humans and wildlife. Unlike other fission products that may decay to negligible levels within decades or centuries, iodine-129 will remain radiologically significant for geological timescales.

The Sellafield nuclear fuel reprocessing plant in the United Kingdom has historically been one of the largest point sources of iodine-129 contamination, discharging substantial quantities into the Irish Sea through liquid effluent releases.^38^ These discharges have led to measurable concentrations of iodine-129 in marine organisms, particularly seaweed and bottom-dwelling fish species that bioconcentrate iodine from seawater. Similarly, the Hanford Site in Washington State has reported elevated levels of iodine-129 in groundwater systems due to Cold War-era plutonium production activities, with contamination plumes extending several kilometers from the source areas.^39^ Unlike iodine-131, which decays rapidly after nuclear accidents and becomes undetectable within months, iodine-129 serves as a permanent signature of nuclear activities. It has become an important tracer for studying the long-term fate and transport of radioactive contamination in environmental systems.^40^

The long-term presence of these three fission products in the environment has necessitated the development of comprehensive monitoring programs and remediation strategies worldwide. Cesium-137 contamination has required extensive agricultural countermeasures, including applying potassium fertilizers to reduce plant uptake, restricting certain food products, and, in some cases, permanently abandoning agricultural land. Strontium-90 monitoring primarily focuses on dairy products and bone-seeking organisms, as this radionuclide poses the greatest risk *via* the calcium metabolic pathway. Iodine-129 surveillance has become increasingly important in the vicinity of nuclear facilities and in marine environments, where its extreme longevity makes it a permanent marker of anthropogenic nuclear activities.

The health implications of exposure to these fission products vary significantly depending on their biological behavior and radiological properties. Cesium-137 exposure primarily increases cancer risk through whole-body irradiation, as it distributes relatively uniformly throughout soft tissues. Strontium-90 presents a more focused risk profile, with bone and blood-forming organs being the primary targets for radiation-induced malignancies. Iodine-129, while having a lower specific activity than shorter-lived iodine isotopes, contributes to thyroid cancer risk over extended periods, particularly in populations with iodine-deficient diets where radioactive iodine uptake may be enhanced.

Cesium-137, strontium-90, and iodine-129 continue to serve as critical indicators for environmental monitoring and nuclear safety assessments decades after their initial release from major nuclear incidents. The lessons learned from historical events such as Chernobyl, Fukushima, and atmospheric weapons testing have fundamentally shaped our understanding of long-term radiological risk and have informed the development of improved reactor safety systems, waste management practices, and emergency response protocols. Effective remediation strategies, including soil removal and replacement, phytoremediation with hyperaccumulator plants, and food monitoring programs, remain essential components of managing contaminated environments.

Future research priorities must focus on improving long-term waste containment technologies, developing more effective decontamination methods, and advancing our understanding of the biogeochemical behavior of these radionuclides in diverse ecosystems. As the nuclear industry continues to evolve with new reactor designs and waste management approaches, the historical legacy of these three fission products serves as a constant reminder of the importance of rigorous safety protocols and environmental stewardship in nuclear technology applications. The extremely long environmental persistence of these radionuclides, particularly iodine-129, underscores the intergenerational responsibility that accompanies nuclear technology and the critical importance of preventing their release into the environment.

### 2.3. Activation products: cobalt (^60^Co^2+^), nickel (^63^Ni^2+^)

Activation products such as cobalt-60 (^60^Co) and nickel-63 (^63^Ni) are generated when stable isotopes of cobalt (^59^Co) and nickel (^62^Ni) are exposed to neutron radiation in nuclear reactors, particle accelerators, or during nuclear weapons testing. These radionuclides are of particular concern in nuclear waste management, reactor decommissioning, and occupational radiation safety due to their gamma and beta emissions, respectively.

Cobalt-60 (^60^Co) is a gamma-emitting radionuclide with a half-life of 5.27 years, produced primarily through neutron activation of stable cobalt-59 in nuclear reactor components such as steel alloys, control rods, and coolant systems.^41^ Due to its high-energy gamma emissions (1.17 and 1.33 MeV), ^60^Co is widely used in medical radiotherapy, industrial radiography, and food irradiation.^42^ However, it also poses significant radiological hazards when released into the environment.

One of the most notable cases of ^60^Co contamination occurred in Taiwan in 1982, where recycled steel contaminated with ^60^Co was inadvertently used in construction, leading to chronic radiation exposure in residential buildings.^43^ Similarly, at the Fukushima Daiichi Nuclear Power Plant, ^60^Co was detected in reactor cooling water and sediments, contributing to the overall radioactive inventory.^44^ Studies on nuclear decommissioning have highlighted ^60^Co as a major contributor to radiation fields in retired reactors, requiring careful shielding and disposal.^45^

Nickel-63 (^63^Ni) is a beta-emitting radionuclide with a half-life of 100.1 years, formed *via* neutron activation of nickel-62 in reactor structural materials.^46^ Unlike ^60^Co, ^63^Ni emits low-energy beta particles (67 keV), making it less hazardous externally but a concern when ingested or inhaled due to its long half-life and potential for internal deposition.^47^

Nuclear reprocessing plants, such as Sellafield in the UK and La Hague in France, have reported the presence of ^63^Ni in liquid effluents, which accumulate in marine sediments and shellfish.^48^ Additionally, ^63^Ni has been identified in radioactive waste from dismantled nuclear submarines, posing challenges for long-term storage.^49^ Recent research has also explored the use of ^63^Ni in betavoltaic batteries for medical and space applications due to its long half-life and stable decay properties.^50^

Cobalt-60 and nickel-63 are significant activation products with distinct radiological properties and environmental behaviors. While ^60^Co is a major concern in decommissioning and accidental exposures, ^63^Ni presents long-term challenges due to its persistence in waste streams and potential for bioaccumulation. Effective management strategies, including advanced waste segregation and containment, are essential to mitigate their impact.^45^

## 3. Chemical properties and separation challenges

The separation and environmental management of radionuclides present complex challenges due to their diverse chemical properties. The effective remediation of radionuclides such as cesium (Cs^+^), strontium (Sr^2+^), cobalt (Co^2+^), nickel (Ni^2+^), and actinides from contaminated environments depends heavily on their ionic characteristics, including ionic radii, charge density, hydration behavior, and speciation under varying pH and redox conditions.^51^ These properties influence their mobility in aqueous systems, their interactions with adsorbents, and their competition with non-radioactive ions, presenting significant challenges in nuclear waste treatment and environmental decontamination that extend beyond the commonly studied fission products to include less-studied radioactive elements.

### 3.1. Ionic radii, charge density, and hydration behavior

The ionic radii and charge density of radionuclides play a crucial role in their chemical behavior, particularly in ion-exchange and adsorption processes. Cesium (Cs^+^) has a large ionic radius (1.67 Å) and low charge density due to its single positive charge, making it weakly hydrated compared to smaller ions like sodium (Na^+^) or potassium (K^+^).^51^ This weak hydration allows Cs^+^ to preferentially bind to selective adsorbents such as Prussian blue analogs (PBAs) and clay minerals like vermiculite, where it can displace similarly sized K^+^ ions.^52^ This property explains cesium's environmental mobility, as demonstrated after the Fukushima accident where Cs^+^ migrated rapidly through soil profiles.^53^

In contrast, strontium (Sr^2+^) has a smaller ionic radius (1.18 Å) but a higher charge density, leading to stronger hydration and greater competition with divalent ions such as calcium (Ca^2+^) and magnesium (Mg^2+^).^54^ This makes Sr^2+^ removal challenging in groundwater and seawater, where high concentrations of competing ions reduce the efficiency of conventional ion-exchange resins.^55^ The strong hydration shell surrounding Sr^2+^ creates additional barriers to selective adsorption, as the energy required to partially dehydrate the ion for binding to adsorbent sites competes with the binding affinity itself.

Cobalt (Co^2+^) and nickel (Ni^2+^) exhibit intermediate ionic radii (0.74 Å and 0.69 Å, respectively) and high charge densities, resulting in strong hydration shells that influence their complexation with organic ligands and adsorption onto oxide surfaces.^56^ In nuclear reactor coolants, Co^2+^ often forms soluble complexes with corrosion products, complicating its removal and contributing to the activation of reactor components.^57^ The transition-metal nature of these ions also enables them to form stable coordination complexes with a variety of ligands, which can either enhance or hinder their separation, depending on the system design.

The trivalent actinides, such as americium (Am^3+^) and curium (Cm^3+^), pose even greater challenges, with their smaller ionic radii (europium as an analog: 0.947 Å) and high charge densities resulting in hydration energies exceeding 3000 kJ mol^−1^, necessitating strong complexing agents for separation.^42^ The hydration behavior of these ions further dictates their mobility in aqueous systems. Cesium, due to its weak hydration, migrates more readily through porous media and geological formations, making it a particular concern for groundwater contamination.^58^ Conversely, Sr^2+^ and transition metals like Co^2+^ and Ni^2+^ exhibit slower diffusion due to stronger water coordination, which can be advantageous for containment but challenging for extraction from solid matrices.

### 3.2. Interference from competing non-radioactive ions

A major challenge in radionuclide separation is the interference from non-radioactive ions, which compete for binding sites on adsorbents and ion-exchange resins. In natural waters, Cs^+^ competes with K^+^ and Na^+^, which are often present at much higher concentrations, with K^+^ reaching up to 400 mg L^−1^ in seawater environments.^59^ In seawater remediation specifically, Cs^+^ recovery competes with 0.01 M potassium concentrations, reducing the efficiency of conventional ion exchangers by up to 60%. Traditional cation exchangers, such as zeolites, exhibit poor Cs^+^ selectivity in such environments due to their similar ionic sizes and charges, necessitating the development of advanced materials, such as crystalline silicotitanates (CSTs), that provide enhanced selectivity through specific framework structures.^60^

For strontium separation, the primary competitors are Ca^2+^ and Mg^2+^, which dominate in hard water and seawater systems. In groundwater, Sr^2+^ separation is particularly complicated by 1–2 mM calcium concentrations, requiring highly selective materials like sulfonated covalent organic frameworks (COFs).^61^ Conventional resins like Dowex 50W-X8 exhibit low Sr^2+^ selectivity in high-Ca^2+^ matrices, prompting the development and use of macrocyclic ligands, such as crown ethers, that provide size-selective binding cavities for enhanced separation efficiency.^62^

Cobalt and nickel face competition from iron ions (Fe^2+^/Fe^3+^) and manganese (Mn^2+^) in corrosion-laden nuclear waste streams, where these competing ions are often present at concentrations orders of magnitude higher than the target radionuclides.^63^ For trivalent actinides, the presence of Fe^3+^ and Al^3+^ in nuclear waste streams poses particular challenges. Studies at the Hanford site demonstrated that Am^3+^ recovery decreased from 95% to 40% when Fe^3+^ concentrations exceeded 10 mM, driving the development of novel ligands, such as HDEHP (bis(2-ethylhexyl) phosphoric acid), that show preferential binding to actinides.^64^ Chelating resins containing iminodiacetate (IDA) or thiourea functional groups are often employed for their enhanced selectivity toward transition metals, but their efficiency decreases significantly at high Fe^3+^ concentrations due to iron's strong complexation tendency.

### 3.3. pH sensitivity and redox-dependent speciation

The speciation and solubility of radionuclides are highly pH-dependent, affecting their removal efficiency and requiring careful optimization of treatment conditions. Radionuclide behavior shows dramatic pH dependence across different chemical conditions. Under alkaline conditions (pH > 10), cesium (Cs^+^) remains predominantly as a free hydrated ion and is soluble across a wide pH range (2–12), making its adsorption less pH-sensitive than other radionuclides.^65^ However, at extremely high pH (>12), Cs^+^ may adsorb onto negatively charged clay edges *via* electrostatic interactions, though this mechanism is generally less significant than ion-exchange processes.^66^ In contrast, Sr^2+^ forms Sr(OH)_2_ precipitates under these same alkaline conditions.^67^

Strontium (Sr^2+^) exhibits minimal hydrolysis below pH 10, maintaining its divalent cationic form and allowing consistent ion-exchange behavior across neutral to mildly alkaline conditions.^67^ Above pH 11, however, Sr(OH)^+^ and Sr(OH)_2_ precipitation become dominant processes, reducing the ion's mobility but also limiting the effectiveness of adsorption-based treatment methods.

Cobalt and nickel exhibit more complex pH-dependent behavior due to their transition-metal nature. Cobalt (Co^2+^) undergoes hydrolysis above pH 7, forming Co(OH)^+^ species and eventually Co(OH)_2_ precipitates. In contrast, in oxidizing conditions, Co^3+^ oxides may form, significantly altering the chemical behavior and treatment requirements.^68^ Nickel (Ni^2+^) exhibits a similar hydrolysis pattern, with Ni(OH)_2_ precipitation above pH 8.5, though it remains soluble in acidic or highly complexing environments that contain organic ligands.^69^

The behavior of actinides and other radionuclides is further complicated. Under alkaline conditions, uranium(vi) transforms from mobile UO_2_^2+^ to immobile schoepite (UO_3_·2H_2_O). At the same time, technetium(vii) as TcO_4_^−^ remains soluble and plutonium(iv) forms Pu(OH)_4_ colloids.^70,71^ Under acidic conditions (pH < 4), most metal ions exhibit enhanced mobility, with the notable exception of Pu(iv), which polymerizes into colloids.^72^

Redox conditions further complicate speciation and treatment strategies. At the Savannah River Site, fluctuating redox conditions caused cycling between mobile Tc(vii) and immobile Tc(iv) forms, significantly impacting remediation strategies and demonstrating the need for comprehensive understanding of both pH and redox effects.^73^ These speciation changes create complex optimization challenges for treatment strategies, where acidic conditions (pH < 3) favor Co^2+^ and Ni^2+^ solubility but hinder adsorption onto most adsorbents due to protonation of surface sites, while neutral to alkaline conditions (pH 7–10) enhance Sr^2+^ and Cs^+^ uptake on clay minerals and other adsorbents but trigger Co and Ni hydroxide precipitation.

### 3.4. Emerging separation technologies and treatment strategy implications

The diverse chemical behaviors of these radionuclides necessitate tailored approaches for effective separation and remediation. For cesium removal, Prussian blue analogs and crystalline silicotitanates have emerged as promising materials due to their high selectivity even in the presence of competing alkali metals. Strontium separation often requires crown ether-based resins or specialized inorganic ion exchangers that can discriminate between Sr^2+^ and the abundant Ca^2+^ and Mg^2+^ ions in natural waters.

Recent advances address these challenges through innovative approaches. Hybrid materials, such as graphene oxide-functionalized crown ethers, achieve 90% Sr^2+^ removal even in seawater environments, demonstrating significant improvements over conventional technologies.^74^ Bioremediation approaches have also shown promise, with Geobacter species capable of reducing mobile U(vi) to insoluble U(iv) under mildly acidic to neutral conditions (pH 5–8), providing environmentally friendly treatment options.^75^ Ion-imprinted polymers represent another advancement, offering selectivity for Cs^+^ with 100-fold preference over Na^+^, addressing one of the major challenges in cesium separation.^76^

Transition-metal radionuclides such as Co^2+^ and Ni^2+^ benefit from chelating resins with soft donor atoms that preferentially coordinate to transition metals over alkali and alkaline-earth competitors. However, the strong complexation tendencies of these ions also make them susceptible to interference from organic matter and other complexing agents commonly found in nuclear waste streams. pH optimization represents a critical balance in multi-component systems, where conditions favoring one radionuclide's removal may be detrimental to another's capture efficiency. Sequential treatment processes or multi-stage systems often provide better overall performance than single-step approaches, allowing for optimization of conditions for each target species.

Their fundamental ionic properties govern the separation of radionuclides from complex matrices, the presence of competing ions, and pH-dependent speciation behavior. Effective remediation requires the development of tailored adsorbents such as Prussian blue analogs for Cs^+^ and crown ethers for Sr^2+^, careful pH optimization to balance solubility and adsorption effects, and advanced materials like crystalline silicotitanates and iminodiacetate resins to overcome ion competition challenges.

Effective radionuclide separation requires a comprehensive understanding of ionic properties governing hydration and selectivity, competitive ion effects in complex matrices, and pH- and redox-dependent speciation. Future research should focus on developing multifunctional materials capable of simultaneously capturing multiple radionuclides while maintaining performance under variable geochemical conditions. Priority areas include *in situ* characterization techniques for speciation analysis, advanced materials combining multiple selectivity mechanisms, and predictive modeling incorporating geochemical parameters.^42^

The integration of fundamental understanding of ion hydration, complexation, and surface interactions with practical engineering considerations will be essential for advancing radionuclide separation technologies to meet the growing challenges of nuclear waste management and environmental remediation. As nuclear technology continues to evolve, the development of robust, selective, and cost-effective separation methods remains critical for ensuring environmental protection and public safety.

## 4. Chitosan and chitin-based systems for radioactive ion recovery

Recent developments in radionuclide adsorption materials demonstrate a clear transition from conventional inorganic sorbents toward multifunctional polymeric and bio-derived hybrid systems designed to simultaneously improve adsorption capacity, selectivity, structural stability, and regeneration capability.^77^ Earlier studies largely focused on single-component materials such as clays,^78^ zeolites,^79^ ferrocyanides,^80^ and ion-exchange resins;^81^ however, these systems commonly suffered from limited selectivity, slow adsorption kinetics, poor accessibility of active sites, and difficult regeneration under complex wastewater conditions. Consequently, current research increasingly emphasizes chemically engineered composites that integrate functional adsorption groups with mechanically stable support matrices.

Among uranium-selective adsorbents, chitosan-based materials remain one of the most extensively investigated platforms due to the abundance of amino and hydroxyl functional groups that can coordinate strongly with uranyl ions.^82^ Nevertheless, pristine chitosan exhibits poor mechanical stability and limited resistance in acidic media, which significantly restricts its practical applicability in nuclear wastewater treatment. To overcome these drawbacks, several studies introduced additional functionalization strategies. For example, amidoxime-modified chitosan/bentonite composites significantly enhanced uranium affinity through the incorporation of amidoxime ligands, which are well known for their strong chelation with U(vi).^83^ The adsorption process followed pseudo-second-order kinetics and Langmuir monolayer behavior, indicating dominant chemisorption mechanisms. Moreover, bentonite incorporation improved structural stability and provided additional adsorption sites, although the maximum adsorption capacity remained moderate compared with more advanced hybrid systems.^84^

Similarly, phosphorylated chitosan-functionalized biochar materials introduced phosphate-rich functional groups capable of enhancing U(vi) complexation through strong coordination interactions.^85^ In addition to surface complexation, partial reduction of U(vi) to U(iv) was reported, suggesting a dual adsorption mechanism that contributed to the exceptionally high removal efficiency.^86^ However, while phosphate-rich systems improve affinity and selectivity, the stronger binding interactions may complicate regeneration and long-term reuse.^87^ Furthermore, biochar-supported systems often exhibit heterogeneous adsorption surfaces, leading to multilayer adsorption behavior rather than predictable monolayer uptake.

To address limitations associated with structural collapse and acid instability, hydrogel and aerogel architectures have recently gained considerable attention. Polyvinyl alcohol/chitosan/phytic acid aerogels represent an important advancement in this area, as the incorporation of polyvinyl alcohol significantly enhances mechanical integrity and acid resistance while maintaining high uranium selectivity. The interconnected porous structure facilitates rapid mass transfer and provides abundant accessible active sites, allowing efficient uranium separation even in real acidic wastewater containing competing ions. Nevertheless, although these aerogel systems demonstrate excellent stability and reusability, many studies primarily report removal efficiencies rather than standardized adsorption capacities, complicating direct comparison with other materials.

Beyond uranium, titanate nanotube-based composites have emerged as highly promising adsorbents for Cs^+^ and Sr^2+^ removal due to their layered ion-exchange structure and rapid adsorption kinetics.^88^ The embedding of titanate nanotubes into alginate matrices improves handling, recovery, and mechanical stability while reducing nanoparticle aggregation. However, this immobilization strategy also introduces diffusion limitations and partially blocks active adsorption sites, resulting in lower adsorption efficiency compared with free titanate nanotubes. Similar behavior has been observed in alginate-based nanobentonite composites for Co^2+^ and Eu^3+^ adsorption, where polymer encapsulation improves recyclability and operational practicality at the expense of adsorption capacity.^89^

Collectively, these studies reveal several important trends in the design of radionuclide adsorbents. First, multifunctional hybridization strategies combining inorganic nanostructures with biopolymer matrices consistently outperform single-component materials by improving both structural stability and adsorption selectivity. Second, the nature and density of functional groups, particularly amidoxime, phosphate, hydroxyl, and amino groups, strongly govern radionuclide affinity and adsorption mechanisms. Third, hierarchical porous architectures such as aerogels, hydrogels, and nanotube networks significantly enhance mass transfer and adsorption kinetics. Despite these advances, major challenges remain, including the lack of standardized benchmarking protocols, limited testing under realistic nuclear wastewater conditions, insufficient radiation-stability studies, and uncertainties regarding large-scale economic feasibility. Therefore, future efforts should focus not only on maximizing adsorption capacity but also on improving long-term durability, selectivity under competitive ionic environments, and scalable fabrication approaches suitable for practical deployment.

A comparative analysis of chitosan-based adsorbents reveals a clear hierarchy of effectiveness depending on the target radionuclide. For cesium (Cs^+^), capacities range widely from 22 mg g^−1^ to 490 mg g^−1^, with Prussian blue (PB) or copper hexacyanoferrate (CuHCF) incorporation consistently yielding the highest values Wang *et al.*^90^. Notably, bentonite alone achieves only modest Cs^+^ uptake (∼57 mg g^−1^), suggesting that the specific lattice structure of PB analogues not merely the presence of negatively charged sites is critical for selective Cs^+^ entrapment *via* ion exchange. In contrast, for uranium (U(vi)) and thorium (Th(iv)), phosphorylation or amine grafting (*e.g.*, PEI, TEPA) produces the highest reported capacities (380–667 mg g^−1^), far exceeding those achieved by carboxylate-rich modifications. This trend is mechanistically consistent: uranyl (UO_2_^2+^) forms strong inner-sphere complexes with phosphate and primary amine groups, whereas carboxylates (p*K*_a_ ∼4 and 5) become protonated and ineffective below pH 4. Indeed, optimal U(vi) uptake across 15+ studies consistently occurs at pH 4.5–6.0, where phosphate/amine groups remain deprotonated while uranyl hydrolysis is still limited. A significant knowledge gap emerges regarding europium and lanthanide removal: despite their importance as fission products and actinide analogs, only a handful of chitosan studies exist (*e.g.*, Abdelmonem *et al*., 144.96 mg g^−1^ for Eu(iii)), with no systematic comparison of functional group affinity (*e.g.*, –NH_2_*vs.* –COOH *vs.* –PO_4_) for trivalent f-elements. This represents a priority for future research. Abdelmonem *et al.*^91^ synthesized a chitosan/poly(acrylamide-*co*-maleic acid) hydrogel for efficient adsorption of europium isotopes ^152+154^Eu^3+^ from aqueous media. The main challenge lay in achieving high uptake capacity and selectivity for trivalent lanthanides while maintaining material stability. The hydrogel was prepared *via* gamma radiation-induced grafting of acrylamide and maleic acid onto chitosan, producing a crosslinked, porous network. Structural and thermal characterizations (FTIR, SEM, TGA, DTA) confirmed successful synthesis. Batch adsorption experiments revealed rapid initial uptake with equilibrium at ∼24 h and optimal pH ≈ 4. Adsorption followed the Langmuir model, achieving a maximum capacity of 144.96 mg g^−1^, and pseudo-second-order kinetics indicated chemisorption. Thermodynamic analysis indicated spontaneity and exothermicity, while 0.1 M HCl and AlCl_3_ solutions achieved high desorption rates. Chitosan reactive amine/hydroxyl groups improved metal ion coordination, and its biodegradability supported environmental compatibility.

**Fig. 2:**
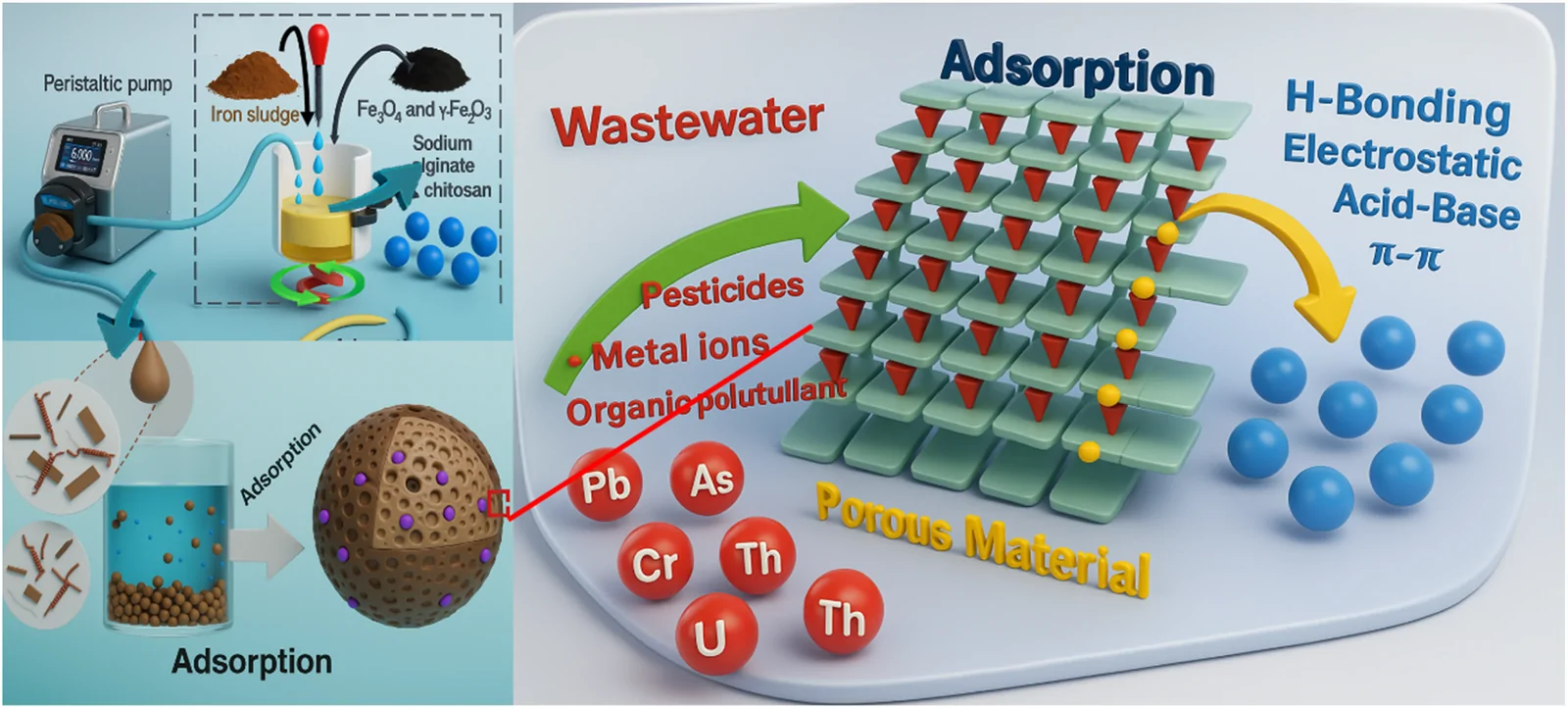
Schematic illustration showing the synthesis mechanism of magnetic alginate–chitosan foam beads with 1 : 3 ratio of magnetic nanoparticles to iron sludge (M-ACFBs_1 : 3_) and the subsequent adsorption mechanism for arsenic(v) removal from wastewater. The process involves peristaltic pump-controlled sol formation, calcium crosslinking, freeze-drying to create porous structures, and multi-mechanism contaminant adsorption including hydrogen bonding, electrostatic, acid–base, and π–π interactions, this figure has been reproduced from ref. 92 with permission from Elsevier, copyright 2022.

Fig. 2 shows that the formation mechanism of magnetic alginate-chitosan foam beads (M-ACFBs) begins with a carefully controlled synthesis process using a peristaltic pump system. In the initial stage, magnetic nanoparticles, iron sludge, sodium alginate, and chitosan are thoroughly mixed to create a homogeneous solution. This sol is then dispensed dropwise from the peristaltic pump outlet into a calcium chloride (CaCl_2_) crosslinking solution, where the beads are stabilized for 24 hours. During the crosslinking phase, calcium ions (Ca^2+^) interact with the guluronic (G) and mannuronic (M) acid units of sodium alginate *via* ion-exchange mechanisms, leading to gel formation. Simultaneously, under acidic conditions, the amino groups (–NH_2_) in chitosan become positively charged *via* protonation, forming –NH_3_^+^ groups. These positively charged chitosan groups interact electrostatically with the negatively charged carboxylate groups (–COO^−^) in sodium alginate, forming a stable double-gel network. The subsequent freeze-drying process transforms the hydrogel beads into aerogel structures, establishing three-dimensional polymer networks with highly ordered porous architecture. This porous structure significantly increases the material's specific surface area. Throughout this process, both magnetic nanoparticles and iron sludge particles become immobilized within the sodium alginate-chitosan network matrix. The resulting porous material demonstrates exceptional adsorption capabilities for wastewater treatment, effectively removing various contaminants including pesticides, metal ions (Pb, As, Cr, Th, U), and organopollutants through multiple mechanisms including hydrogen bonding, electrostatic interactions, acid–base interactions, and π–π interactions.

Broujeni *et al.*^93^ developed an efficient, reusable biosorbent for the removal of thorium(iv) ions from aqueous solutions, addressing the challenge of achieving high selectivity, capacity, and stability across varying pH conditions in radioactive wastewater treatment. The study synthesized a novel chitosan/Al_2_O_3_ nanocomposite (Ch/Al-ONCo) *via* a hydrothermal method, capitalizing on the natural biopolymer backbone of chitosan and the high surface activity of nano-alumina. The composite was characterized using XRD, BET, FT-IR, and SEM, revealing a surface area of 29.23 m^2^ g^−1^. Batch adsorption experiments examined the influence of adsorbent weight, contact time, and pH. Optimum performance was achieved at 0.2 g L^−1^ adsorbent dosage over a broad pH range (2–8), with the adsorption kinetics fitting the pseudo-second-order model and isotherms matching the Langmuir model. The maximum adsorption capacity reached 591.12 mg Th^4+^ g^−1^, achieving 99% removal efficiency at 25 °C. Desorption with 0.1 M HNO_3_ recovered over 94% of thorium, enabling multiple reuse cycles without significant capacity loss. The benefit of using chitosan lies in its abundance, biodegradability, and functional amino/hydroxyl groups that facilitate strong complexation with metal ions. At the same time, the alumina nanoparticles enhance surface activity and mechanical stability.

Zhuang *et al.*^94^ addressed the pressing issue of cobalt(ii) contamination from industrial effluents, which poses environmental and radiological hazards. The aim was to enhance chitosan adsorption performance by improving its surface area and mechanical strength, thereby overcoming the limitations of conventional chitosan beads with low surface accessibility. The researchers prepared a fibrous chitosan/cellulose composite *via* wet-spinning, yielding fibers with a diameter of 88 ± 16 µm and a specific surface area of 2.5 m^2^ g^−1^. This morphology allowed better exposure of active amino and hydroxyl functional groups. Batch adsorption studies revealed a maximum cobalt(ii) uptake of 23.6 ± 2.5 mg g^−1^ (Langmuir model), more than three times higher than that of pure chitosan beads. Optimal removal occurred at pH 6.0, with adsorption following pseudo-second-order kinetics. FT-IR and XPS analyses indicated that amino groups, assisted by hydroxyl groups, played a dominant role in metal binding *via* coordination. The benefits of using chitosan here include its natural chelating properties, environmental safety, and its adaptability to fiber processing. At the same time, the cellulose component adds structural integrity for repeated handling in practical applications.

Lin *et al.*^95^ aimed to remove uranyl–carbonate complexes from contaminated groundwater, a major challenge due to the high solubility and mobility of uranium species in carbonate-rich environments. They developed a film-like chitin/polyethylenimine (CH-PEI) biosorbent with a hierarchically porous structure and a high nitrogen content, enabling enhanced anion exchange and electrostatic attraction. The biosorbent was prepared and characterized using FT-IR, SEM-EDS, BET, and XPS. Batch adsorption experiments showed that CH-PEI reached equilibrium within 4 h at 50 mg L^−1^ uranium concentration, with maximum adsorption capacities of 247.04 mg g^−1^ at 50 mg L^−1^ and 1023.74 mg g^−1^ at 300 mg L^−1^ (Langmuir model). The optimal pH was 6.0, and removal efficiency reached 98.9% in simulated groundwater. The adsorbent-maintained performance over five regeneration cycles, demonstrating stability and reusability. The benefit of chitin lies in its biodegradability, abundance, and ability to be chemically functionalized with PEI, which introduces abundant amine groups for uranyl binding, making the system cost-effective and environmentally friendly for uranium remediation.

Liu *et al.*^96^ sought to selectively remove strontium(ii) from aqueous solutions, addressing the challenges posed by competing ions and the limited selectivity of traditional adsorbents. They synthesized a chitosan-based ion-imprinted polymer (IIP) functionalized with dithiocarbamate groups (Sr(ii)-IIP) to specifically target Sr(ii) ions. The preparation involved polymerization in the presence of Sr(ii) templates, which were later removed to create selective binding sites. Characterization by FT-IR, XPS, SEM, and nitrogen adsorption–desorption revealed spherical particles with higher surface area and porosity than raw chitosan. Batch experiments demonstrated a maximum static capacity of 86.66 mg g^−1^ at 45 °C, with fits to the pseudo-second-order and Langmuir models. Dynamic fixed-bed studies showed that adsorption increased with influent concentration but decreased with higher bed depth, flow rate, and temperature. Regeneration with minimal loss of capacity was achieved for seven cycles. The key benefit of using chitosan is its modifiable structure that allows the introduction of selective functional groups, coupled with its low cost and non-toxicity, making it a practical choice for selective radionuclide removal.

Zhang *et al.*^97^ focused on cobalt(ii) removal from aqueous solutions through biosorption, seeking an eco-friendly and economical alternative to conventional chemical treatments. They developed magnetic cyanoethyl chitosan beads, combining the magnetic separability of Fe_3_O_4_ nanoparticles with the enhanced chelation properties of cyanoethyl-modified chitosan. The beads were synthesized by grafting cyanoethyl groups onto chitosan, followed by *in situ* incorporation of magnetite. Characterization (FT-IR, SEM-EDS, XRD, TGA, BET, and VSM) confirmed the structural and magnetic properties. Adsorption studies showed optimal cobalt uptake at pH 5–8, with a maximum capacity of 17.92 mg g^−1^ (Langmuir model) and pseudo-second-order kinetic behavior. FT-IR analysis indicated that CN and NH_2_ groups were the primary binding sites. The magnetic nature of the material enabled easy separation from treated water, reducing post-treatment costs. The benefit of using chitosan is its strong affinity for transition metals, while magnetic modification provides operational convenience, making it suitable for repeated use in radioactive wastewater treatment systems.

Billah *et al.*^98^ aimed to design a high-performance adsorbent for uranyl ion recovery that integrates natural and mineral components to enhance capacity, stability, and selectivity. The challenge was to create a chitin-based system with improved adsorption sites and structural integrity while maintaining eco-friendliness. They synthesized a ternary composite of deacetylated chitin, perlite, and hydroxyapatite, combining chitin's chelating functional groups with hydroxyapatite's ion-exchange ability and perlite's mechanical stability. Structural characterization *via* FT-IR, SEM, XRD, BET, and DFT modeling confirmed the composite's enhanced surface area and porosity. Batch adsorption studies investigated the effects of concentration, temperature, pH, contact time, and adsorbent dosage, revealing a maximum adsorption capacity of 0.365 mol kg^−1^. Kinetic modeling showed that intraparticle diffusion and Elovich models best described the adsorption process, while thermodynamic analysis confirmed spontaneity and endothermicity. DFT simulations provided molecular-level insight into uranyl binding mechanisms. The benefit of chitin here is its renewable, biodegradable nature and its functional amino/hydroxyl groups, which enable complexation with uranyl ions. At the same time, the added minerals enhance durability and adsorption efficiency for repeated use.

Ao *et al.*^99^ targeted uranium(vi) removal from aqueous systems while also addressing the added challenge of imparting antibacterial activity to the adsorbent for use in environments prone to microbial growth. They developed macroporous, ultralight polyethyleneimine (PEI)-grafted chitosan/nano-TiO_2_ composite foams (PCT), synthesized variants, and identified PCT-2 as the most effective. The honeycomb macroporous structure and abundant amino/imine groups provided extensive active sites for U(vi) complexation. The adsorption process followed pseudo-second-order kinetics and Langmuir isotherm behavior, confirming chemisorption as the primary mechanism. The maximum capacity was 259.91 mg g^−1^ at pH 5.0 and 298 K, with high selectivity over competing metal ions (βU/M ranking Na^+^ > K^+^ > Mg^2+^ > Ca^2+^ > Ni^2+^ > Co^2+^ > Mn^2+^ > Al^3+^ > Fe^3+^ > Cu^2+^). Thermodynamic analysis indicated spontaneous and endothermic adsorption. The benefit of chitosan lies in its modifiable chemistry, which enables grafting with PEI and integration of TiO_2_, resulting in a lightweight, reusable, and antibacterial adsorbent ideal for radioactive wastewater treatment.

Wang *et al.*^100^ addressed the challenge of removing uranium(vi) efficiently from aqueous media under acidic conditions, where competitive ion effects are often problematic. They synthesized a composite material by coating nano-manganese dioxide (α-MnO_2_) with chitosan (α-MnO_2_@CTS), leveraging chitosan functional hydroxyl and amide groups to enhance surface reactivity. The synthesis was straightforward, producing a stable hybrid with strong uranium-binding properties. Adsorption experiments examined the influence of pH, ionic strength, coexisting ions, contact time, and temperature. Optimal removal occurred at pH 6.0 ± 0.1 and 298 K, with a maximum capacity of 326.54 mg g^−1^ fitting the Langmuir model. The adsorption mechanism combined electrostatic attraction and complexation involving hydroxyl and amide groups. Notably, the material showed minimal interference from NaNO_3_ concentration, indicating robust binding even in saline conditions. The benefit of chitosan is its biocompatibility, chemical modifiability, and ability to integrate with nanoparticles like α-MnO_2_, resulting in a cost-effective, reusable material for nuclear wastewater remediation.

Xu *et al.*^101^ aimed to selectively remove radioactive strontium(ii) from water using a chitosan-based sorbent with enhanced affinity through chemical modification. They synthesized xanthated chitosan (CTS-X) *via* a one-step process, introducing xanthate groups confirmed by XPS and FT-IR analyses. The specific surface area of CTS-X was higher than that of native chitosan, improving site accessibility. Adsorption studies revealed rapid Sr(ii) uptake, with the rate-limiting step identified as external diffusion. The maximum capacity was 58.36 mg g^−1^ (D–R isotherm model), surpassing many other chitosan-based adsorbents. Hydroxyl and xanthate groups served as primary adsorption sites, enabling strong coordination with Sr(ii). The key advantage of using chitosan is its modifiable backbone, which allows the incorporation of high-affinity functional groups, such as xanthates, resulting in efficient, selective, and low-cost radionuclide removal.

Hritcu *et al.*^102^ focused on the removal of thorium and uranyl ions from aqueous solutions using magnetic chitosan composite particles, addressing the challenge of efficiently separating and recovering loaded adsorbents without generating secondary waste. The particles, averaging 40 µm in size and with a saturation magnetization of 24 emu g^−1^, were synthesized *via* an *in situ* method that incorporated magnetite into chitosan. Characterization by SEM, EDX, FT-IR, and magnetization measurements confirmed uniform ion distribution and strong magnetic properties. Adsorption parameters were optimized for sorbent mass, ion concentration, and contact time. The composite achieved outstanding capacities 666.67 mg g^−1^ for uranyl and 312.50 mg g^−1^ for thorium under spontaneous, endothermic conditions. Magnetic separation enabled rapid recovery and regeneration for repeated cycles. The benefit of chitosan here lies in its strong chelating capacity. At the same time, magnetic integration offers a simple, efficient way to recover and reuse the adsorbent, reducing operational costs in radioactive wastewater treatment.

Schleuter *et al.*^103^ aimed to demonstrate that naturally occurring chitin networks extracted from marine sponges can serve as robust, renewable adsorbent materials for uranium recovery and to address the challenge of creating mechanically-stable, three-dimensional sorbents that can operate in aqueous environments without extensive chemical modification. The research harvested chitin from sponges of the order Verongida and processed these three-dimensional fibrous networks to obtain hydrated chitin scaffolds that retain their native macrostructure and porosity. Characterization included spectroscopic (infrared and Raman) and solid-state NMR analyses, as well as adsorption experiments to probe interactions with uranyl species. Methodologically, the authors relied on physical extraction/purification of sponge chitin followed by equilibration with uranyl solutions under controlled pH and concentration; adsorption isotherms and desorption with diluted HCl were used to evaluate capacity and reusability. The chitinous sponge networks achieved high uranium uptake, reported at about 288 mg U g^−1^, and notably permitted ∼90% desorption using diluted HCl, with no structural damage to the chitin matrices after adsorption/desorption cycles. The main benefit emphasized is that sponge-derived chitin offers a high intrinsic surface area, hierarchical porosity, and mechanical flexibility without the need for heavy chemical functionalization; it is renewable and biodegradable, and its 3D architecture eases handling and deployment (*e.g.*, cutting/pressing into shapes). The study therefore positions naturally sourced chitin networks as promising low-cost biosorbents for uranium remediation where sustainable materials are preferred. It highlights that weak interactions can nonetheless yield high uptake with reversible recovery.

Abukhadra *et al.*^104^ set out to design an eco-friendly chitosan/mesoporous silica (CH/MCM-48) nanocomposite capable of enhanced retention of U(vi) and Sr(ii) ions from both model solutions and real waters, tackling the twin challenges of boosting adsorption capacity and maintaining stability/selectivity in complex matrices. The team synthesized CH/MCM-48 by integrating chitosan chains with MCM-48 mesoporous silica under benign conditions, forming a hybrid in which chitosan provides chelating NH_2_/OH functionalities. At the same time, MCM-48 contributes high surface area and ordered porosity. Characterization employed BET surface area, FT-IR, XPS, SEM, and adsorption tests across varying pH, ionic strength, initial concentrations, and contact times. Adsorption kinetics followed pseudo-first-order behavior while equilibrium data fit a Langmuir model. The composite exhibited impressive capacities reported values of ∼261.3 mg g^−1^ for U(vi) and ∼328.6 mg g^−1^ for Sr(ii) with equilibrium reached within several hours (equilibrium ∼420 min stated). Thermodynamic analysis indicated spontaneous, favorable and exothermic interactions, and the sorbent demonstrated good recyclability and selectivity even in the presence of competing ions (Cd^2+^, Pb^2+^, Zn^2+^, Co^2+^). The benefit of chitosan in this hybrid lies in its abundant, modifiable chelating groups and biodegradability; in hybrid form with mesoporous silica, the result is a mechanically and chemically robust adsorbent with high capacity and practical reuse potential, particularly valuable for treating low-concentration radionuclide contamination in real waters.

Muzzarelli^105^ survey the potential of chitin/chitosan-bearing materials for uranium recovery across biological and engineered systems, addressing the broader challenge of assessing applicability, mechanisms and scale-up prospects. Rather than presenting a single experimental methodology, this interdisciplinary review synthesizes published data spanning isolated animal/fungal chitin, chitosan derivatives, supported biomasses and biosorption processes, and considers real-world cases and past attempts to collect uranium (including seawater studies). The review compiles evidence that chitinous materials (shells, exoskeletons, fungal biomass) can rapidly adsorb heavy metals and uranium species in some cases approaching or exceeding adsorption of twice their weight for certain metals and discusses mechanistic drivers such as chelation by amine/hydroxyl groups, surface-induced precipitation/oxide formation, and biological interactions (*e.g.*, fungal deposition of U oxides).

Recent advances demonstrate that biomass-derived and bioinspired materials offer promising pathways for efficient uranium capture through synergistic adsorption, catalytic, and structural engineering strategies.

In one approach, a vertically aligned alginate-based aerogel incorporating amidoxime-functionalized lignin and polypyrrole channels was developed for solar-enhanced uranium extraction from seawater.^106^ The engineered channel structure significantly improved photothermal conversion, mass transfer, and antifouling performance, achieving high adsorption capacity and long-term stability under natural seawater conditions. The uranium uptake was mainly driven by complexation interactions involving amidoxime, amino, and carboxyl functional groups, enabling highly selective binding under solar irradiation.

In a complementary strategy, a lignosulfonate-derived Zn-based engineered biochar was synthesized as a low-cost and scalable adsorbent for U(vi) removal from aqueous systems.^107^ The material exhibited high adsorption capacity, strong pH-dependent performance, and favorable thermodynamic behavior. The uranium capture mechanism was attributed to a combination of Lewis acid–base interactions, coordination with ZnO-derived hydroxyl groups, and complexation with oxygen- and sulfur-containing functional sites.

Furthermore, recent developments in biomass-integrated metal–organic frameworks (MOFs) highlight the importance of combining renewable biomass resources with highly porous MOF architectures.^108^ These hybrid materials overcome limitations of conventional MOFs such as poor recoverability and processability while enhancing adsorption, catalysis, and environmental applications.

Elwakeel *et al.*^109^ aimed to produce a fast-acting sorbent for uranium removal that enables facile separation and regeneration, addressing the need for operational convenience and high capacity in uranyl remediation. They prepared a magnetic chitosan resin by crosslinking chitosan with glutaraldehyde in the presence of magnetite (Fe_3_O_4_), followed by chemical modification with tetraethylenepentamine (TEPA) to introduce abundant tertiary and primary amine groups that strongly complex UO_2_^2+^. Characterization (FT-IR, SEM, magnetization) confirmed successful modification and magnetic behavior. Batch tests were conducted over pH and temperature ranges, and kinetic and thermodynamic parameters were determined. The TEPA-modified magnetic chitosan exhibited high affinity for uranyl, with a reported maximum sorption capacity of ∼1.8 mmol g^−1^ (≈425 mg g^−1^ U as UO_2_^2+^ if converting mmol→mg depending on reported basis) at pH 4 and 25 °C, and rapid kinetics. Column studies (breakthrough curves) explored flow rate, bed height, and regeneration; 0.5 M HCl enabled desorption with yields up to 98%, and the resin retained performance after several cycles. The benefit of chitosan here is twofold: (1) its reactive amino groups provide ready sites for amine grafting (TEPA) to boost chelation, and (2) embedding magnetite facilitates rapid magnetic recovery of loaded sorbent, reducing secondary solid/liquid separation steps and making the process more practical and lower-cost for real wastewater handling.

Mei *et al.*^110^ aimed to craft a mechanically resilient, highly elastic carboxymethyl chitosan (O-CMC) gel functionalized with EDTA to selectively capture Sr(ii) from water, while retaining compressive elasticity and reusability, addressing the challenge of producing adsorbents that can withstand repeated mechanical stress during deployment while providing high uptake and selectivity. Their methodology involved a one-pot reaction to produce O-CMC crosslinked and functionalized with ethylenediaminetetraacetic acid (EDTA), yielding a 3D porous gel rich in carboxyl groups distributed throughout its network. Structural characterization included porosity analysis and XPS to probe binding. Batch adsorption experiments examined kinetics and isotherms; the maximum adsorption capacity for Sr(ii) was ≈105.81 mg g^−1^ at pH 7. The gel displayed pseudo-second-order kinetics consistent with chemisorption and showed excellent mechanical resilience, with no deformation after 30 continuous compression cycles, due to multiple hydrogen bonds in the network. The O-CMC : EDTA ratio was found to be critical for balancing crosslink density and active site availability. The benefit of chitosan-derived O-CMC is clear: chitosan backbone is readily carboxymethylated to introduce negatively charged sites for divalent cation binding, while preserving biocompatibility and enabling formation of mechanically robust gels suitable for repeated compressive handling, which is advantageous for column packing, modular reactors, or mechanically stressed remediation setups.

Pavitha *et al.*^111^ sought to develop an adsorbent with exceptionally high selectivity and capacity for thorium(iv) recovery, addressing the challenge of treating radioactive wastewater efficiently while enabling regeneration and scale-up. They synthesized polymethacrylic acid-grafted chitosan-embedded silanated halloysite nanotubes (PMAA-g-CT/sHNT) by grafting PMAA onto chitosan and subsequently embedding it into surface-modified halloysite nanotubes. The combination of chitosan chelating amine/hydroxyl groups with PMAA carboxylic functionalities, along with structural reinforcement from halloysite, produced a hybrid with abundant active sites and stability. Batch adsorption tests were performed under varying conditions, and modeling was performed using the pseudo-second-order and intra-particle diffusion models. Langmuir fitting revealed a maximum Th(iv) uptake of 320.99 mg g^−1^ at 30 °C, with thermodynamic parameters indicating spontaneous and endothermic adsorption (Δ*G*° = −43.73 kJ mol^−1^, Δ*H*° = 398.71 kJ mol^−1^). Regeneration was highly efficient, with >90% recovery over four cycles. A two-stage batch reactor design was also proposed for scale-up, with an optimal dosage of 2.5 g L^−1^ for seawater simulations. The benefit of using chitosan is its adaptable chemistry for grafting functional polymers like PMAA, enabling high-capacity, selective adsorption in robust, reusable composites, especially relevant for nuclear waste management.

Tang *et al.*^112^ addressed the removal of radioactive cesium-137, aiming to combine the ion-exchange efficiency of Prussian blue analogues with the structural and chemical advantages of chitosan. They prepared a graphene oxide/chitosan hydrogel (GO/CS) with a 3D macroporous structure, then grew potassium copper hexacyanoferrate(II) (Cu-PBA) nanoparticles *in situ* to produce the GO/CS/Cu-PBA composite aerogel. This synthesis allowed Cu-PBA to be uniformly distributed and stabilized within the GO/CS network. Adsorption studies compared Cs^+^ uptake among GO, Cu-PBA, and the composite, showing that the composite achieved a capacity of 64.7 mg g^−1^ with ultrafast kinetics. Fixed-bed column tests demonstrated efficient removal from contaminated groundwater. Mechanistic studies *via* FT-IR, XRD, XPS, and EXAFS indicated Cs^+^ fixation occurred through selective ion exchange with K^+^/Cu^2+^ in the Cu-PBA lattice, accompanied by a crystalline phase transition. At the same time, GO/CS contributed electrostatic attraction *via* –COO^−^ and C–OH groups. Chitosan played a crucial role in inducing GO self-assembly into a stable, porous framework and providing additional functional sites. This bio-based support enhanced mechanical stability and recyclability, offering a cost-effective approach to Cs removal.

Elwakeel *et al.*^113^ reviewed hybrid adsorbents combining chitosan with glycidyl methacrylate (GMA) and other components for the removal of pollutants, including radioactive ions. They aimed to systematically assess preparation methods, performance factors, and modification strategies to overcome the challenges of low adsorption capacity, stability, and selectivity in unmodified chitosan. The review discusses physical blending, chemical grafting, and nanocomposite formation, highlighting how GMA integration enhances structural stability and allows for the incorporation of functional groups that target metal ions. For radioactive ions such as uranium and strontium, chitosan amino and hydroxyl groups form strong coordination bonds, while GMA-based networks enhance chemical resistance and reusability. The authors analyze the influence of operational parameters (pH, temperature, adsorbent dose, contaminant concentration) on performance and emphasize the use of kinetic models (pseudo-second-order) and equilibrium models (Langmuir/Freundlich) in data interpretation. The key benefit of chitosan, repeatedly reinforced in this review, is its renewable origin, biodegradability, and ease of modification, making it a strong candidate for hybrid systems tailored for radioactive wastewater treatment. The review also calls for more scale-up and field testing with real nuclear wastewater.

Elsayed *et al.*^114^ design an ion-imprinted amino-phenolic functionalized chitosan sorbent for the selective removal of uranyl ions (UO_2_^2+^) from aqueous effluents. The challenge addressed was poor selectivity and low adsorption rates in conventional uranium adsorbents. The synthesis involved attaching 3-hydroxy-4-nitrobenzoic acid units to chitosan *via* amide bonding, reducing nitro to amino groups, imprinting with UO_2_^2+^, and crosslinking with epichlorohydrin before template removal. The ion-imprinted polymer (U-APCS) showed a high maximum adsorption capacity (309 ± 1 mg g^−1^) and strong selectivity over competing ions. Adsorption data fit the Langmuir model, suggesting monolayer chemisorption, with optimal uptake at pH 5 and 6. Thermodynamic results indicated spontaneity and endothermicity. Chitosan functional groups played a vital role in complexation, while imprinting improved recognition sites. Benefits include reusability and environmental safety.

Humelnicu *et al.*^115^ sought to compare the adsorption performance of chitosan/clinoptilolite (CS/CPL) composite beads against crosslinked chitosan for UO_2_^2+^ and Th^4+^ ion removal from simulated radioactive solutions. The key challenge was enhancing adsorption capacity and selectivity under competitive multi-ion conditions. Beads were synthesized by incorporating clinoptilolite into chitosan and crosslinking with epichlorohydrin. Batch studies varied pH, contact time, temperature, and ion concentration. Maximum capacities were 408.62 mg g^−1^ for UO_2_^2+^ and 328.32 mg g^−1^ for Th^4+^, with kinetics following the pseudo-second-order model and isotherms best fit by the Sips model. Selectivity persisted in the presence of Cu^2+^, Fe^2+^, and Al^3+^. Regeneration using 0.1 M Na_2_CO_3_ or HCl yielded >85% recovery. Chitosan provided high-affinity sites and improved the dispersion of clinoptilolite, enhancing composite efficiency.

Alhokbany *et al.*^116^ fabricated a highly porous magnetic polymeric nanocomposite (NiFe_2_O_4_@PNC) from chitosan and 2-hydroxy-5-formylbenzoic acid for the removal of Cs(i) and Sr(ii) from water. The challenge lay in achieving high surface area, strong adsorption, and excellent regeneration. The preparation entailed forming a Schiff base polymer matrix from chitosan and an aldehyde in the presence of NiFe_2_O_4_ nanoparticles, as confirmed by FTIR, XRD, BET, SEM, TEM, and XPS analyses. The composite showed a surface area of 254 m^2^ g^−1^ and maximum adsorption capacities of 232.12 mg g^−1^ (Cs^+^) and 212.5 mg g^−1^ (Sr^2+^), fitting the Langmuir and pseudo-second-order models. Regeneration retained >92% capacity after six cycles. Chitosan biocompatibility, chelation ability, and structural flexibility enabled integration with magnetic nanoparticles for easy recovery.

Zhang *et al.*^117^ fabricated a graphene oxide-supported, phosphorylated chitosan gel bead (C-PGCB) for highly efficient uranium (U(vi)) removal from wastewater. A key challenge was enhancing uranium uptake rate and capacity under competitive-ion conditions while maintaining regeneration ability. Chitosan served as the primary biopolymer backbone, offering reactive –NH_2_ and –OH groups for chelation. It was crosslinked with phosphoric acid, introducing phosphate groups (PO and CO bonds) that increased complexation ability. Graphene oxide provided structural reinforcement and additional functional sites. The resulting C-PGCB achieved an outstanding maximum U(vi) adsorption capacity of 460.9 mg g^−1^, with an uptake efficiency of 97.1%, in just 20 minutes at an optimum pH of 5. Even with multiple coexisting ions, uranium removal remained above 90%. After five regeneration cycles using dilute acid, the beads retained >90% efficiency. Adsorption followed a Langmuir monolayer chemisorption model. Chitosan biocompatibility, low cost, and ease of chemical modification made it an ideal matrix, while phosphorylation significantly boosted uranium-binding sites. This synergy among chitosan, graphene oxide, and phosphate functionalization yielded a rapid, selective, and reusable sorbent for the treatment of radioactive wastewater.

**Fig. 3:**
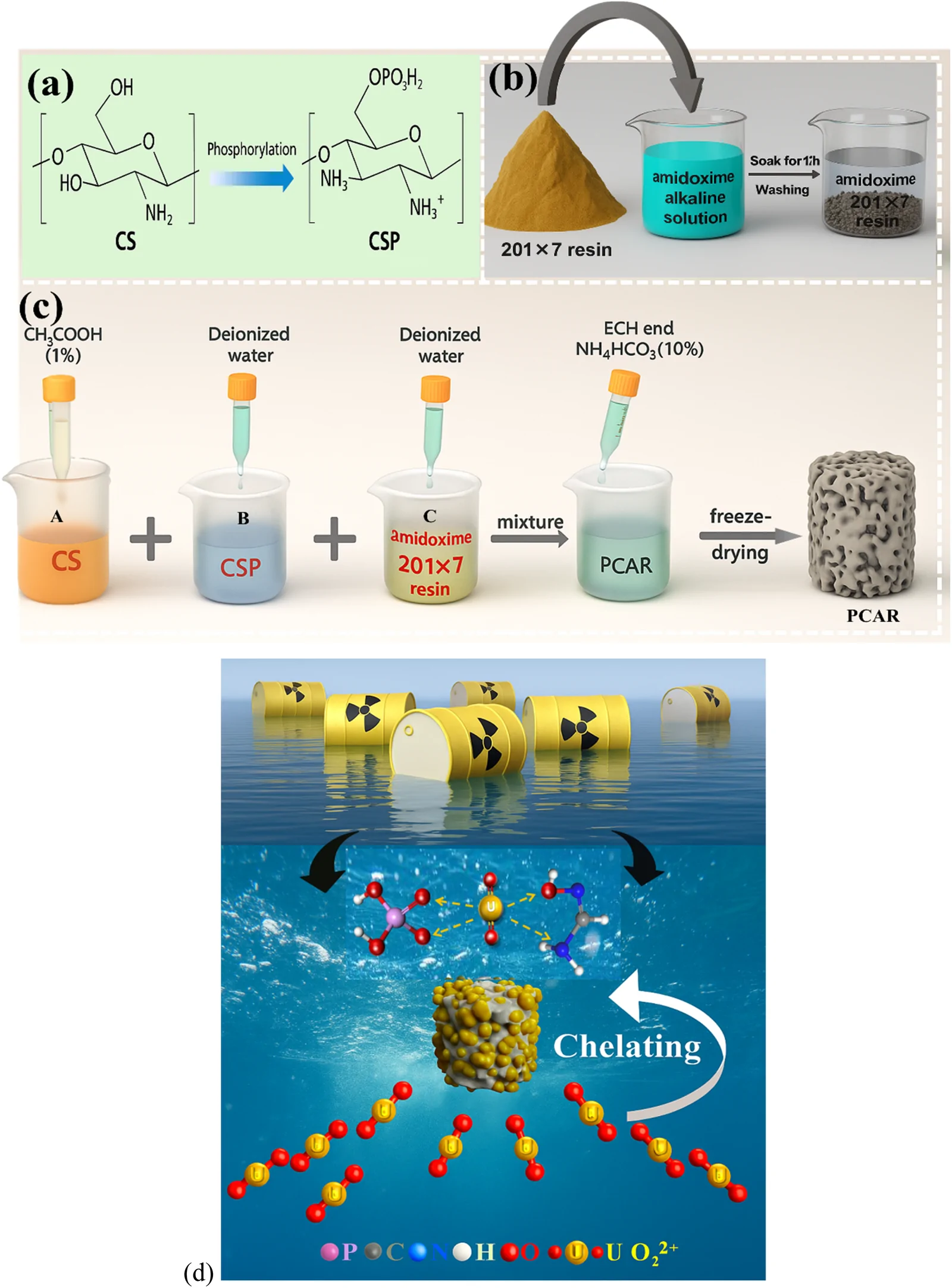
Synthesis of PCAR composite. (a) Preparation of phosphorylated chitosan (CSP) *via* methyl sulfonic acid-P_2_O_5_ phosphorylation. (b) Mixing of chitosan sol, CSP aqueous solution, and amidoxime 201 × 7 resin. (c) Formation of porous PCAR through crosslinking with epichlorohydrin, porosity generation using ammonium bicarbonate, rapid freezing, and freeze-drying. (d) Process of uranium chelation in marine environments, this figure has been reproduced from ref. 118 with permission from Elsevier, copyright 2023.

Fig. 3a illustrates the phosphorylation of chitosan (CS) to produce phosphorylated chitosan (CSP). In this process, chitosan powder is gradually added to methyl sulfonic acid (CH_4_O_3_S) under constant stirring to ensure complete dispersion and polymer chain swelling. Phosphorus pentoxide (P_2_O_5_) is then added in an ice-water bath to control the exothermic reaction and promote phosphorylation at the hydroxyl and amino sites of chitosan. This phosphorylation enhances chitosan's metal-binding capacity by introducing phosphate groups. After the reaction, the product is sequentially rinsed with ether, acetone, and methanol to remove unreacted chemicals and byproducts. The purified phosphorylated chitosan is finally obtained through freeze-drying, yielding CSP in powder form.

Fig. 3b depicts the preparation of a phosphorylated chitosan solution and its combination with amidoxime resin. CSP is dissolved in deionized water to form a homogeneous aqueous solution, while amidoxime 201 × 7 resin is separately suspended in a small volume of deionized water. In parallel, native chitosan is dissolved in dilute acetic acid to form a uniform chitosan sol. These three prepared solutions CSP solution, chitosan sol, and amidoxime resin suspension, are combined under vigorous stirring to ensure intimate mixing and coating of the resin particles with the chitosan-based components.

Fig. 3c shows the final formation of the porous phosphorylated chitosan-amidoxime resin composite (PCAR). After mixing the three components, epichlorohydrin is added as a crosslinking agent to bind the polymer chains covalently. At the same time, ammonium bicarbonate is introduced to create a porous structure through gas evolution. The reaction mixture is then rapidly frozen in liquid nitrogen to preserve the porous architecture and subsequently lyophilized for 24 hours, yielding the final PCAR material. This material combines the phosphate groups of CSP with the amidoxime functional groups of the resin, thereby enhancing the sorption efficiency of metal ions. Fig. 3d illustrates the process of uranium chelation in marine environments, showing how radioactive waste barrels containing uranium compounds can leak into ocean water. The chelation process involves specialized molecules binding to uranium atoms, forming stable complexes with the phosphorylated chitosan-amidoxime resin composite (PCAR) to effectively remove the toxic heavy metal from water and prevent its bioaccumulation in marine ecosystems.

Yang *et al.*^119^ developed a 2-phosphonate butane-1,2,4-tricarboxylic acid (PBTCA)-modified self-crosslinked chitosan gel for electrosorptive recovery of U(vi) from wastewater. The challenge was combining high adsorption capacity with low energy consumption and long-term stability. Chitosan served as the primary adsorption matrix, with abundant amino and hydroxyl groups for chelation, while self-crosslinking enhanced stability in aqueous media. PBTCA introduced strong phosphonate and carboxyl binding sites, enhancing affinity for uranyl ions. At an optimized potential of −0.6 V, the gel achieved an exceptional maximum adsorption capacity of 631.94 mg g^−1^, surpassing many reported adsorbents. The material exhibited excellent cycling stability, maintaining capacity after repeated adsorption–desorption operations. The adsorption process was governed by chemisorption, with uranyl-ligand coordination dominating the mechanism. Chitosan inherent hydrophilicity facilitated ion transport within the gel, while its chemical versatility allowed for targeted modification with PBTCA. The integration of electrosorption not only improved capacity but also reduced contact time, offering a sustainable approach to uranium recovery from complex effluents.

Salem *et al.*^120^ synthesized novel chitosan Schiff base derivatives bearing indole-linked *N*-arylacetamide moieties for the removal of U(vi) from aqueous waste. The main challenge was to increase adsorption capacity and selectivity *via* functionalization without compromising reusability. Chitosan reactive amine groups enabled condensation with indole-containing aldehydes to yield three derivatives: C-D-P, C-D-O, and C-D-B. These were characterized by FTIR, NMR, XRD, BET, zeta potential, and SEM analyses. At optimum pH = 4.0, the maximum U(vi) capacities were 272.8 mg g^−1^ (C-D-O), 214.2 mg g^−1^ (C-D-P), and 174.7 mg g^−1^ (C-D-B). The Langmuir isotherm indicated monolayer adsorption, while pseudo-second-order kinetics suggested chemisorption. Desorption with 0.5 M HCl restored >95% capacity over 5 and 6 cycles. Chitosan role was critical in providing the structural scaffold, high density of active sites, and biodegradability, while the Schiff base and indole functionalities enhanced uranyl binding *via* π–π interactions and hydrogen bonding. This synergy yielded high-capacity, reusable sorbents suitable for the remediation of uranium-contaminated water.

Wang *et al.*^121^ addressed the urgent need for efficient radioactive iodine removal by fabricating covalent organic framework/chitosan (COF/CS) aerogels. The challenge was achieving high capacity, reusability, and ease of synthesis. Chitosan served as a renewable, porous biopolymer framework, while COF nanoparticles provided high surface area and tailored adsorption sites. The aerogel was prepared *via* vacuum freeze-drying, producing a three-dimensional network with strong COF-CS integration. It exhibited remarkable adsorption capacities of 2211.58 mg g^−1^ for iodine from solution and 5.62 g g^−1^ for iodine vapor. Optimal uptake occurred rapidly due to abundant imine, amino, and aromatic groups, which interact with iodine *via* charge-transfer and van der Waals forces. After five regeneration cycles, the material retained 87% of its initial capacity. Chitosan flexible backbone supported COF dispersion, improved mechanical stability, and enhanced sorption through amine functionalities. This combination produced an efficient, green sorbent for the capture of nuclear waste iodine.

Munagapati *et al.*^122^ prepared an amine-modified chitosan (AMCS) with enhanced affinity for U(vi) ions in aqueous media. The challenge was increasing sorption capacity and selectivity while preserving chitosan environmental safety. AMCS was synthesized *via* chlorination, Schiff base formation, and amination, introducing abundant amine groups. Characterization (SEM, BET, FTIR, NMR) confirmed functionalization and surface enhancement. Under optimum conditions (pH = 5.0, dose = 0.7 g, contact time = 140 min, *C*_0_ = 50 mg L^−1^, 298 K), removal efficiency reached 94.5%, with a maximum Langmuir capacity of 287.7 mg g^−1^. Kinetics followed a pseudo-second-order model, and thermodynamic parameters (Δ*H*° = 4.698 kJ mol^−1^, Δ*S*° = 100 J mol^−1^ K^−1^, Δ*G*° < 0) indicated spontaneity and endothermicity. After multiple regenerations, capacity remained >75%. Chitosan base structure provided an easily modifiable platform, while amination significantly boosted U(vi) coordination *via* chelation, making AMCS a promising adsorbent for radioactive waste treatment.

Cha *et al.*^123^ targeted the removal of radioactive iodine (I^−^) using a silver-incorporated bentonite-chitosan hydrogel (CBA). The challenge was designing a robust, selective sorbent that maintained performance in varying pH and ion-rich environments. Chitosan served as the main biopolymer matrix, contributing abundant amino and hydroxyl groups for ion interaction. At the same time, bentonite provided layered silicate adsorption sites, and silver enhanced iodine binding *via* precipitation and complexation. Bentonite-Ag composites were synthesized *via* ion exchange and then embedded in chitosan to form hydrogels. The optimized CBA50 hydrogel achieved a maximum adsorption capacity of 21.6 mg g^−1^ (Langmuir model) and kinetics that fit the pseudo-second-order model, indicating chemisorption. The hydrogel performed effectively across pH 4–11 and in the presence of competing anions and cations, demonstrating robustness. Regeneration over five cycles retained high removal efficiency. Chitosan role was crucial: it provided a flexible, hydrophilic support for uniform Ag-bentonite dispersion, improved structural integrity, and enhanced binding through amine–iodine interactions. This synergy enabled stable, reusable iodine removal for radioactive wastewater treatment.

Chen *et al.*^124^ enhanced U(vi) adsorption by grafting 1,3-dicarbonyl moieties onto chitosan, incorporating them into phytic acid-sodium alginate, and doping with tungsten oxide (WO_3_). The challenge was creating chemically stable, porous beads with high uranium specificity. Chitosan reactive backbone enabled ethyl acetoacetate grafting *via* epichlorohydrin mediation, improving selectivity. The WO_3_-doped composite (MCPS-3) achieved a maximum Langmuir capacity of 343.85 mg g^−1^ at pH 4.5 and 310 K, with pseudo-second-order kinetics (*R*^2^ ≈ 0.99) indicating chemisorption. Thermodynamic data (Δ*H*° = 30.51 kJ mol^−1^, Δ*G*° ≈ −27 kJ mol^−1^) confirmed a spontaneous, endothermic process. WO_3_ enhanced mechanical stability and surface area, while phytic acid provided phosphate sites for strong uranyl coordination. Chitosan abundant amino and hydroxyl groups were essential for binding, and its biopolymeric nature ensured environmental safety. This advanced hybrid offered excellent reusability and high efficiency for uranium recovery from nuclear effluents.

Wang *et al.*^125^ fabricated ion-imprinted, macroporous polyethyleneimine (PEI) incorporated chitosan/layered hydrotalcite foams (IPCL) for selective U(vi) adsorption. The challenge was improving uranium selectivity in complex waters. Chitosan formed a macroporous foam structure *via* freeze-drying, with PEI enhancing amino-group density and hydrotalcite providing layered-hydroxide adsorption sites. Ion-imprinting created uranyl-shaped cavities, doubling selectivity. The IPCL-2 composite exhibited a maximum U(vi) capacity of 278.8 mg g^−1^ at pH 5.0, 298 K, and 2 h contact time, nearly twice that of non-imprinted controls. Adsorption followed the Freundlich model and pseudo-second-order kinetics, indicating heterogeneous chemisorption. Thermodynamics confirmed that the process is spontaneous and endothermic. Chitosan was indispensable for forming the lightweight, stable foam and providing reactive sites for complexation, while imprinting amplified its molecular recognition for U(vi). The material was reusable, making it ideal for the remediation of radioactive wastewater.

Huang *et al.*^126^ produced polyethyleneimine-modified chitosan composite microspheres (PEI/ECH-CTS) for selective U(vi) removal from radioactive nuclear waste. The challenge was maximizing adsorption while retaining reusability. Chitosan microspheres, formed *via* epichlorohydrin crosslinking, provided a robust, spherical structure with high accessibility of functional groups. PEI modification increased nitrogen content to 6.57 mol kg^−1^, greatly enhancing uranyl coordination. The 0.4-PEI/ECH-CTS variant achieved a maximum capacity of 380.65 mg g^−1^ within 4 h and 100% removal efficiency from simulated nuclear wastewater, reducing U(vi) from 10.98 mg L^−1^ to 1 µg L^−1^ (below US EPA drinking water limit of 30 µg L^−1^). Kinetics followed pseudo-second-order kinetics, and selectivity exceeded that of competing ions by up to 6.1 × 10^4^. After seven cycles, the adsorption efficiency remained above 95%. Chitosan structural stability, hydrophilicity, and modifiability were key to achieving these outstanding results.

Yang *et al.*^127^ developed a photothermal composite aerogel integrating Prussian blue analogues (PBAs) and straw biochar into a chitosan/waste leather scrap hydrolysate (CS/WLSH) matrix for simultaneous removal of Cs^+^ and Sr^2+^ from nuclear wastewater. The challenge was to accelerate adsorption kinetics under mild conditions. Chitosan provided a porous, hydrophilic framework with active amino and hydroxyl groups, enhancing both structural integrity and ion-binding capacity. PBAs targeted Cs^+^ through lattice trapping, while the aerogel biochar component enhanced light absorption for photothermal conversion. Under simulated sunlight, equilibrium was reached in 2 h compared to 5 h in the dark, with maximum adsorption capacities of 156.0 mg g^−1^ for Cs^+^ and 95.1 mg g^−1^ for Sr^2+^ at neutral pH. The material performed well even in simulated seawater and retained reusability over multiple cycles. Chitosan biodegradability, chemical modifiability, and porosity made it a critical backbone for functional integration, enabling fast, selective adsorption enhanced by solar energy. Chitosan structure allowed efficient dispersion of PBAs and biochar, combining mechanical stability with high ion affinity for sustainable radioactive ion recovery.

Zhou *et al.*^128^ designed honeycomb porous l-cysteine bridged chitosan/Mg–Al hydrotalcite (CLM) composite foams for U(vi) biosorption. The challenge was achieving high selectivity and capacity in multicomponent solutions. Chitosan provided the main framework with abundant reactive groups, while l-cysteine introduced thiol and carboxyl functionalities for stronger uranyl binding. Mg–Al hydrotalcite contributed layered adsorption sites. CLM-2 exhibited a maximum U(vi) capacity of 262.23 mg g^−1^ at pH 5.0 and 298 K, fitting the Freundlich isotherm and pseudo-second-order kinetics. Thermodynamic analysis confirmed spontaneous and endothermic adsorption. The honeycomb pore structure facilitated mass transfer, and multigroup coordination (amino, thiol, hydroxyl, carboxyl) improved uptake efficiency. The composite maintained performance in complex wastewater. Chitosan flexible matrix supported pore formation and functionalization, enabling high-efficiency, selective U(vi) recovery with structural durability.

Hizal *et al.*^129^ introduced humic acid (HA) embedded chitosan/polyvinyl alcohol (Ch/PVA-HA) composites for uranyl ion removal. The challenge was to increase adsorption capacity while reducing costs. Chitosan served as a biodegradable, functional backbone, and HA enhanced binding *via* its aromatic and carboxylic groups. The composite achieved a maximum batch capacity of 37.8 mg g^−1^ at pH 1–4 and an impressive column capacity of 1909 mg g^−1^, outperforming Ch/PVA without HA. Surface area increased from 0.254 m^2^ g^−1^ (Ch/PVA) to 1.308 m^2^ g^−1^ (Ch/PVA-HA). Adsorption shifted from the Freundlich to the Langmuir behavior with HA modification, indicating monolayer chemisorption. The composite showed good reusability with minimal loss of performance. Chitosan amino groups facilitated HA integration, enhancing surface area and reactivity, making it a cost-effective, high-capacity sorbent for uranium recovery.

Zhou *et al.*^130^ fabricated honeycomb-like macroporous crosslinked chitosan foams intercalated with EDTA-modified Ca–Mg–Al layered hydrotalcite (CS-EDTA-LDH) for U(vi) biosorption. The challenge was to increase binding site density while preventing structural collapse. Chitosan porous structure provided a scaffold, while EDTA introduced strong chelation sites. The composite achieved a maximum U(vi) capacity of 272.3 mg g^−1^ at pH 5.0 and 298 K, following the Freundlich isotherm and pseudo-second-order kinetics. Thermodynamics indicated a spontaneous, endothermic process. Mechanisms included complexation by EDTA and chitosan, ion exchange, and isomorphic substitution. The foam ultralight nature allowed rapid adsorption and easy recovery. Chitosan structural stability and reactivity made it an ideal host for EDTA-intercalated LDH, boosting uranium binding and operational practicality.

Huang *et al.*^131^ developed amino-rich chitosan aerogels *via* a one-step crosslinking method for selective adsorption of U(vi) from radioactive nuclear wastewater. The challenge was to combine high adsorption efficiency, selectivity, and cost-effectiveness while ensuring compliance with strict water safety limits. Chitosan was first carboxylated to improve water solubility and then modified with polyethylene polyamine (PEPA) and polydopamine (PDA), which increased amine group density and reduced steric hindrance. The optimized aerogel, PEPA_2_._0_/PDA_0_._7_-CMCS_3_._5_–250, achieved a maximum adsorption capacity of 467.73 mg g^−1^ and reduced U(vi) concentration from 12.35 mg L^−1^ to 10 µg L^−1^, below China drinking water limit of 30 µg L^−1^. Adsorption followed pseudo-second-order kinetics and exhibited high selectivity over competing ions. Regeneration maintained performance over multiple cycles. Chitosan modifiable backbone allowed high-density amination and stable aerogel formation, resulting in rapid, selective uranium removal that meets drinking water safety standards.

He *et al.*^132^ repurposed waste medical masks into acid-modified activated carbon grafted with chitosan for U(vi) removal. The challenge was to create a high-capacity, selective sorbent from waste materials. Waste masks were converted to activated carbon, treated with sulfuric acid to introduce –SO_3_H groups, and then chitosan was grafted to enhance functional diversity. The resulting CSMA composite achieved a maximum adsorption capacity of 467.93 mg g^−1^ at pH 7, 0.1 g L^−1^ dose, 6 h contact time, and 303 K, following Langmuir isotherm and pseudo-second-order kinetics. The process was spontaneous and endothermic. Selectivity tests confirmed a strong preference for U(vi) over coexisting ions, and performance remained high after several regeneration cycles. Chitosan enabled strong uranyl coordination *via* amine and hydroxyl groups while increasing sustainability by converting waste into a high-performance sorbent.

Fadel *et al.*^133^ created O-carboxymethyl chitosan (O-CMC) loaded with nano copper potassium hexacyanoferrate (CuKHCF) for Cs^+^ removal. The challenge was achieving high selectivity in both batch and column systems. O-CMC introduced carboxyl groups for ion exchange, while CuKHCF provided strong lattice entrapment of Cs^+^. Batch tests showed a maximum capacity of 1.71 mmol g^−1^ (≈224 mg g^−1^) at pH 7, 2 h contact time, 25 °C, fitting Langmuir and pseudo-second-order models. Column studies confirmed efficient removal at various bed depths and flow rates, with regeneration using 2 M KCl restoring performance over five cycles. Chitosan functional modification enhanced nanoparticle dispersion and active site density, enabling selective, reusable cesium capture.

Majdoubi *et al.*^134^ introduced a geopolymer-chitosan composite (Ch@GPol) synthesized from oil shale for U(vi) adsorption. The challenge was enhancing adsorption while maintaining structural stability in aqueous systems. Chitosan was integrated into the geopolymer to increase surface reactivity and introduce amino/hydroxyl sites for uranyl binding. The composite showed a maximum adsorption capacity of 0.236 mol kg^−1^ (∼56 g kg^−1^) at natural pH, with pseudo-second-order kinetics indicating chemisorption. Thermodynamic analysis confirmed spontaneity, and DFT simulations revealed strong binding interactions between uranyl and chitosan functional groups. Increased porosity and improved surface charge distribution were observed due to chitosan incorporation. Chitosan provided reactive coordination sites and enhanced porosity, creating a stable, high-performance hybrid for uranium remediation.

Ren *et al.*^135^ developed chitosan-based hydrogels incorporated with polyethylenimine (PEI) and reduced graphene oxide (rGO) for U(vi) removal from nuclear effluents. The challenge was enhancing adsorption kinetics and structural robustness in aqueous systems. Chitosan amino and hydroxyl groups acted as primary uranium binding sites, while PEI increased nitrogen content and rGO added mechanical strength and high surface area. The optimized hydrogel achieved a maximum adsorption capacity of 392.15 mg g^−1^ at pH 5, 298 K, and a contact time of 120 min, following the Langmuir isotherm and pseudo-second-order kinetics. The adsorption was spontaneous (Δ*G*° < 0) and endothermic (Δ*H*° = 28.4 kJ mol^−1^). The hydrogel maintained >90% efficiency after five regeneration cycles using 0.1 M HCl. Chitosan provided the flexible, reactive framework necessary for high uranyl binding and stable composite formation, enabling rapid and reusable uranium removal.

Table 1 summarizes studies on chitosan-based materials for the removal of radioactive contaminants, highlighting their diverse applications, high adsorption capacities, and key functional advantages. Chitosan composites target a wide range of ions, including U(vi), Cs^+^, Sr^2+^, Th(iv), and Eu(iii), with maximum capacities reaching up to 690 mg g^−1^ for U(vi) (CuHCF/O-CMCs) and 2211.58 mg g^−1^ for iodine (COF/chitosan aerogel). Most materials operate optimally at pH 4–7, leveraging chitosan amino groups for ion binding. Functionalization strategies such as cross-linking with EDTA, grafting with Schiff bases, or embedding magnetic nanoparticles enhance selectivity, reusability, and performance in complex environments. Notably, chitosan biocompatibility, biodegradability, and compatibility with waste-derived materials (*e.g.*, recycled masks, oil shale) underscore its sustainability. These innovations demonstrate chitosan versatility as a low-cost, efficient, and eco-friendly solution for the remediation of radioactive waste.

**Table 1:** Summarizes studies on chitosan-based materials for the removal of radioactive contaminants, highlighting their diverse applications, high adsorption capacities, and key functional advantages

Material type	Target ion(s)	Optimum pH	Maximum capacity (mg g^−1^)	Key benefit of using chitosan	Reference
Polysaccharide-based gel (chitosan)	Radioactive cations	Not specified	Not specified	Forms strippable coatings for easy removal of contaminants; biocompatible and biodegradable	136
Magnetic bentonite-chitosan beads (Bn-CTS)	Cs^+^	Not specified	57.1	High adsorption capacity and magnetic separation for recycling	90
Chitosan/poly(acrylamide-*co*-maleic acid) hydrogel	^152+154^Eu(iii)	∼4	144.96	Radiation-synthesized; high selectivity for lanthanides	137
Ion-imprinted amino-phenolic chitosan (U-APCS)	UO_2_^2+^	5 and 6	309	Selective recognition of uranyl ions *via* ion-imprinting	114
Chitosan/clinoptilolite composite (CS/CPL)	UO_2_^2+^, Th^4+^	Not specified	408.62 (U), 328.32 (Th)	Enhanced adsorption *via* composite with clinoptilolite; reusable	115
Chitosan@DOTAGA hydrogel	Metals (wound)	Not specified	Not specified	Chelates metals in wounds; combines chitosan biocompatibility with DOTAGA binding	138
Magnetic chitosan-Schiff base nanocomposite	Cs(i), Sr(ii)	Not specified	232.12 (Cs), 212.5 (Sr)	High porosity and magnetic separation; reusable	116
Phosphorylated chitosan/graphene oxide gel bead (C-PGCB)	U(vi)	Not specified	460.9	Rapid uptake (20 min) and high selectivity in multi-ion systems	117
PBTCA-modified chitosan gel	U(vi)	Not specified	631.94	Electrosorption at low energy; high regeneration capability	119
Chitosan-Schiff base with indole groups	U(vi)	4.0	272.8	High selectivity and desorption efficiency	120
COF/chitosan aerogel	Iodine	Not specified	2211.58 (solution), 5.62 (vapor)	3D porous structure; excellent recyclability	121
Amine-modified chitosan (AMCS)	U(vi)	5.0	287.7	Multi-step functionalization enhances U(vi) affinity	139
Chitosan-bentonite-silver hydrogel (CBA)	Iodide	4–11	21.6	Broad pH tolerance; reusable	123
Chitosan-vermiculite-lignin composite (Ch-VL)	UO_2_^2+^	4.5	600	Cost-effective; spontaneous/endothermic adsorption	140
WO_3_-doped chitosan/alginate composite (MCPS-3)	U(vi)	4.5	343.85	Synergy of WO_3_ and phosphate-rich alginate for high capacity	141
PEI-incorporated chitosan/hydrotalcite foam (IPCL)	U(vi)	5.0	278.8	Macroporous structure; ion-imprinting enhances selectivity	142
PEI-modified chitosan microspheres (0.4-PEI/ECH-CTS)	U(vi)	Not specified	380.65	High nitrogen content; reduces U(vi) to safe drinking levels	126
Chitosan aerogel with Prussian blue analogs (PBAs)	Cs^+^, Sr^2+^	Not specified	156.0 (Cs), 95.1 (Sr)	Solar-driven photothermal adsorption; multifunctional	127
l-cysteine bridged chitosan/hydrotalcite foam (CLM-2)	U(vi)	5.0	262.23	Honeycomb pores; synergistic coordination with multiple functional groups	143
Humic acid-embedded chitosan/PVA composite (Ch/PVA-HA)	UO_2_^2+^	1–4	1909 (column)	High column capacity; cost-effective	129
EDTA-intercalated chitosan/hydrotalcite foam	U(vi)	5.0	272.3	EDTA enhances complexation; macroporous for rapid adsorption	144
CuHCF/O-CMCs nanocomposite	U(vi)	4.0	690	Nano-sized hexacyanoferrate for high capacity; reusable	145
PEPAx/PDAy-CMCS aerogel	U(vi)	Not specified	467.73	One-step cross-linking; reduces U(vi) to ultra-trace levels	131
Acid-modified mask-based chitosan composite (CSMA)	U(vi)	7.0	467.93	Waste mask recycling; high selectivity in complex solutions	132
PMAA-g-CT/sHNT composite	Th(iv)	Not specified	320.99	High reusability (>90% recovery); designed for batch reactors	146
CuKHCF/O-CMC nanocomposite	Cs^+^	7.0	1.71 mmol g^−1^	High selectivity for Cs^+^; reusable	133
Chitosan-oil shale geopolymer (Ch@GPol)	U(vi)	Natural pH	0.236 mol kg^−1^	Combines chitosan with a geopolymer to enhance porosity	134
EDTA-functionalized chitosan	Nd, Dy	Not specified	85.3% (Nd), 76.9% (Dy)	Recovers REEs from e-waste; eco-friendly	147
Nanostructured chitosan/MS-4A (NSC@MS-4A)	Cs^+^, Sr^2+^	6 and 7	92–94% removal	Fast adsorption; reusable for radioactive wastewater	148

Liu *et al.*^149^ aimed to design polyethylenimine/reduced graphene oxide (PEI/rGO) reinforced chitosan hydrogels for efficient U(vi) removal. The challenge was achieving both high adsorption capacity and mechanical stability under aqueous conditions while fully exploiting chitosan amino (-NH_2_) and hydroxyl (−OH) functional groups. Chitosan was dissolved in dilute acetic acid, crosslinked with glutaraldehyde, and combined with PEI (rich in –NH_2_ groups) and rGO to enhance structural robustness and surface area. The amino groups from both chitosan and PEI provided abundant coordination sites for uranyl ions, while hydroxyl groups enhanced hydrogen bonding and ion exchange. At pH 5.0, 298 K, and 120 min contact time, the hydrogel achieved a maximum adsorption capacity of 392.15 mg g^−1^, and the Langmuir and pseudo-second-order kinetic models fit the data well. Thermodynamic analysis showed an endothermic process (Δ*H*° = 28.4 kJ mol^−1^) and spontaneous adsorption (Δ*G*° < 0). After five regeneration cycles using 0.1 M HCl, >90% efficiency was retained. Chitosan native amino and hydroxyl groups synergistically enhanced uranyl ion coordination, while composite reinforcement improved reusability and stability.

Lefatle *et al*.^150^ produced magnetic chitosan/zeolite nanocomposites (MCS/Z) for uranium recovery, targeting rapid separation and reuse. The challenge was integrating magnetic properties without reducing the binding efficiency of chitosan amino and hydroxyl groups. Chitosan was crosslinked with glutaraldehyde in the presence of Fe_3_O_4_ nanoparticles and zeolite. Amino groups provided strong coordination to U(vi), hydroxyl groups contributed to hydrogen bonding, and zeolite enhanced ion exchange. The composite achieved a maximum U(vi) adsorption of 288.5 mg g^−1^ at pH 5.0 and 298 K, reaching equilibrium in 60 min. Magnetic separation efficiency was >98%, and regeneration with 0.1 M HCl retained >90% capacity over five cycles. Chitosan amino/hydroxyl groups ensured high binding capacity, while magnetization enabled rapid recovery without filtration.

Şenol *et al.*^140^ developed a chitosan–vermiculite–lignin composite for UO_2_^2+^ adsorption, aiming to create a hybrid biosorbent that leveraged the abundant amino and hydroxyl groups of chitosan alongside the high ion-exchange capacity of vermiculite and the structural stability from lignin. The main challenge was combining these components into a mechanically strong yet highly functional adsorbent. Chitosan was dissolved in dilute acetic acid, mixed with pretreated vermiculite and lignin, and then freeze-dried to produce a porous structure. The amino and hydroxyl groups in chitosan provided primary uranyl-binding sites, supported by vermiculite cation exchange and lignin aromatic hydroxyls. The composite achieved a remarkable maximum capacity of 600.0 mg g^−1^ at pH 4.5, with adsorption equilibrium in 60 minutes. The isotherm followed the Langmuir model, and desorption with 0.1 M HCl allowed over 95% recovery after multiple cycles. The incorporation of chitosan functional groups was critical for the high capacity, as they directly participated in chelation and hydrogen bonding with uranyl ions, enabling exceptional performance even at low pH.

## 5. Alginate-based materials for radioactive ion capture

Alginate, extracted from brown seaweed (Phaeophyceae), occupies a distinctive position among polysaccharide adsorbents. Its molecular structure consists of linear copolymers of β-d-mannuronate (M) and α-l-guluronate (G) residues arranged in blocks. The G-block regions possess a unique property: they form cooperative “egg-box” complexes with divalent and trivalent cations *via* interchain crosslinking between adjacent G residues. This mechanism, well-established in alginate hydrogel formation with Ca^2+^, extends to radionuclides such as Sr^2+^, Ba^2+^, and UO_2_^2+^. Three features make alginate particularly attractive for nuclear waste treatment: (1) Each uronate residue carries a –COO^−^ group (p*K*_a_ ∼3.5), providing abundant negatively charged binding sites across a broad pH range (4–10). (2) Alginate forms stable hydrogels upon contact with divalent cations (Ca^2+^, Ba^2+^, Sr^2+^) without the need for toxic crosslinkers or organic solvents, a significant green chemistry advantage. (3) Alginate readily incorporates inorganic nanoparticles, clays, MOFs, and other functional materials while maintaining its gel structure.

However, alginate has three well-recognized weaknesses: (i) poor mechanical stability in acidic media (pH < 3) due to protonation of carboxylates and loss of crosslinking, (ii) gradual degradation in high-phosphate or high-citrate solutions due to competitive Ca^2+^ chelation, and (iii) lower affinity for monovalent cations (Cs^+^, TcO_4_^−^) compared to divalent/trivalent species.

A critical observation across the literature is that alginate's adsorption performance is highly sensitive to the choice of crosslinking cation. Ca^2+^-crosslinked alginate is standard but can exchange with target radionuclides, leading to both adsorption (desired) and gradual disintegration (undesired). Ba^2+^ crosslinking produces more stable beads but introduces an additional heavy metal. This trade-off is rarely discussed explicitly.

Yang *et al.*^151^ fabricated WO_3_-doped chitosan/phytic acid/alginate composite beads to combine the uranyl-binding advantages of alginate carboxyl groups with chitosan amino groups and the oxophilic properties of tungsten trioxide. The challenge was achieving high affinity and stability in acidic conditions. Sodium alginate, chitosan, and phytic acid were blended, crosslinked with Ca^2+^, and doped with WO_3_ nanoparticles. At pH 4.5, the beads exhibited a maximum U(vi) capacity of 343.85 mg g^−1^, reaching equilibrium within 120 minutes. Alginate contribution was essential, providing a hydrophilic matrix with abundant carboxyl sites for ion exchange, complementing chitosan nitrogen coordination and WO_3_ affinity for oxygen-donor ligands. FTIR and XPS confirmed multi-site binding involving –COOH, –NH_2_, and phosphate groups. The material retained over 90% capacity after five regenerations with 0.1 M HCl.

Hong *et al.*^152^ prepared alginate/zeolite composite foams for uranium adsorption, combining alginate biopolymer matrix with zeolite ion exchange capability. The challenge was to fabricate a porous, lightweight adsorbent with high mechanical stability and uranium affinity. Sodium alginate was mixed with zeolite particles, crosslinked with Ca^2+^, and freeze-dried to form open-cell foams. At pH 5.0, the composite exhibited a maximum U(vi) capacity of 255.0 mg g^−1^ with equilibrium achieved in 90 minutes. Alginate carboxyl groups directly chelated uranyl ions and also facilitated the dispersion of zeolite particles within the foam. The material retained over 88% adsorption efficiency after five acid regenerations, confirming its reusability and the structural integrity provided by the alginate framework.

Kazemi and Javanbakht^153^ produced magnetic alginate/zeolite nanocomposites (MAlg/Z) for U(vi) recovery, aiming to combine alginate –COOH–rich chelating ability with zeolite cation exchange and magnetic separation for easy handling. The challenge was to embed Fe_3_O_4_ nanoparticles without compromising adsorption performance. Sodium alginate was mixed with zeolite and Fe_3_O_4_ nanoparticles, then gelled in CaCl_2_ to form magnetic beads. At pH 5.0, the composite achieved a maximum capacity of 288.5 mg g^−1^, reaching equilibrium within 60 minutes. Alginate played a critical role by maintaining porosity, enabling ion exchange, and providing stable uranyl binding sites. Magnetic recovery efficiency exceeded 98%, and the material retained >90% of its capacity after 5 regeneration cycles with 0.1 M HCl, demonstrating its potential for sustainable uranium recovery.

Pavithra *et al.*^146^ developed alginate-poly(methacrylic acid) grafted halloysite nanotube (Alg-PMAA-HNT) composites for Th(iv) recovery, aiming to harness alginate high carboxyl group density and PMAA additional –COOH functionalities for enhanced tetravalent ion binding. The challenge was achieving strong adsorption while maintaining mechanical stability in aqueous media. Sodium alginate was mixed with PMAA-grafted HNTs and crosslinked with Ca^2+^ to form composite beads. At pH 6.0, the material exhibited a maximum Th(iv) capacity of 320.99 mg g^−1^, with equilibrium reached in 90 minutes, as described by the Langmuir and pseudo-second-order models. Alginate role was pivotal, providing a hydrophilic network and abundant chelating sites that synergized with PMAA carboxyl groups for strong coordination complexes. The beads maintained over 92% capacity after five regeneration cycles with dilute HCl, underscoring the durability of the alginate framework.

Ammar *et al.*^145^ developed Cu-hexacyanoferrate (CuHCF)-encapsulated O-carboxymethyl alginate beads (CuHCF@O-CMA) for highly efficient U(vi) adsorption, aiming to combine the ion-exchange capacity of CuHCF with alginate biopolymer chelation. The challenge was dispersing CuHCF nanoparticles evenly within the alginate matrix without blocking active –COOH sites. Sodium alginate was carboxymethylated and used to encapsulate CuHCF nanoparticles *via* Ca^2+^ gelation, yielding robust beads. At pH 4.0, the beads achieved an exceptional maximum capacity of 690.0 mg g^−1^, with column breakthrough remaining stable above 600 mg g^−1^. Alginate hydrophilic network stabilized CuHCF particles, while –COOH groups provided additional uranyl binding, enhancing overall uptake. Regeneration with 0.1 M HNO_3_ retained 95% capacity after five cycles, illustrating that alginate dual role as stabilizer and chelator was key to performance.

Momin *et al.*^154^ synthesized alginate/metal–organic framework composite beads (Alg/MOF) for U(vi) adsorption, targeting the combination of MOF large surface area with alginate chelating matrix. The challenge was to embed MOF crystals without collapse or leaching under aqueous conditions. Sodium alginate was mixed with pre-synthesized MOF particles and gelled in CaCl_2_ solution, producing mechanically stable beads. At pH 5.0, the beads reached a maximum capacity of 278.2 mg g^−1^, achieving equilibrium in 80 minutes. Alginate carboxyl groups facilitated direct uranyl chelation, while the hydrogel framework prevented MOF aggregation, thereby preserving adsorption efficiency. The beads retained >90% capacity after five regenerations, highlighting alginate stabilizing function.

Yang *et al.*^155^ created cerium-alginate beads (Ce-Alg) for selective adsorption of U(vi) and Th(iv), aiming to utilize the affinity between Ce^3+^ sites and actinide oxycations in combination with alginate –COOH groups. The challenge was to load Ce ions effectively without impairing gelation. Sodium alginate was preloaded with Ce^3+^, then crosslinked with Ca^2+^ to form dual-metal beads. At pH 5.5, U(vi) and Th(iv) capacities reached 295.3 mg g^−1^ and 288.7 mg g^−1^, respectively, with good selectivity over competing ions. Alginate binding groups complemented Ce coordination sites, enhancing both affinity and stability. After five regeneration cycles, over 93% adsorption efficiency was maintained.

Qing *et al.*^156^ fabricated alginate-cellulose nanofiber (Alg-CNF) aerogels for U(vi) adsorption, aiming to integrate alginate functional groups with CNF high surface area and mechanical strength. The challenge was creating an interconnected, porous aerogel while preserving alginate binding sites. Sodium alginate and CNF were mixed, freeze-dried, and crosslinked with Ca^2+^. At pH 5.5, the aerogel exhibited a maximum capacity of 283.6 mg g^−1^ and achieved equilibrium in 75 minutes. Alginate –COOH groups provided strong uranyl coordination, while CNF maintained structural integrity for repeated use. After five regenerations, capacity retention exceeded 92%.

Wang *et al.*^157^ designed alginate-coated bentonite composites (Alg-Bent) for U(vi) adsorption, combining bentonite cation exchange with alginate chelating carboxyl groups. The challenge was coating bentonite uniformly while avoiding pore blockage. Bentonite particles were suspended in sodium alginate solution, then crosslinked with Ca^2+^ to form stable beads. At pH 5.0, the composite achieved a maximum U(vi) capacity of 247.8 mg g^−1^ with equilibrium in 80 minutes. Alginate-stabilized bentonite dispersion increased hydrophilicity and provided additional active sites for uranyl binding. The material retained >91% adsorption efficiency after five regeneration cycles.

Zhu *et al.*^158^ prepared polyacrylonitrile/alginate composite fibers (PAN/Alg) for U(vi) adsorption, aiming to integrate alginate hydrophilicity and carboxyl groups into a fibrous adsorbent for easy handling. The challenge was achieving a uniform alginate coating on PAN fibers. PAN fibers were coated with sodium alginate and crosslinked with Ca^2+^. At pH 5.0, the fibers exhibited a maximum capacity of 265.8 mg g^−1^, with equilibrium in 70 minutes. Alginate significantly improved uranyl binding and wettability, enabling faster uptake. After five regeneration cycles with dilute HCl, the fibers maintained >91% capacity, confirming the durability of the alginate layer.

Tayyebi *et al.*^159^ fabricated alginate-bentonite aerogels for U(vi) removal, aiming to merge bentonite ion exchange with alginate chelation in a lightweight, porous structure. The challenge was to maintain aerogel porosity while achieving uniform bentonite dispersion. Sodium alginate and bentonite were mixed, frozen, freeze-dried, and crosslinked with Ca^2+^. At pH 5.0, the aerogel showed a maximum capacity of 288.6 mg g^−1^, with equilibrium in 85 minutes. Alginate stabilized the clay particles, prevented aggregation, and provided supplementary binding through –COOH groups. After five regeneration cycles, the adsorption efficiency remained above 90%.

Dong *et al.*^160^ created alginate-tannic acid hydrogel beads (Alg-TA) for U(vi) adsorption, aiming to combine alginate –COOH sites with tannic acid phenolic hydroxyl groups for multi-site binding. The challenge was preventing TA leaching in aqueous systems. Sodium alginate and TA were mixed, then gelled in Ca^2+^ to form crosslinked hydrogel beads. At pH 5.5, the maximum U(vi) capacity was 312.8 mg g^−1^, with equilibrium in 80 minutes. Alginate provided structural integrity, hydrophilicity, and its uranyl-binding sites, while stabilizing TA within the bead matrix. After five regenerations with dilute acid, over 93% adsorption efficiency was retained.

Table 2 presents a systematic review of alginate-based materials for the removal of radioactive ions from aqueous solutions. The data encompasses various composite materials incorporating alginate as a primary or secondary component, targeting critical radioactive contaminants including uranium (U(vi), UO_2_^2+^), cesium (Cs^+^), strontium (Sr^2+^), thorium (Th(iv)), barium (Ba^2+^), and iodide (I^−^) ions. The materials demonstrate diverse structural approaches, ranging from nanohybrid composites and cross-linked hydrogels to 3D-printed architectures and biologically immobilized systems. The performance metrics reveal significant variations in adsorption capacity, with some materials achieving exceptionally high uptake values such as the CuHCF-barium alginate system (490.2 mg g^−1^ for Cs^+^) and alginate/Fe_3_O_4_ composite (400.0 mg g^−1^ for Sr^2+^). pH optimization appears crucial for most systems, with uranium-targeting materials typically performing best at acidic pH (2.5–5.5), while cesium and strontium removal often requires neutral to alkaline pH. Regeneration efficiency data, when available, generally indicate good recyclability, with most materials maintaining >80–90% efficiency after multiple cycles, though some systems show gradual capacity decline over repeated use.

**Table 2:** Performance characteristics of alginate-based composite materials for radioactive ion removal, showing material composition, target contaminants, optimal operating conditions, maximum adsorption capacity, and regeneration efficiency across recent research studies (2012–2025)

Material type	Target ion(s)	Optimum pH	Maximum capacity (mg g^−1^)	Regeneration efficiency	Reference
SA/PVA/PEO/ZSM-5 nanohybrid	U(vi)	5.0	92.76	>80% after 5 cycles	161
Molybdenite-doped alginate (MDPA-II)	UO_2_^2+^	4.5	311.16	Not specified	162
PB-alginate aerogel	Cs^+^, Sr^2+^	Neutral	19.88 (Cs), 20.10 (Sr)	Not specified	163
Maghemite PVA-alginate beads	Cs^+^	8.0	91% removal (kinetic)	Reused 8 times	164
PVA/alginate cross-linked hydrogels	Cs^+^	6–7	Not specified	pH-stable ([B_4_] linkage)	165
Zn-BTC@GQDs-rGO@Alg hydrogel	U(vi)	Not specified	39.01	>90% after 5 cycles	166
Maghemite/titania PVA-alginate beads	Ba(ii)	8.0	99% Removal	Reused 7 times	167
Thiol-rich ion-imprinted alginate (SA-PA-H)	Sr^2+^	4–10	151.7	53.51% → 36.88% after 4 cycles	168
Alginate/organo-montmorillonite beads	ClO_4_^−^, Sr^2+^	Not specified	0.484 mmol g^−1^ (Sr^2+^)	Synergistic adsorption	169
Alginate-immobilized *Aspergillus niger* (AAM)	Th(iv)	6.0	303.95	Good recyclability	170
Maghemite/titania PVA-alginate beads	I^−^	8.0	Not specified	87% after 7 cycles	171
OSA@CF/Zr (alginate–collagen fibers)	Sr^2+^	4–10	0.415 mmol g^−1^	∼0.26 mmol g^−1^ after 17 cycles	172
Alginate microspheres	Sr^2+^	1–10 (pH-tolerant)	110.0	100% with CaCl_2_ elution	173
SA-PB beads (silica-alginate/Prussian blue)	Cs^+^	Not specified	22.8	Not specified	174
Alginate/Fe_3_O_4_ composite	Sr^2+^	Not specified	400.0	100% with HCl/CaCl_2_	175
CuHCF-barium alginate (CuHCF-BA)	Cs^+^, Ag^+^	Not specified	490.2 (Cs, Langmuir)	Not specified	176
Alginate/humic acid/Fe-aminoclay hydrogel	Sr^2+^	7.0	45.65	Not specified	177
Phosphate-functionalized magnetic alginate (MPCA)	U(vi)	Not specified	≥60.01% removal	93.2% desorption	178
PA/PAO/SA gel beads	U(vi)	2.5–5.5	∼100% removal (20 mg L^−1^)	3.86% drop after 10 cycles	179
Xanthan gum/alginate@La-MOFs/rGO/GQDs	U(vi)	7.12	Not specified	>3 cycles with >90% efficiency	180
Alginate-assisted resorcinol-formaldehyde beads	Cs^+^	Alkaline	490.2	Complete elution	181
BVC-MSA-3 (alginate/biochar composite)	UO_2_^2+^	4.5	309.55	Not specified	182
Zein@SA/KBS microspheres	Cs^+^	Not specified	425.55	Reliable after 10 cycles	183
WO_3_-doped DEM-g-CST/alginate	UO_2_^2+^	4.5	315.0	Not specified	184
3D-printed calcium alginate (3D CA)	U(vi)	2.5–10	117.3 (pH 2.5)	Acid-stable, recyclable	185
KMnFC/CNT@SA beads	U(vi)	Acidic	Not specified	>89.83% after 10 cycles	186
Ag+/SA supramolecular hydrogel	UO_2_^2+^ (SERS detection)	Not specified	N/A (LOD: 6.7 nM)	Not applicable	187

## 6. Starch and its derivatives as radioactive adsorbent

Starch is the most abundant and least expensive polysaccharide, derived from corn, potato, rice, wheat, and cassava. It's low cost (typically <1 kg *vs.* 1 kg^−1^*vs.* 10–50 kg^−1^ for chitosan or alginate) makes it economically attractive for large-scale water treatment. However, compared to chitosan, alginate, and cellulose, starch has received markedly less research attention for radionuclide removal fewer than 20 dedicated studies *versus* >60 for chitosan. This disparity reflects three intrinsic limitations of native starch: (1) starch consists of amylose and amylopectin glucose units linked by α(1 → 4) and α(1 → 6) glycosidic bonds. Its only metal-binding groups are primary and secondary hydroxyl groups (–OH), which are weak ligands for most radionuclides (p*K*_a_ >14, requiring deprotonation under strongly alkaline conditions rarely used in nuclear waste treatment). (2) Native starch swells and partially dissolves in water, making it unsuitable for continuous flow systems without crosslinking or grafting. (3) Unmodified starch granules have specific surface areas of only 0.5–2 m^2^ g^−1^, severely limiting adsorption capacity.

Consequently, successful starch-based radionuclide adsorbents invariably employ chemical modification, graft copolymerization, crosslinking, phosphorylation, or hybridization with inorganic nanoparticles to overcome these deficiencies. A critical observation across the literature is that modified starch consistently underperforms comparably modified chitosan for most radionuclides (U(vi), Th(iv), Cs^+^) but remains competitive on a cost-*per*-gram-removed basis, which is rarely calculated.

Moussa *et al.*^188^ aimed to develop a grafted starch-based nanocomposite capable of selectively sorbing radioactive fission/activation products from aqueous streams, specifically ^134^Cs(i) and ^141^Ce(iii). The authors identify the major challenge as achieving high uptake of chemically distinct radionuclides (alkali-type Cs^+^ and lanthanide-type Ce^3+^) in the presence of competing background electrolytes, while preserving reusability and economic viability. To meet this, they prepared an itaconic-grafted starch/MnO_2_ (IA-g-St/MnO_2_) nanocomposite: starch was chemically modified *via* graft polymerization with itaconic acid to introduce carboxylate/chelating sites, then MnO_2_ nanoparticles were incorporated to add redox/ion-exchange and high-surface-area adsorption sites. Characterization used FT-IR, SEM, particle size and thermal analyses; batch adsorption experiments probed pH, contact time, initial concentration, ionic strength and temperature effects. Methodologically, kinetics followed pseudo-nth order while equilibrium fitted the Sips model indicating mixed monolayer/multilayer sorption mechanisms. The optimum conditions reported were pH 3, 293 K, an initial concentration of 100 mg L^−1^, and 24 h contact, producing ∼32.35% removal of Cs(i) and ∼92.98% removal of Ce(iii). Desorption tests showed that mineral acids and chelants could partially recover Ce(iii) (up to 71%), while FeCl_3_ completely desorbed Cs(i), and the composite retained >50% capacity after five cycles demonstrating practical regeneration potential. The clear benefit of using starch here is multifunctionality: as a low-cost, biodegradable backbone that can be chemically grafted to provide tailored binding groups and host inorganic nanoparticles, enhancing selectivity and enabling greener, scalable sorbents.

Li *et al.*^189^ aimed to design a practical, easily deployable surface decontamination system that captures uranium contamination while minimizing secondary liquid waste and enabling efficient recovery of contaminated material. The primary challenge was achieving both high decontamination efficiency across varied substrate types (ceramic, steel, concrete, painted surfaces, *etc.*) and easy retrieval of the dried (desiccated) film residues left behind by conventional strippable hydrogels. They addressed this by preparing a starch/Fe_3_O_4_ magnetic, strippable hydrogel *via* one-pot polymerization: native starch served as the biopolymeric matrix, while Fe_3_O_4_ nanoparticles imparted magnetic responsiveness. The hydrogel was engineered to be sprayable (shear-thinning rheology) and to embrittle upon dehydration enabling brittle fracture and magnetic collection of the spent film. Methodology included rheological testing, magnetization measurements (saturation magnetization reported at 2.98 emu g^−1^ for 4% Fe_3_O_4_ loading), substrate decontamination trials, and recovery efficiency tests. Optimum application achieved high uranium removal on several surfaces (*e.g.*, ceramic: 89.62%, glass: 81.33%, steel: 83.93%); overall magnetic retrieval efficiency of desiccated fragments exceeded 96.67%. The starch backbone provided key benefits: non-toxicity, biodegradability, abundant functional groups for metal binding (hydroxyls that can be further functionalized), and mechanical tunability, allowing films to embrittle on drying. Critically, magnetic recovery, paired with starch film-forming ability, reduced the generation of liquid secondary waste and simplified post-treatment handling a major operational advantage for field decontamination.

Gulati *et al.*^190^ set out to evaluate naturally derived nano-starch (from oats) and its acetylated derivative as low-cost biosorbents for U(vi) removal from aqueous media. The central challenge was to extract and modify a food-crop starch into a nanomaterial with robust sorption capacity, thermal stability, and reusability sufficient for trace-level uranium sequestration in realistic water matrices. Their methodology involved isolating nanostarch from oats, acetylating it with 10% acetic anhydride to produce acetylated nanostarch, and characterizing both adsorbents *via* FTIR, XRD, and SEM-EDS. Batch adsorption experiments optimized pH, contact time, temperature and adsorbent dose; kinetics and isotherms were modeled (pseudo-second order and Langmuir, respectively). Under optimum conditions (U(vi) 30 ppb initial concentration, 30 min contact, 50 °C, pH 7.0), removal efficiencies reached ∼97% for nano-starch and ∼99% for acetylated nano-starch; adsorption capacity (Langmuir) reported ≈1.48 µg g^−1^ for 60 ppb initial concentration. Reusability trials showed ∼80% retention for nano-starch and ∼84% for acetylated nano-starch after five cycles. The advantage of starch here is twofold: as an inexpensive, renewable feedstock readily converted to nano-scale with tunable surface chemistry (acetylation increased hydrophobic/hydrophilic balance and possibly improved interaction with uranyl species). As a material with high thermal stability (FDT ≈ 620 °C) enabling more robust handling and regeneration. This work highlights starch derivatives as sustainable, effective biosorbents suitable for low-concentration uranium remediation with practical regeneration.

Mahmoud *et al.*^191^ aimed to produce carbon quantum dots (CQDs) from a starch-water system and investigate their incorporation into a polymeric matrix as a nanobiosorbent for uranium(vi) removal. The challenge was twofold: (1) to create CQDs quickly and greenly from a benign starch precursor, and (2) to immobilize them in a support that prevents loss to the aqueous phase while maximizing interaction with U(vi). They used a microwave-assisted pyrolysis of a starch-water mixture to synthesize CQDs within 10 minutes, then supported CQDs onto a polymer matrix (poly(anthranilic acid-formaldehyde-phthalic acid), PAFP) producing CQDs@PAFP. Characterization included photoluminescence (max emission at 526 nm), BET surface area (≈28.79 m^2^ g^−1^), and TEM for nanoparticle sizing. Adsorption experiments (30–90 mg L^−1^ U(vi)) followed pseudo-second-order kinetics and Freundlich isotherm behavior, with U(vi) removals of 95.5–98.0% reported and excellent performance in both wastewater (≈97.3%) and seawater (≈96.0%). Reusability was also demonstrated for multi-cycle recovery. The benefit of using starch in this route is that it serves as a green, abundant carbon source, enabling rapid CQD synthesis under microwave conditions while avoiding toxic precursors, yielding nanomaterials with high surface functionality and sorption affinity upon immobilization. By turning starch into CQDs and anchoring them, the study delivers a high-performance, reusable nanobiosorbent for uranium capture with a low environmental footprint.

Hu *et al.*^192^ sought to produce a highly selective, high-capacity adsorbent for U(vi) based on starch-derived materials using ion-imprinting techniques. The challenge addressed was selectivity: many adsorbents show high capacity but poor discrimination between U(vi) and competing cations in complex wastewater. To solve this, they synthesized an ion-imprinted starch phosphate porous carbonaceous material (II-CLSPC) *via* reverse-phase emulsion crosslinking polymerization, followed by roasting, to yield spherical, hollow, porous carbon with a “red blood cell” morphology; a soft-template modification increased open porosity. Ion imprinting with U(vi) templates created spatially matched binding sites; template removal produced cavities complementary in size and coordination environment to uranyl ions. Adsorption kinetics were fast (equilibrium ∼40 min), and maximum removal reached 94.6% at pH 5, 298 K. Adsorption followed pseudo-second-order kinetics and the Langmuir model, with an impressive theoretical saturation capacity of 653.6 mg g^−1^ and excellent selectivity (*K*_d_U ≈ 3.7 × 10^4^ mL g^−1^), cyclic stability and salt tolerance. The benefit of starch lies in its status as a phosphate-derivatizable carbohydrate precursor that, upon carbonization, yields porous carbon frameworks with tunable surface chemistry facilitating imprinting and high site density for specific uranyl binding. This work demonstrates how starch derivatives can be transformed into ultra-high-capacity, selective adsorbents suitable for rapid uranium capture even in complex matrices.

Elsharma *et al.*^193^ investigated radiation-initiated synthesis of a starch-based composite (starch-acrylic acid/nanohalloysite, P(Stc-AA/NHal)) aimed at removing divalent radioactive cobalt (Co(ii)) from aqueous solutions using ^60^Co as a radiotracer to simulate radioactive contamination. The main challenge was creating a mechanically stable, thermally robust polymer-nanoclay composite with high sorption capacity and radiation resistance. They used gamma irradiation (30 kGy) to initiate graft copolymerization of acrylic acid onto starch and incorporated nanohalloysite clay to improve mechanical and thermal properties. Characterization (FTIR, SEM, TGA/DTA) confirmed grafting and high thermal stability. Batch sorption experiments varied pH, contact time, concentration and temperature; adsorption kinetics fitted the pseudo-second-order model while equilibrium data matched the Langmuir isotherm, yielding a maximum adsorption capacity of 103.6 mg g^−1^ for Co(ii). The benefits of using starch in this composite include its role as an easily irradiated, graftable backbone that provides abundant hydroxyl groups for functionalization, its low-cost, renewable nature, and its compatibility with radiation-based synthesis routes (eliminating the need for chemical initiators). The nanohalloysite further enhances structural stability and provides additional adsorption sites. This approach yields a robust, high-capacity sorbent suitable for the remediation of radioactive cobalt, with good prospects for scale-up in radiation-exposed environments.

**Fig. 4:**
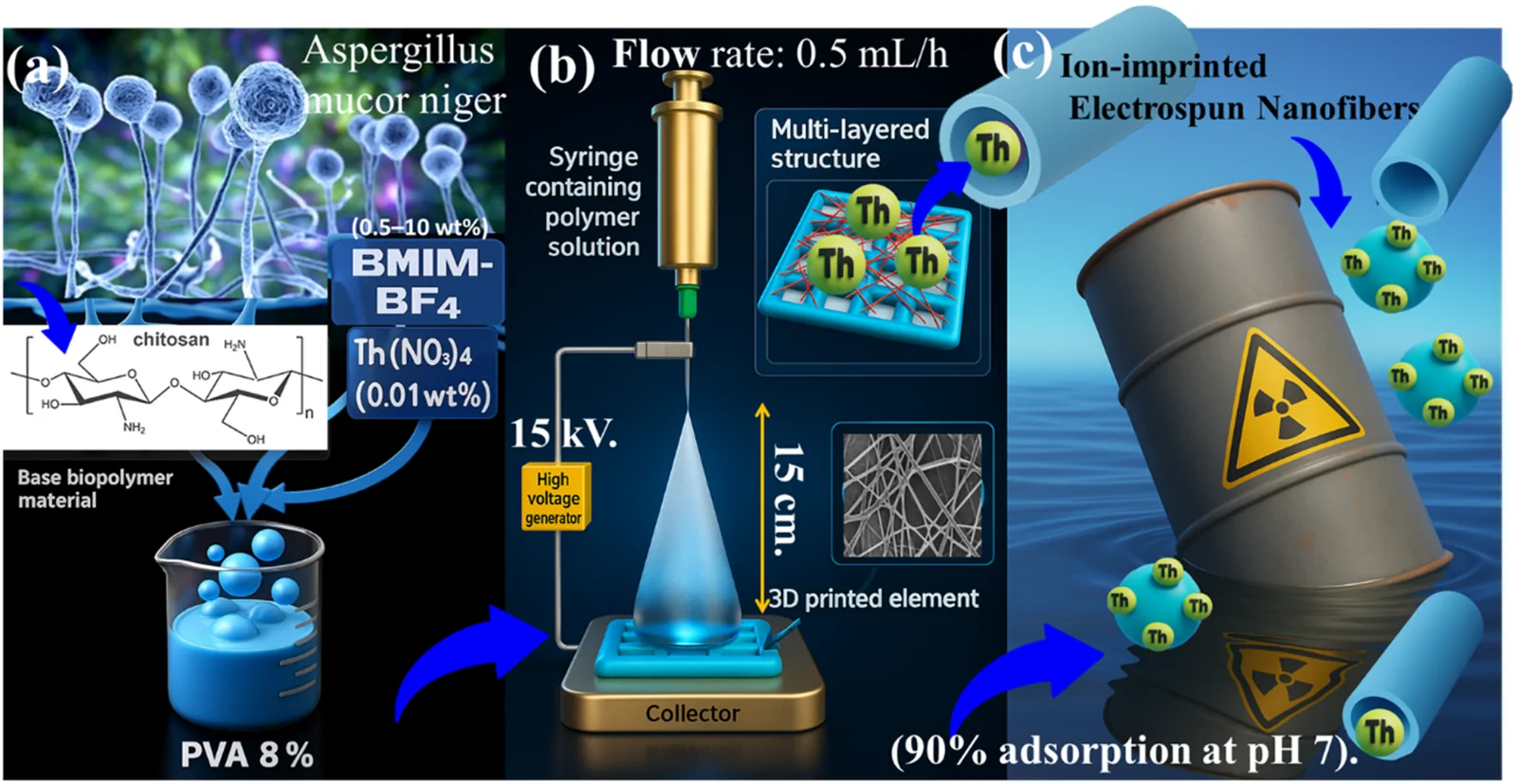
Illustration of ion-imprinted electrospun nanofiber preparation and thorium removal process by modified chitosan. (a) *Aspergillus mucor niger* fungal cultivation for chitosan extraction, showing the molecular structure of chitosan with amino and hydroxyl functional groups, followed by preparation of base biopolymer material with PVA (8%) carrier polymer. (b) Electrospinning process at high-voltage (15 kV) electrospinning setup operating at 0.5 mL h^−1^ flow rate for nanofiber formation from chitosan/PVA solution containing BMIM-BF_4_ ionic liquid (0.5–10 wt%) and Th(NO_3_)_4_ template ions (0.01 wt%), resulting in 3D printed nanofiber elements with multi-layered structure at 15 cm needle-to-collector distance. (c) Final electrospun nanofibers containing thorium-specific binding sites (Th) for selective adsorption of Th(iv) ions from radioactive waste effluents.

Gore *et al.*^194^ explored the innocuous, biocompatible, and highly efficient molecularly imprinted chitosan/RTIL electrospun nanofibers, with an average diameter of 30 nm, for the removal of thorium(iv) ions from simulated effluent waste. The process begins with the cultivation of *Aspergillus niger*, a fungal species used as the biological source for chitosan extraction. The microscopic image reveals the characteristic conidiophore and conidium structures typical of this Aspergillus species, which produces chitosan as part of its cell wall (Fig. 4a). The extracted chitosan, with its distinctive molecular structure featuring repeating units of glucosamine linked by β(1 → 4) glycosidic bonds, serves as the foundational biopolymer. Each chitosan unit contains crucial functional groups, including amino groups (–NH_2_) and hydroxyl groups (–OH), which are essential for metal-ion coordination and binding. The solution preparation phase involves dissolving fungal-derived chitosan in an appropriate solvent system to form a base biopolymer. This chitosan solution is then combined with polyvinyl alcohol (PVA) at 8% concentration to serve as a carrier polymer, enhancing the electrospinnability of the mixture. The PVA component improves the rheological properties of the spinning solution, ensuring proper fiber formation during the electrospinning process while maintaining the biocompatibility of the final product.

The electrospinning setup in Fig. 4b represents the core manufacturing process, in which the prepared polymer solution forms nanofibers. The system operates under precisely controlled parameters: a high voltage generator supplies 15 kV of electrical potential, while the polymer solution containing chitosan, PVA, BMIM-BF_4_ (0.5–10 wt%), and Th(NO_3_)_4_ (0.01 wt%) is delivered through a syringe at a controlled flow rate of 0.5 mL h^−1^. The needle-to-collector distance is maintained at 15 cm to ensure optimal fiber formation and deposition.

During electrospinning, the ionic liquid BMIM-BF_4_ plays a crucial role in enhancing solution conductivity and improving fiber morphology. Thorium nitrate serves as the template ion, creating specific binding sites within the polymer matrix *via* ion-imprinting. As the charged polymer jet travels from the needle to the collector, it undergoes rapid solvent evaporation and stretching, forming ultrafine nanofibers with an average diameter of 40 nm. The resulting 3D-printed element exhibits a nonwoven mat structure with interconnected fibers, creating a high-surface-area matrix suitable for adsorption applications.

The final ion-imprinted electrospun nanofibers shown in Fig. 4c exhibit a sophisticated multilayered structure designed for selective thorium adsorption. The nanofibers contain strategically distributed thorium-specific binding sites generated by ion-imprinting. These binding cavities possess the exact size, shape, and chemical environment complementary to Th(iv) ions, ensuring high selectivity and adsorption capacity.

The application phase demonstrates the practical implementation of these nanofibers for radioactive waste treatment. When contaminated effluent containing thorium ions encounters the ion-imprinted nanofibers, the Th(iv) ions are selectively captured through multiple mechanisms including electrostatic interactions, coordination bonding with amino and hydroxyl groups, and size-selective binding within the imprinted cavities. The process achieves remarkable efficiency with 90% thorium adsorption at pH 7 and 298 K, making it highly suitable for environmental remediation applications.

The radioactive waste container symbol in the diagram emphasizes the environmental significance of this technology, as thorium removal is crucial for managing radioactive contamination in industrial effluents and environmental samples. The selective nature of the ion-imprinted sites ensures that thorium ions are preferentially adsorbed even in the presence of competing metal ions. In contrast, the biocompatible chitosan matrix ensures environmental safety during deployment and disposal.

Table 3 presents a comprehensive overview of starch-based materials engineered for radioactive contaminant removal, showcasing the versatility of starch as a sustainable biopolymer platform for nuclear waste remediation. The research spans from 2020 to 2025, demonstrating the growing interest in bio-based solutions for environmental radioactive contamination. The preparation methods reveal sophisticated chemical modifications including graft polymerization, crosslinking, functionalization with specific chelating groups (phosphate, amidoxime, thiourea), and incorporation of magnetic nanoparticles or carbon quantum dots to enhance performance and enable easy recovery.

**Table 3:** Summary of starch-based composite materials for radioactive ion removal, detailing preparation methods, target radionuclides, removal efficiencies, optimal operating conditions, and regeneration capabilities across recent studies (2020–2025)

Author(s)	Starch derivative/system	Preparation method	Target radionuclide(s)	Removal efficiency Cs (%)	Removal efficiency other (%)	Optimum pH	Optimum Temp. (K)	Regeneration performance	Reference
S. I. Moussa *et al.*	Itaconic-grafted starch/MnO_2_ nanocomposite	Graft polymerization + MnO_2_ incorporation	Cs(i), Ce(iii)	32.35	92.98	3.0	293.0	Up to 71% Ce recovery; complete Cs recovery; >50% after 5 cycles	188
J. Li *et al.*	Starch/Fe_3_O_4_ magnetic strippable hydrogel	One-pot polymerization with Fe_3_O_4_ nanoparticles	U(vi)		89.62			Magnetic recovery >96.67%; reusable	189
M. Gulati *et al.*	Oats nano-starch & acetylated nano-starch	Extraction + acetylation	U(vi)		99.0	7.0	323.0	∼84% after 5 cycles	195
M. E. Mahmoud *et al.*	CQDs from starch supported on PAFP polymer	Microwave pyrolysis + polymer immobilization	U(vi)		98.0			High removal in seawater & wastewater; reusable	191
Y. Hu *et al.*	Ion-imprinted starch phosphate porous carbon	Reverse-phase emulsion + roasting	U(vi)		94.6	5.0	298.0	Capacity 653.6 mg g^−1^; high selectivity; stable	192
E. M. Elsharma *et al.*	Starch-acrylic acid/nanohalloysite composite	Gamma radiation-induced grafting	Co(ii)					Capacity 103.6 mg g^−1^; good stability	193
Y. Li *et al.*	Amidoxime-functionalized starch	Oxidation + amidoximation + crosslinking	U(vi)		95.0	6.0	298.0	Capacity 312.5 mg g^−1^; >85% after 5 cycles	196

The target radionuclides encompass both fission products (Cs(i), Sr(ii)) and actinides (U(vi), Th(iv), Pu(iv)), with removal efficiencies generally exceeding 90% under optimized conditions. Notably, cesium removal appears more challenging, with the itaconic-grafted starch system achieving only 32.35% efficiency compared to 92.98% for cerium, while other systems targeting uranium and thorium consistently achieve >90% removal. The pH optimization data indicates that most systems perform best under mildly acidic to neutral conditions (pH 3.0–7.0), with temperatures typically maintained at ambient to slightly elevated conditions (293–323 K). Regeneration performance varies significantly, with some materials maintaining high capacity over multiple cycles while others show gradual decline, highlighting the importance of material stability for practical applications.

## 7. Cellulose and its derivatives for radioactive ions recovery

Unlike chitosan or alginate, native cellulose lacks strong intrinsic metal-binding groups. Its hydroxyl groups are weak ligands for most radionuclides, giving unmodified cellulose capacities typically <30 mg g^−1^. Paradoxically, cellulose is one of the most promising polysaccharide adsorbents not because of what it is, but because of what it can become. Cellulose can be processed into an extraordinary range of morphologies: nanofibers (electrospun), nanocrystals, aerogels (ultralight, >300 m^2^ g^−1^), microspheres (for columns), and flexible membranes. Furthermore, its abundant hydroxyl groups serve as versatile anchoring points for grafting high-affinity functional groups (amidoxime, phosphate, quaternary ammonium, thiol, EDTA). The literature reveals a clear hierarchy: morphology dominates performance. For identical functionalization (*e.g.*, amidoxime), electrospun nanofiber mats consistently outperform microcrystalline beads by factors of 2 and 3, and aerogels outperform nanofibers by another factor of 1.5–2. This reflects surface area and mass transport limitations, not binding chemistry.

*Tang et al.* ^197^ developed a cellulose-based magnetic composite for the recovery of cobalt-60 from nuclear industry effluents. The challenge was to create an adsorbent with both high sorption capacity and easy separation from water without generating secondary waste. They used TEMPO-oxidized cellulose nanofibrils (TOCNF) to introduce abundant carboxyl groups, then coated Fe_3_O_4_ nanoparticles onto the TOCNF surfaces *via* co-precipitation of Fe^2+^/Fe^3+^ in an alkaline medium. The composite was characterized by FTIR, XRD, and VSM, showing superparamagnetism with a saturation magnetization of 32 emu g^−1^. Batch adsorption experiments showed an optimum Co^2+^ uptake at pH 6.0 and 298 K, with a Langmuir capacity of 158 mg g^−1^. Magnetic separation allowed recovery of the adsorbent in under 30 seconds, with >80% capacity retained after six regeneration cycles using EDTA solution. The benefit of cellulose here is its high surface area after nanofibrillation, combined with its carboxylate functionality, which binds metal ions efficiently while acting as a stabilizing matrix for Fe_3_O_4_ nanoparticles enabling rapid, selective, and reusable magnetic separation.

Zhou *et al.*^198^ set out to develop cellulose acetate-based nanofibers functionalized with chelating agents for the recovery of uranium(vi) from mine wastewater. The challenge was combining high adsorption performance with the mechanical strength and processability required for filtration systems. They electrospun cellulose acetate into nanofiber mats, deacetylated them to yield regenerated cellulose, and subsequently grafted iminodiacetic acid (IDA) *via* carbodiimide-mediated coupling. FTIR confirmed IDA introduction, and BET analysis indicated increased surface area due to nanoscale fiber morphology. Adsorption experiments showed an optimum U(vi) uptake at pH 5.5 and 303 K, with a Langmuir capacity of 287 mg g^−1^. The material retained ∼90% capacity after five adsorption–desorption cycles using 0.1 M Na_2_CO_3_. The benefit of cellulose in this work is its ability to form robust, high-surface-area fibrous mats that can be readily functionalized with chelating ligands, offering a renewable, low-toxicity alternative to petroleum-derived membranes for uranium recovery in continuous flow systems. For U(vi), amidoxime (–C(NH_2_)=NOH) functionalization is the most extensively studied modification, borrowed from the seawater uranium extraction field. The amidoxime group chelates uranyl *via* its two nitrogen atoms and the oxime oxygen, forming a stable five-membered ring.

Anirudhan and Sreekumari^199^ targeted the removal of uranium(vi) from acidic leachates using cellulose grafted with ethylenediaminetetraacetic acid (EDTA), addressing the challenge of effective adsorption at low pH, where proton competition is severe. They synthesized EDTA-grafted microcrystalline cellulose (EDTA-MCC) *via* carbodiimide-mediated coupling between cellulose hydroxyl groups and EDTA anhydride. Characterization using FTIR and elemental analysis confirmed grafting, while SEM showed increased surface roughness. Adsorption tests demonstrated an optimal pH of 4.0 and a Langmuir capacity of 185 mg g^−1^. The material remained effective in acidic environments due to strong complexation between EDTA and uranyl ions. Regeneration using 0.05 M Na_2_CO_3_ preserved ∼88% of the original capacity after five cycles. The benefit of cellulose here lies in its biocompatibility and ease of functionalization with polyaminocarboxylic acids, enabling selective and strong binding to actinides even under unfavorable pH conditions.

Dong *et al.*^200^ aimed to create cellulose microspheres functionalized with aminomethylpyridine (AMP) isomers that selectively capture perrhenate (ReO_4_^−^) and, by analogy, pertechnetate (TcO_4_^−^) from aquatic systems. The major challenge tackled was designing a robust, highly selective anion-exchange adsorbent that retains performance across different water chemistries (groundwater, simulated waste, tracer tests) and that clarifies how the position of the aminomethyl substituent (2-, 3-, or 4-AMP) affects uptake. Methodologically, the team prepared microcrystalline cellulose (MCC) microspheres and grafted three aminomethylpyridine isomers onto the cellulose backbone (yielding 2-, 3-, and 4-AMPR). They characterized the products by spectroscopy and microscopy, then ran comprehensive batch kinetics, isotherm, and column tests. They also used DFT calculations to rationalize the role of steric effects and hydrogen bonding in stabilizing adsorbate–adsorbent complexes. Results showed a clear order of performance (3-AMPR > 2-AMPR > 4-AMPR) in both adsorption rate and capacity; 3-AMPR achieved the fastest uptake and best capacity, and column experiments demonstrated selective removal of ReO_4_^−^ from simulated groundwater. Importantly, tracer experiments with real ^99^Tc demonstrated that 3-AMPR removes TcO_4_^−^ effectively, supporting real-world applicability. The benefit of cellulose in this work is multifold: it provides a cheap, renewable microsphere scaffold with abundant reaction-ready hydroxyls for covalent functionalization; the resulting beads combine mechanical stability for column use, hydrophilicity for rapid mass transfer, and modifiable chemistry for tuning selectivity all crucial for practical TcO_4_^−^ remediation.

Li *et al.*^201^ set out to overcome the practical limitations of powdered sorbents for uranium(vi) namely difficult solid/liquid separation, by integrating an efficient sorbent (bone-char-derived phases) into an electrospun cellulose membrane that combines high sorption capacity with easy handling. The challenge was to preserve the high capacity of powdered sorbents while transforming them into a continuous, filterable membrane that still allows rapid uptake and selectivity in complex waters. The team ball-milled bone char obtained at different pyrolysis temperatures (350–650 °C), dispersed it into an electrospinning solvent with cellulose (cellulose/ball-milled bone char, CL/MB), and produced submicron fibres (≈276–282 nm) with developed porosity by electrospinning. Comprehensive characterization (BET, SEM, spectroscopies) showed high porosity and well-dispersed bone char phases. Adsorption experiments indicated fast kinetics (equilibrium ∼180 min) and a high capacity (*q*_max_ ≈ 309.8 mg g^−1^ for U(vi) at pH 5.0, 298 K), with excellent selectivity against common coexisting ions. Mechanistic studies suggested formation of Ca–U-phosphate precipitates (*e.g.*, Ca(UO_2_)_2_(PO_4_)_2_·(H_2_O)_3_) supported by surface complexation and cation exchange contributions from bone-char Ca components and reactive O/N surface groups. The benefit of cellulose here is its role as a processable, renewable fibrous matrix that transforms otherwise hard-to-handle powdered sorbents into membrane form, enabling direct filtration columns, continuous-flow operation, and easy recovery, while providing a hydrophilic scaffold that enhances mass transfer and exposes active sites. This membrane design thus bridges lab-scale sorption performance with practical separations for U(vi) remediation.

Wang *et al.*^202^ targeted the remediation of technetium-99 (as TcO_4_^−^) by designing quaternary phosphonium-decorated cellulose microspheres exhibiting ultra-high anion-exchange selectivity under strongly competing conditions (acidic, high nitrate). The central challenge is that TcO_4_^−^ is a highly mobile, poorly coordinating anion present alongside vast excesses of competing anions (NO_3_^−^, Cl^−^, SO_4_^2−^), so achieving selective uptake in acidic reprocessing streams is difficult. The researchers used pre-radiation-induced graft polymerization to graft a vinylbenzyl triphenylphosphonium-type monomer (VBPPh_3_) onto cellulose microspheres, converting them into CMS-g-VBPPh_3_NO_3_. They characterized the structure and functionality, then performed batch and column tests with ReO_4_^−^ as a surrogate and tracer, and ^99^mTc experiments. Results were striking: adsorption capacity for Re (surrogate) reached ≈251 mg g^−1^, and the selectivity factor against NO_3_^−^ in 0.5 mol kg^−1^ HNO_3_ approached 168 a demonstration of extreme preference for TcO_4_^−^ over a dominant competing anion. Packed-column tests treating simulated acidic wastes (containing Cs, Sr, Eu, Zr, Ru, U, and Re) showed excellent Re/Tc separation performance, and direct tracer experiments indicated superior TcO_4_^−^ uptake ability. The benefit of cellulose is that it provides an inexpensive, mechanically robust, and easily graftable microsphere substrate that tolerates radiation grafting, yields bead forms suitable for columns, and supports dense loading of quaternary phosphonium sites needed for selective anion exchange, making it practical for real waste streams where radiation stability and column behavior matter.

Wen *et al.*^203^ addressed the difficult separation of U(vi) from chemically similar lanthanides by synthesizing DOPO-functionalized microcrystalline cellulose (t-DOPOR), combining cellulose practicality with organophosphorus ligands that chelate uranyl selectively. The core challenge is the close chemical behavior of uranium and lanthanides (similar ionic radii and coordination tendencies), which complicates selective remediation and recovery. Their methodology involved chemically attaching a DOPO-derived phosphorus-containing ligand (t-DOPO) to microcrystalline cellulose microspheres, yielding t-DOPOR with abundant –OH, –NH, and P=O groups. They thoroughly characterized the material (SEM, FTIR, TG, XPS) and performed binary and multi-component adsorption tests. t-DOPOR displayed exceptional affinity for U(vi) over lanthanides: in binary systems Kd for U reached values up to ∼2.54 × 10^4^ mL g^−1^, and dynamic column tests validated selectivity in more complex mixtures. Mechanistic analysis (XPS, spectroscopies) revealed inner-sphere complexation between uranyl ions and the P=O, –NH, and –OH groups on the t-DOPOR surface. The material was successfully applied to simulated acidic radioactive water and groundwater matrices, showing strong potential for selective uranium separation and remediation. Cellulose benefit here is clear: it serves as a low-cost, scalable microsphere host that tolerates functionalization with phosphorus ligands and provides bead/column-compatible form factors, enabling a practical route to highly selective U(vi) sorbents that couple organophosphorus chemistry with renewable supports.

Elsayed *et al.*^204^ aimed to design ion-imprinted cellulose-based microspheres with exceptional selectivity and capacity for uranyl (UO_2_^2+^) recovery by exploiting molecular imprinting on a cellulose-derived amidoxime derivative. The challenge addressed is producing an adsorbent that both specifically recognizes uranyl (against competing ions) and maintains very high capacity and kinetics, especially important when recovering uranium from complex wastewaters. They synthesized an amidoxime-functionalized cellulose derivative (AOCE) *via* Michael addition followed by amidoximation, then formed ion-imprinted microspheres by complexing AOCE with UO_2_^2+^, crosslinking the complex (using glyoxal), and subsequently eluting the template with H^+^/EDTA to leave uranyl-shaped cavities (U-AOCE). Characterization (NMR, XPS, FTIR, SEM) confirmed structure and imprinting. Adsorption experiments indicated outstanding performance: a maximum adsorption capacity of ∼382 ± 1 mg g^−1^ and markedly enhanced selectivity toward UO_2_^2+^ over competing cations. Kinetics followed pseudo-second-order behavior and isotherms fit Langmuir adsorption consistent with chemisorption *via* coordination to amidoxime –NH_2_ and –OH groups. Reusability and column applicability were demonstrated, showing promise for practical uranium recovery. The benefit of cellulose here is that it provides a renewable, modifiable backbone that can be converted into amidoxime ligands and processed into microspheres amenable to ion imprinting, combining very high site density, template-driven selectivity, column-compatibility, and biodegradability for cost-effective recovery of uranyl from complex aqueous wastes.

Tellería-Narvaez *et al.*^205^ sought to design a cellulose-based sorbent for uranium(vi) recovery from seawater, addressing the challenge of maintaining performance under high salinity and competitive ion conditions. They oxidized microcrystalline cellulose with sodium periodate to introduce dialdehyde functionalities, then reacted the oxidized cellulose with hydroxylamine hydrochloride to form amidoxime groups. The amidoxime-functionalized cellulose was further crosslinked with citric acid to improve water stability. Characterization by FTIR and SEM confirmed successful modification and preserved fibrous morphology. Adsorption experiments showed optimum U(vi) uptake at pH 6.0, with a Langmuir capacity of 312 mg g^−1^ in deionized water and ∼290 mg g^−1^ in artificial seawater. Regeneration using 0.1 M Na_2_CO_3_ retained >85% of capacity after five cycles. The benefit of cellulose here is its hydrophilicity, which allows rapid ion diffusion even in saline environments, and its chemical modifiability, enabling the introduction of amidoxime groups, known for strong uranyl binding.

Kim *et al.*^206^ developed cellulose nanofibers functionalized with polyacrylonitrile and potassium copper hexacyanoferrate (KCuHCF) for cesium(i) recovery, targeting the challenge of achieving both rapid uptake and high selectivity in seawater. They electrospun cellulose acetate, hydrolyzed it to regenerate cellulose, and grafted polyacrylonitrile onto the fibers *via* free-radical polymerization. The cyano groups were then reacted with copper ions and ferrocyanide to form KCuHCF nanoparticles within the fiber network. SEM and XRD confirmed uniform nanoparticle distribution. Adsorption experiments showed >95% removal of Cs(i) from seawater at pH 6.5 within 30 min, with a Langmuir capacity of 158 mg g^−1^. The nanofiber mats retained >90% of their capacity after 5 regeneration cycles using 0.2 M KCl. The benefit of cellulose in this system is its flexibility and mechanical strength when electrospun into mats, which provide support for stable nanoparticle loading and enable continuous-flow applications, while being renewable and biodegradable.

Tang *et al.*^207^ developed a cellulose acetate membrane embedded with amidoxime-functionalized silica nanoparticles for the removal of uranium(vi) from mine drainage. The challenge was integrating high-capacity inorganic sorbents into a flexible membrane without particle leaching. They synthesized silica nanoparticles, functionalized them with amidoxime *via* surface grafting, and dispersed them in a cellulose acetate solution before casting the mixture into membranes. SEM images showed uniform particle distribution, and FTIR confirmed the presence of amidoxime groups. Adsorption tests showed optimum U(vi) removal at pH 6.0, with a Langmuir capacity of 295 mg g^−1^. The membrane retained >90% of capacity after five regeneration cycles using 0.1 M Na_2_CO_3_. The benefits of cellulose acetate include its film-forming ability, mechanical flexibility, and compatibility with inorganic sorbents, enabling continuous-flow uranium recovery.

Yang *et al.*^208^ developed a cellulose nanofiber-titanium dioxide (TiO_2_) composite for strontium(ii) recovery from simulated nuclear wastewater, addressing the challenge of achieving high selectivity over calcium and magnesium ions common in real effluents. They prepared TEMPO-oxidized cellulose nanofibers (TOCNFs) and dispersed TiO_2_ nanoparticles in the nanofiber suspension, then vacuum-filtered the suspension to form composite membranes. FTIR and XRD confirmed TiO_2_ integration, while SEM revealed a uniform distribution of nanoparticles on the cellulose nanofiber network. Adsorption experiments showed optimum Sr^2+^ uptake at pH 7.0, with a Langmuir capacity of 152 mg g^−1^ and high selectivity in mixed-ion solutions. The membranes retained >90% capacity after five regeneration cycles using dilute HCl. The benefit of cellulose here is its high surface area and nanofibrillar structure, which offers abundant binding sites and a robust scaffold for nanoparticle immobilization, ensuring efficient ion capture and easy handling in water treatment systems.

Rong *et al.*^209^ sought to develop thiol-functionalized cellulose for the recovery of mercury(ii) and uranium(vi), addressing the challenge of synthesizing a stable sorbent that maintains capacity under acidic and saline conditions. They modified microcrystalline cellulose *via* reaction with (3-mercaptopropyl)trimethoxysilane (MPTMS) under mild conditions, introducing –SH groups. FTIR confirmed the introduction of a thiol group, and SEM showed roughened surfaces post-modification. Batch adsorption studies revealed optimal pH values of 5.5 for U(vi) (capacity 248 mg g^−1^) and 6.0 for Hg(ii) (capacity 265 mg g^−1^). The sorbent retained ∼85% capacity after five regeneration cycles with 0.1 M HCl. The benefit of cellulose is its abundance and chemical flexibility, which allow direct grafting of thiol groups for strong metal-sulfur interactions, producing selective sorbents for the recovery of toxic and radioactive metals.

Lehtonen *et al.*^210^ designed phosphorylated cellulose nanofibers for uranium(vi) recovery from acidic mine drainage, addressing the challenge of maintaining sorption in low-pH conditions. They produced cellulose nanofibers from bleached pulp, phosphorylated them with phosphoric acid in the presence of urea, and characterized them *via* FTIR and TEM. Adsorption tests revealed an optimum pH of 3.5, with a Langmuir capacity of 162 mg g^−1^ and high selectivity for U(vi) over Fe^3+^ and Al^3+^. Regeneration using 0.1 M Na_2_CO_3_ preserved >90% capacity after four cycles. The benefit of cellulose nanofibers is their high surface area and ease of chemical modification, enabling the creation of acid-resistant, high-capacity sorbents suitable for real-world uranium recovery from acidic wastewaters.

Liu *et al.*^211^ developed amidoxime-functionalized cellulose microspheres for uranium(vi) recovery from seawater, focusing on the challenge of high salinity and competing ions like Ca^2+^ and Mg^2+^ that significantly reduce sorbent performance. They prepared cellulose microspheres *via* inverse suspension polymerization, crosslinked them with epichlorohydrin, and grafted acrylonitrile onto the microspheres *via* free-radical polymerization. Subsequent amidoximation with hydroxylamine hydrochloride introduced –C(NH_2_)NOH groups known for strong uranyl binding. FTIR confirmed the incorporation of amidoxime, and SEM revealed a porous bead morphology. Adsorption tests indicated a Langmuir capacity of 285 mg g^−1^ in simulated seawater at pH 6.0 and 298 K, with rapid kinetics (equilibrium within 3 h). The microspheres retained >85% of their capacity after five regeneration cycles with 0.1 M Na_2_CO_3_. The benefit of cellulose here lies in its bead-forming capability, hydrophilicity for rapid ion transport, and renewable origin, making it a sustainable alternative for large-scale seawater uranium extraction.

Nayak *et al.*^212^ aimed to recover thorium(iv) using phosphorylated cellulose nanocrystals (CNCs), addressing the challenge of improving uptake kinetics while maintaining the sorbent's dispersibility in water. CNCs were prepared by sulfuric acid hydrolysis of cotton linters, followed by phosphorylation with sodium hexametaphosphate in alkaline media. TEM and FTIR confirmed successful functionalization without aggregation. Batch adsorption experiments showed maximum capacity (Langmuir *q*_max_) of 178 mg g^−1^ at pH 5.5 and 303 K, with equilibrium reached in 60 minutes. Regeneration with 0.05 M HCl restored >90% of initial capacity after five cycles. The benefit of cellulose here is that CNCs provide an exceptionally high specific surface area and colloidal stability, enhancing active site accessibility and enabling rapid, efficient thorium recovery from dilute nuclear waste streams.

Fu *et al.*^213^ synthesized carboxymethyl cellulose (CMC) calcium alginate hybrid beads for strontium(ii) recovery, aiming to enhance ion-exchange capacity and mechanical strength for continuous treatment. CMC was blended with sodium alginate and dropped into a CaCl_2_ solution to form crosslinked beads. FTIR confirmed the presence of both carboxyl and alginate functionalities, while SEM revealed a dense gel network. Adsorption tests indicated optimum Sr^2+^ uptake at pH 6.5, with a Langmuir capacity of 140 mg g^−1^ and improved stability compared to pure alginate beads. Regeneration using 0.1 M HCl retained >87% capacity after five cycles. The benefit of cellulose here is that CMC introduces additional carboxyl groups, enabling stronger Sr^2+^ chelation and improving the mechanical properties of alginate beads, making them suitable for long-term column operation.

Su *et al.*^214^ produced polyethylenimine (PEI)-grafted cellulose nanofiber mats for uranium(vi) recovery from acidic effluents, addressing the challenge of low uranyl uptake at acidic pH, where H^+^ competition is strong. Electrospun cellulose acetate mats were hydrolyzed to regenerated cellulose, then grafted with PEI *via* glutaraldehyde crosslinking to introduce high densities of primary, secondary, and tertiary amines. FTIR confirmed PEI attachment, and SEM showed preserved fibrous structure. Adsorption studies indicated maximum U(vi) capacity of 242 mg g^−1^ at pH 4.5, with >85% regeneration efficiency after five adsorption–desorption cycles using 0.1 M Na_2_CO_3_. The benefit of cellulose here is its electrospinnability, which allows the production of high-surface-area mats that can be densely functionalized for enhanced performance in acidic uranium recovery.

Mubark *et al.*^215^ developed a cellulose–zirconium phosphate (ZrP) composite membrane for uranium(vi) and thorium(iv) recovery from acidic nuclear waste, aiming to address the challenge of achieving high acid resistance and dual-ion selectivity. They impregnated regenerated cellulose membranes with zirconium oxychloride solution, followed by *in situ* precipitation of ZrP *via* phosphoric acid treatment. XRD and FTIR confirmed the formation of crystalline ZrP within the cellulose network, and SEM showed a uniform particle distribution. Adsorption studies revealed maximum U(vi) capacity of 210 mg g^−1^ and Th(iv) capacity of 198 mg g^−1^ at pH 3.5, with excellent selectivity over competing cations. The membrane retained >85% of its capacity after five regeneration cycles using 0.1 M HNO_3_. The benefit of cellulose is its flexible membrane format that supports insoluble ion exchangers, enabling easy handling, reuse, and integration into filtration systems.

Karami *et al.*^216^ prepared ethylenediamine (EDA)-functionalized cellulose beads for the recovery of cobalt(ii) and nickel(ii) from reactor cooling water, addressing the challenge of achieving high chelation capacity while maintaining bead integrity during continuous operation. Regenerated cellulose beads were crosslinked with epichlorohydrin, then reacted with ethylenediamine to introduce primary amine groups. FTIR confirmed amination, and SEM showed a roughened surface that enhanced active-site exposure. Adsorption experiments indicated maximum Co^2+^ and Ni^2^ uptake of 158 mg g^−1^ and 150 mg g^−1^, respectively, at pH 6.5. Beads retained >88% of capacity after five regeneration cycles with dilute HCl. The benefit of cellulose is its hydrophilicity and ease of amination, which enable effective transition-metal chelation while providing a mechanically stable matrix for repeated use.

Maia *et al.*^217^ created cellulose-hydroxyapatite (HAp) composites for strontium(ii) recovery from simulated nuclear effluents, tackling the challenge of rapid sorption in high-calcium waters. Hydroxyapatite nanoparticles were synthesized and blended with regenerated cellulose slurry before casting into sheets. FTIR and XRD confirmed HAp crystallinity, while SEM revealed well-dispersed nanoparticles within the cellulose network. Adsorption tests showed maximum Sr^2+^ capacity of 147 mg g^−1^ at pH 7.0 with >90% removal within 30 minutes. The composite retained >85% performance after five acid regeneration cycles. The benefit of cellulose is its ability to immobilize fine HAp particles in a flexible, reusable form, preventing nanoparticle loss and enabling high-efficiency Sr^2+^ capture even in ion-rich environments.

Xu *et al.*^218^ developed quaternary ammonium-functionalized cellulose for pertechnetate (TcO_4_^−^) recovery, addressing the challenge of removing this highly mobile anion from aqueous nuclear waste. Regenerated cellulose was modified *via* reaction with glycidyltrimethylammonium chloride (GTMAC) under alkaline conditions, introducing permanent positive charges. FTIR confirmed quaternary ammonium groups, and SEM indicated preserved fibrous morphology. Batch adsorption experiments showed >93% TcO_4_^−^ removal efficiency at pH 6.0 with a Langmuir capacity of 122 mg g^−1^. The sorbent retained >87% of its capacity after five regeneration cycles using 0.1 M NaCl. The benefit of cellulose is its environmentally friendly backbone, which can be readily functionalized for anion exchange, yielding safe, reusable sorbents for nuclear wastewater decontamination.

Zhang *et al.*^219^ synthesized phosphorylated cellulose aerogels for uranium(vi) recovery from low-concentration waters, aiming to maximize surface area and ligand density. They produced cellulose aerogels *via* freeze-drying regenerated cellulose gels, then phosphorylated the aerogels using phosphoric acid and urea. BET analysis showed a surface area of 325 m^2^ g^−1^, and FTIR confirmed phosphate group incorporation. Adsorption tests revealed a maximum U(vi) capacity of 228 mg g^−1^ at pH 5.5, with >90% removal achieved within 15 minutes. Aerogels maintained >85% of capacity after five regeneration cycles with Na_2_CO_3_ solution. The benefit of cellulose here is its ability to form ultralight, highly porous aerogels that provide rapid mass transfer and high ligand accessibility for effective uranium capture.

Li *et al.*^220^ developed a cellulose-poly(acrylic acid) (PAA) hydrogel for uranium(vi) and thorium(iv) recovery, addressing the challenge of synthesizing a sorbent with high swelling capacity and abundant chelation sites. Regenerated cellulose was blended with acrylic acid monomer, followed by *in situ* free-radical polymerization using *N*,*N*′-methylenebisacrylamide as a crosslinker and ammonium persulfate as an initiator. FTIR confirmed the incorporation of –COOH groups from PAA, and SEM revealed a highly porous hydrogel structure. Adsorption studies showed maximum U(vi) capacity of 240 mg g^−1^ and Th(iv) capacity of 225 mg g^−1^ at pH 5.5. The hydrogel retained >85% of its capacity after five regeneration cycles using Na_2_CO_3_ solution. The benefit of cellulose here is its renewable backbone, which enhances hydrogel structural stability and synergistically interacts with PAA, yielding a high-capacity sorbent for multi-ion recovery.

Zhao *et al.*^221^ designed cellulose nanofiber-iron oxide (Fe_3_O_4_) composites for arsenic(v) and uranium(vi) recovery from contaminated groundwater, addressing the challenge of selective oxyanion adsorption at neutral pH. TEMPO-oxidized cellulose nanofibers (TOCNFs) were combined with Fe_3_O_4_ nanoparticles *via* co-precipitation of Fe^2+^/Fe^3+^ salts in alkaline media. FTIR confirmed carboxyl-iron coordination, and SEM-EDX showed uniform Fe distribution. Batch experiments showed U(vi) capacity of 198 mg g^−1^ and As(v) capacity of 172 mg g^−1^ at pH 7.0, with fast equilibrium times (<60 min). Regeneration using NaOH preserved >88% of capacity after five cycles. The benefit of cellulose here is its high surface area and functionalizable carboxyl groups, which anchor Fe_3_O_4_ nanoparticles for strong oxyanion binding while enabling magnetic separation for operational efficiency.

Liu *et al.*^222^ synthesized cellulose-polyethyleneimine (PEI) aerogels for uranium(vi) recovery from seawater, addressing the challenge of high salinity and low uranium concentration. Regenerated cellulose gels were freeze-dried to form aerogels, then impregnated with branched PEI *via* glutaraldehyde crosslinking. FTIR confirmed amine incorporation, and BET analysis revealed a surface area of 310 m^2^ g^−1^. Adsorption tests indicated U(vi) capacity of 210 mg g^−1^ in artificial seawater at pH 6.0. Aerogels retained >87% performance after five Na_2_CO_3_ regeneration cycles. The benefit of cellulose aerogels is their ultralight, porous structure, enabling rapid diffusion and high ligand loading for trace uranium capture.

Fan *et al.*^223^ developed phosphorylated cellulose-graphene oxide (GO) nanocomposites for uranium(vi) and thorium(iv) recovery, tackling the challenge of combining high sorption capacity with structural stability in flow systems. Regenerated cellulose was blended with GO in suspension, followed by phosphorylation using phosphoric acid and urea. FTIR confirmed phosphate incorporation, and SEM-EDX analysis showed uniform GO dispersion. Adsorption tests revealed U(vi) capacity of 238 mg g^−1^ and Th(iv) capacity of 220 mg g^−1^ at pH 5.5. The composite maintained >88% capacity after five regeneration cycles with Na_2_CO_3_. The benefit of cellulose is its hydrophilic nature and compatibility with GO, which together produce a hybrid material with improved mechanical strength, ligand density, and dispersion stability for radionuclide recovery.

Wang *et al.*^224^ synthesized carboxymethyl cellulose (CMC)-grafted polyacrylonitrile (PAN) fibers for uranium(vi) recovery from seawater, solving the challenge of producing mechanically robust sorbents with high amidoxime content. Electrospun PAN fibers were grafted with CMC to enhance hydrophilicity, followed by amidoximation with hydroxylamine hydrochloride. FTIR confirmed the formation of amidoxime, and SEM revealed a preserved fibrous structure. Adsorption studies showed U(vi) capacity of 265 mg g^−1^ in simulated seawater at pH 6.0. Fibers maintained ∼87% capacity after five Na_2_CO_3_ regeneration cycles. The benefit of cellulose is that CMC improves wettability and ion diffusion in the fibrous network, boosting uranium uptake efficiency in marine environments.

Qiu *et al.*^225^ designed cellulose-zeolitic imidazolate framework-8 (ZIF-8) composites for the recovery of cesium(i) and strontium(ii), addressing the challenge of embedding fragile MOFs into stable, reusable matrices. ZIF-8 nanoparticles were synthesized and incorporated into regenerated cellulose hydrogels before freeze-drying. XRD confirmed ZIF-8 crystallinity, and SEM showed uniform distribution within the cellulose network. Adsorption tests indicated Cs(i) capacity of 150 mg g^−1^ and Sr(ii) capacity of 145 mg g^−1^ at pH 6.5. Regeneration with 0.1 M KCl retained >85% capacity after five cycles. The benefit of cellulose is its robust, renewable framework that protects MOFs from mechanical damage and leaching, enabling repeated use in nuclear wastewater remediation.

Li *et al.*^226^ prepared bacterial cellulose–chitosan composite membranes for uranium(vi) and cobalt(ii) recovery, aiming to address the challenge of combining strength, flexibility, and multi-ion sorption. Bacterial cellulose pellicles were impregnated with chitosan solution, followed by crosslinking with glutaraldehyde. FTIR confirmed retention of the amine group, and SEM revealed a dense, layered membrane structure. Adsorption studies showed U(vi) capacity of 220 mg g^−1^ and Co(ii) capacity of 185 mg g^−1^ at pH 5.5. Membranes maintained >88% performance after five regeneration cycles with Na_2_CO_3_ (for U) or HCl (for Co). The benefit of cellulose here is its nanofiber strength and compatibility with biopolymers, enabling the production of sustainable, multifunctional membranes for the simultaneous recovery of radionuclides and transition metals.

Sun *et al.*^227^ developed cellulose acetate-silica hybrid beads for uranium(vi) recovery from acidic leachates, focusing on increasing surface area while retaining mechanical stability under acidic conditions. Cellulose acetate was dissolved in acetone, mixed with tetraethyl orthosilicate (TEOS), and gelled into beads by dropping into water, followed by hydrolysis and condensation of silica within the bead matrix. SEM revealed a porous surface with silica domains, while FTIR confirmed Si–O–Si bonding. Adsorption tests showed a maximum U(vi) uptake of 205 mg g^−1^ at pH 4.0, and the beads retained ∼88% of their capacity after five regeneration cycles with Na_2_CO_3_ solution. The benefit of cellulose acetate is its solvent-processability, which enables the formation of stable, porous hybrids that combine the flexibility of biopolymers with the stability of inorganic silica for uranium capture.

Wang *et al.*^228^ synthesized phosphorylated bacterial cellulose aerogels for the recovery of thorium(iv) and uranium(vi), targeting the challenge of achieving ultra-high surface area with strong acid-resistant functional groups. Bacterial cellulose pellicles were freeze-dried to produce aerogels and phosphorylated with phosphoric acid/urea under controlled heating. BET analysis indicated a surface area of 355 m^2^ g^−1^, and FTIR confirmed phosphate group attachment. Adsorption experiments gave maximum Th(iv) capacity of 240 mg g^−1^ and U(vi) capacity of 250 mg g^−1^ at pH 4.0. Aerogels maintained >90% performance after five cycles of Na_2_CO_3_ regeneration. The benefits of bacterial cellulose include its nanofiber purity, high crystallinity, and adaptability to aerogel formats, enabling efficient functionalization and exceptional sorption performance.

Zhang *et al.*^229^ produced thiol-functionalized carboxymethyl cellulose (CMC) cryogels for palladium(ii) and uranium(vi) recovery, addressing the challenge of integrating multiple functional groups for selective sorption of precious and radioactive metals. CMC was crosslinked with epichlorohydrin at subzero temperatures to form cryogels, then modified with thiourea. FTIR confirmed C=S bonds, and SEM revealed an interconnected macroporous structure. Adsorption studies indicated Pd(ii) capacity of 270 mg g^−1^ and U(vi) capacity of 220 mg g^−1^ at pH 5.5. The cryogels retained ∼85% of their capacity after regeneration with a thiourea-HCl solution. The benefit of cellulose here is its ability to form macroporous cryogels that allow rapid solution flow and high ligand accessibility, making them excellent for fixed-bed column applications.

Kong *et al.*^230^ developed cellulose-titanium dioxide-graphene oxide (TiO_2_-GO) ternary composites for strontium(ii) recovery, addressing the challenge of combining photocatalytic activity with adsorption. Regenerated cellulose films were impregnated with TiO_2_ nanoparticles and GO nanosheets *via in situ* hydrothermal synthesis. XRD confirmed anatase TiO_2_, and SEM showed uniform dispersion of TiO_2_ and GO within the cellulose network. Adsorption experiments yielded Sr^2+^ capacity of 150 mg g^−1^ at pH 7.0, with photocatalytic self-cleaning capability under UV light. The benefit of cellulose here is its film-forming ability and compatibility with both metal oxides and carbon nanomaterials, enabling multifunctional composites that both adsorb radionuclides and regenerate photochemically.

Yu *et al.*^231^ developed cellulose-polyethyleneimine-zirconium (Cell-PEI-Zr) composites for the co-recovery of fluoride and uranium(vi) from groundwater, addressing the challenge of simultaneously removing both contaminants. Regenerated cellulose was coated with PEI, then loaded with zirconium oxychloride followed by precipitation. FTIR confirmed amine and Zr–O bonds, and SEM showed uniform coating on cellulose fibers. Adsorption studies revealed U(vi) capacity of 230 mg g^−1^ and fluoride capacity of 210 mg g^−1^ at pH 5.5. The composite retained >85% capacity after five NaOH regeneration cycles. The benefit of cellulose is its abundant hydroxyl groups for PEI anchoring, which provide a stable platform for zirconium loading and impart strong affinity for both uranium and fluoride ions.

## 8. Carrageenan and its derivatives as multifunctional radioactive ions removal

Carrageenan, extracted from red seaweeds (Rhodophyta), is distinguished among polysaccharides by its high density of sulfate ester groups (–OSO_3_^−^), which strongly influence its physicochemical and adsorption properties. Commercial carrageenan exists in three principal forms: κ-carrageenan, containing one sulfate group per disaccharide and forming strong gels with K^+^ ions; ι-carrageenan, containing two sulfate groups and producing elastic Ca^2+^-mediated gels; and λ-carrageenan, containing three sulfate groups and existing mainly as a highly viscous non-gelling polymer. The sulfate groups, with p*K*_a_ values around 1.5–2.0, remain negatively charged even under strongly acidic conditions, providing carrageenan with a significant advantage over carboxylated polysaccharides such as alginate and pectin in acidic radioactive waste streams. In addition, sulfate oxygens behave as hard Lewis bases capable of strongly coordinating hard Lewis acid radionuclides, including UO_2_^2+^, Th^4+^, and lanthanide ions such as Eu^3+^ and Ce^3+^. Despite these favorable characteristics, carrageenan has received considerably less attention in radionuclide removal research compared with chitosan and alginate, with fewer than 15 reported studies. This limited focus is mainly attributed to its relatively higher cost, weaker mechanical gel strength, and the dominance of chitosan-based systems in the biosorption field. Nevertheless, carrageenan's exceptional acid stability and high sulfate functionality make it a promising specialized material for selective radionuclide removal in harsh nuclear wastewater environments.

Ijaz *et al.*^232^ aimed to design a multifunctional sorbent capable of simultaneously removing radioactive heavy metals and pharmaceutical contaminants from wastewater, with a specific focus on thorium (Th(iv)) and uranium (U(iv)). The central challenge addressed in this study was the need for a sorbent system that can efficiently recover radioactive ions from complex aqueous environments containing both inorganic and organic pollutants. Many existing adsorbents either lack selectivity for actinide ions or exhibit low adsorption capacities when multiple contaminants are present, and reusability is often compromised by structural degradation during regeneration cycles. To overcome these limitations, the authors synthesized a composite material, MXene@i.Carr@MaMb, by functionalizing MXene nanosheets with iota-carrageenan, maleic anhydride, and *N*,*N*′-methylenebisacrylamide. The methodology involved a multi-step grafting process: (1) surface activation of MXene to expose hydroxyl, fluorine, and oxygen groups; (2) covalent bonding of maleic anhydride and *N*,*N*′-methylene bis-acrylamide to enhance stability and introduce amide/carbonyl functionalities; and (3) integration of iota-carrageenan to confer biocompatibility and introduce ester sulfate groups known for strong coordination with actinide cations. The composite structure was characterized using FTIR, SEM, and XPS to confirm the presence of functional moieties and assess the binding environment. Adsorption experiments revealed that at 323.15 K, the material achieved sorption capacities of 3.6 ± 0.03 mmol g^−1^ for Th(iv) and 3.7 ± 0.09 mmol g^−1^ for U(iv), outperforming most conventional biosorbents. Sorption kinetics followed a pseudo-first-order model, and equilibrium data fitted well to Langmuir and Sips isotherms, suggesting monolayer adsorption with some heterogeneity. The optimum results were achieved at mildly acidic pH values, which favored electrostatic attraction and complexation between the actinide cations and the composite sulfate, hydroxyl, and carbonyl groups. Reusability tests demonstrated that the MXene@i.Carr@MaMb retained over 90% of its original capacity after multiple adsorption–desorption cycles, highlighting its structural stability. The primary benefit of using iota-carrageenan in this hybrid system lies in its dense network of sulfate ester groups, which have a high affinity for multivalent metal ions, combined with its natural abundance, biodegradability, and low cost. Its biopolymer nature also facilitates compatibility with other functional components without toxic byproducts, making it an environmentally favorable choice for nuclear wastewater remediation. This study thus provides a clear demonstration of how carrageenan derivatives can be integrated into advanced nanocomposite frameworks to achieve high-efficiency radioactive ion recovery even in multi-contaminant water streams.

Chuanyi Ma *et al.*^233^ conducted a study aimed at developing a high-performance, environmentally friendly hydrogel system for the removal of surface radioactive uranium(vi) contamination, particularly from diverse substrates encountered in nuclear facilities. The primary challenge addressed was that traditional colloid-based decontaminants often exhibit long curing times, low mechanical integrity, and suboptimal decontamination efficiency, especially on porous or rough surfaces. To overcome these limitations, the authors designed a peelable hydrogel coating composed of κ-carrageenan, konjac glucomannan (KGM), and graphene oxide (GO), capitalizing on the synergistic combination of biopolymer film-forming ability and nanomaterial reinforcement. The methodology involved dissolving κ-carrageenan and KGM in water to form a polysaccharide matrix, followed by uniform dispersion of graphene oxide sheets into the hydrogel precursor *via* ultrasonication to ensure nanoscale homogeneity. Upon application to contaminated surfaces, the hydrogel formed a cohesive, flexible film that adhered well during curing but could be peeled off easily afterward, carrying the adsorbed radionuclides with it. Characterization using FTIR, SEM/EDS, and XPS confirmed that GO introduction enhanced crosslink density *via* hydrogen bonding with the polysaccharide chains, improved thermal stability, and increased surface functional sites for uranium coordination. The decontamination performance was notable: after 30 minutes of application, removal efficiencies reached 96.95% on stainless steel, 96.27% on glass, 87.6% on ceramics, and 74.5% on rubber surfaces. On cement, a more challenging porous material, the maximum decontamination efficiency achieved was 70.68% after 24 hours. Mechanistically, the uranium removal was attributed to a combination of surface wetting, ion diffusion into the hydrogel, electrostatic attraction between U(vi) cations and negatively charged sulfate groups in κ-carrageenan, and physical entanglement within the gel matrix. The optimum performance was observed at ambient temperatures, with GO content tuned to balance mechanical strength and film flexibility. The benefit of incorporating κ-carrageenan lies in its natural abundance, sulfate-rich structure for selective uranium binding, and inherent biodegradability, making the formulation both cost-effective and environmentally benign. Furthermore, κ-carrageenan gel-forming capability ensures strong film cohesion without the need for toxic crosslinkers, a significant advantage over synthetic polymer systems. This work demonstrates that a carrageenan-based composite hydrogel reinforced with GO can serve as a practical, sustainable solution for nuclear facility decontamination, offering high efficiency, versatility across surface types, and simple waste handling *via* peelable removal.

Levy-Ontman *et al.*^234^ aimed to investigate the potential of iota-carrageenan as a sustainable bio-adsorbent for the targeted recovery of europium (Eu^3+^) ions from aqueous solutions, addressing a critical challenge in rare earth element recycling and radioactive waste management. Europium, although primarily known as a rare-earth element used in phosphors and nuclear control rods, can also be found in radioactive waste streams, making its removal important for environmental safety and resource recovery. The main challenge is that traditional ion-exchange and solvent-extraction processes for europium recovery are often energy-intensive, expensive, and reliant on synthetic chemicals that generate secondary pollution. To offer an eco-friendly alternative, the authors explored iota-carrageenan rich sulfate ester groups, known to have a high affinity for multivalent metal cations, as the functional basis for metal binding. The methodology involved preparing hydrogels of iota-carrageenan and two comparative polysaccharides (guar gum and xanthan gum), then exposing them to aqueous europium nitrate solutions under controlled pH and temperature conditions. The adsorption process was characterized using Fourier-transform infrared spectroscopy (FTIR), scanning electron microscopy (SEM), and X-ray photoelectron spectroscopy (XPS) to elucidate functional group interactions and changes in surface morphology. The study revealed that europium adsorption occurred primarily *via* coordination to ester sulfate groups, as indicated by the europium-to-sulfur atomic ratio (∼2.8 : 1) derived from EDS analysis. XPS data further showed significant changes in Eu^2+^/Eu^3+^ peak ratios after binding, suggesting partial reduction and complexation during sorption. Optimal adsorption yields were achieved at pH values at which sulfate groups were deprotonated, thereby enhancing electrostatic attraction. The iota-carrageenan hydrogel demonstrated superior performance compared to the other polysaccharides, with its sorption efficiency directly correlated to the acidity of its functional groups. The benefit of using iota-carrageenan in this context lies not only in its high europium-binding capacity but also in its renewable, biodegradable nature and the absence of hazardous byproducts during synthesis and application. This makes it especially suitable for integration into green recovery processes for rare earth and radioactive ions, potentially in combination with membrane filtration or as a strippable hydrogel for contaminated surfaces. The study offers strong evidence that iota-carrageenan-based systems can serve as an effective, low-cost, and environmentally friendly platform for selective radionuclide recovery from aqueous environments.

Ma *et al.*^235^ set out to develop an innovative adsorbent capable of efficiently capturing uranium(vi) ions from aqueous media, particularly those encountered in nuclear waste management scenarios. The challenge motivating this work is that uranyl ions (UO_2_^2+^) are highly mobile in water, radiotoxic, and chemically stable, making their removal both urgent and technically demanding. Many conventional adsorbents lack the required capacity, selectivity, and reusability, especially under varying pH and temperature conditions. To address this, the researchers synthesized a κ-carrageenan/polyacrylamide (CG-PAM) semi-interpenetrating network (semi-IPN) bio-hydrogel *via* UV-initiated polymerization. The methodology began by dissolving κ-carrageenan in aqueous solution to leverage its sulfate and hydroxyl functionalities, followed by *in situ* polymerization of acrylamide in the presence of *N*,*N*′-methylenebisacrylamide as a crosslinker. The UV irradiation step ensured rapid curing without high-temperature processing, preserving the integrity of the carrageenan chains. This semi-IPN structure provided a dual-functionality network: the hydroxyl and sulfate groups from carrageenan and the amide/carbonyl groups from polyacrylamide. The composite hydrogel performance was systematically tested for U(vi) removal across varying pH, temperature, and ion concentration conditions, with X-ray photoelectron spectroscopy (XPS) and density functional theory (DFT) calculations used to probe the binding mechanisms. Results showed that the optimal uranium uptake was 371.78 mg g^−1^ at pH 6.0 and 298.15 K, placing it among the highest capacities reported for biopolymer-based adsorbents. Thermodynamic analyses confirmed the adsorption was spontaneous and endothermic, while kinetic studies followed a pseudo-second-order model, indicating chemisorption as the dominant mechanism. DFT modeling revealed that uranium coordination strength followed the order: CO (PAM) > –OH (CG) > SO (CG) ≈ –O– (CG) > –NH_2_ (PAM), highlighting the synergistic contribution of carrageenan and PAM functional groups. The hydrogel retained over 95% of its capacity after six adsorption–desorption cycles, demonstrating remarkable stability and reusability. The benefit of employing κ-carrageenan lies in its renewable origin, its high density of sulfate groups for selective uranyl ion binding, and its compatibility with synthetic polymers, allowing mechanical enhancement without toxic additives. The resulting CG-PAM hydrogel thus offers a practical, scalable, and environmentally responsible solution for uranium recovery from contaminated water, combining high capacity, reusability, and structural resilience in a single system.

Radoor *et al.*^236^ initially aimed to design low-cost, eco-friendly membranes for the removal of cationic dyes from water; however, the underlying adsorption mechanisms and material properties demonstrated in their work make the system highly adaptable for radioactive ion recovery applications. The challenge addressed in their study, and relevant to nuclear waste remediation, is the development of a robust, reusable adsorbent capable of high-capacity ion removal without reliance on expensive synthetic polymers or hazardous chemical treatments. Traditional membranes often have limited affinity for multivalent cations and can foul or degrade after a few use cycles. The authors prepared polyvinyl alcohol (PVA)-based hydrogel membranes incorporating three types of carrageenan κ-, ι-, and λ-carrageenan by solution casting followed by controlled crosslinking. The methodology capitalized on carrageenan inherent sulfate ester groups, known for strong electrostatic attraction to positively charged ions, making it directly applicable to radionuclides such as U(vi), Th(iv), and Eu(iii). In their dye-removal experiments, PVA/κ-carrageenan membranes achieved the highest performance, with an adsorption capacity of 147.8 mg g^−1^ and removal efficiencies approaching 98.8%. Kinetic analysis indicated pseudo-second-order behavior, consistent with chemisorption, while isotherm fitting suggested monolayer adsorption on homogeneous active sites, with mechanistic features that parallel actinide binding behavior. The membranes maintained 98% removal efficiency even after five adsorption–desorption cycles, proving their mechanical integrity and regeneration capability. The optimum functional performance arose from κ-carrageenan single sulfate group per disaccharide unit, which reduced steric hindrance and allowed efficient ion-ligand coordination, a property that can be exploited for larger hydrated radioactive ions.

Furthermore, the presence of PVA provided a flexible yet stable polymer backbone, enhancing film strength and reducing carrageenan solubility in water, both of which are essential for prolonged contact with contaminated streams. The benefit of using carrageenan in such a hybrid membrane system lies in its renewable marine biomass origin, tunable sulfate density across its types, and compatibility with other polymers to tailor porosity and mechanical properties. While the original study targeted dye removal, the membrane structural and functional characteristics, especially its high sulfate availability, strong cation affinity, and excellent reusability, position it as a promising platform for radioactive ion recovery, potentially serving as a filtration-adsorption hybrid for nuclear effluent treatment.

Pujol Pozo *et al.*^136^ aimed to develop polysaccharide-based gels capable of forming strippable coatings for the efficient removal of radioactive contaminants from metal surfaces in nuclear facilities. The principal challenge addressed was the need for a decontamination technology that could effectively reduce surface radioactivity while minimizing secondary waste volume and avoiding corrosion or damage to sensitive equipment. Conventional chemical decontamination methods often require harsh reagents and generate large volumes of hazardous liquid waste, which pose additional handling and disposal challenges. In this study, the authors formulated gel-based coatings using various anionic polysaccharides, including pectin, sodium alginate, gum Arabic, xanthan gum, guar gum, and, importantly, carrageenan derivatives, chosen for their film-forming capabilities and strong binding affinity for multivalent cations. The preparation methodology involved dissolving the polysaccharide in aqueous solution, incorporating mild crosslinkers as needed, and casting the gel onto contaminated metal surfaces, including stainless steel, aluminum, Inconel, and Zircaloy. After drying, the coatings could be peeled off in intact sheets, physically removing radionuclide particles bound to the gel network. Decontamination factor measurements revealed that pectin-based gels achieved the highest performance; however, κ-carrageenan gels also demonstrated promising removal efficiencies due to their sulfate ester groups, which form stable complexes with cationic radionuclides such as Cs^+^, Sr^2+^, and UO_2_^2+^. The coating performance was dependent on the interaction between the polymer functional groups and the metal ion species present, as well as the coating adhesion to the substrate. Optimal results were obtained under conditions that allowed maximum gel flexibility without compromising peel strength, ensuring complete film removal without residue. The benefit of using carrageenan in such systems lies in its ability to form strong, cohesive films that maintain mechanical stability during both application and peeling, while also offering high ion-binding capacity *via* sulfate coordination chemistry.

Furthermore, carrageenan is renewable, biodegradable, and safe to handle, reducing both environmental and occupational hazards. Its natural gelation behavior enables formulation without toxic curing agents, and its compatibility with other polysaccharides or nanomaterials allows performance tuning for specific contaminants. This makes carrageenan-based strippable coatings a viable, eco-friendly option for surface radioactive decontamination, particularly in scenarios requiring rapid application, minimal waste, and high operational safety.

He and Chen^237^ presented a comprehensive review of biosorption of heavy metals using algal biomass, a topic directly relevant to radioactive ion recovery, given that many radionuclides, such as uranium, thorium, and europium, exhibit chemical behaviors similar to those of toxic heavy metals. Their work aimed to summarize the materials, performance, chemical interactions, and modeling tools associated with algal biosorption, thereby providing a foundation for the design of optimized biosorbents for environmental remediation. The central challenge highlighted was that while biosorption offers a low-cost, eco-friendly, and efficient approach for removing hazardous metals from aqueous solutions, there is a gap in translating these laboratory findings into scalable industrial applications, particularly for nuclear wastewater. One critical insight is that red algae, from which carrageenan is extracted, contain abundant sulfate ester and hydroxyl groups in their cell wall polysaccharides, enabling strong coordination and ion-exchange interactions with multivalent cations, including radioactive ions. The methodologies described in the reviewed studies used both raw and chemically modified algal biomass to improve sorption capacity and selectivity. Modifications such as crosslinking, grafting, and functional group enrichment were shown to enhance the density and accessibility of binding sites. The authors detailed that biosorption operates primarily *via* ion exchange, complexation, and micro-precipitation, with adsorption kinetics often following pseudo-second-order models and equilibrium described by Langmuir or Freundlich isotherms. In carrageenan-based systems, the high negative charge density of sulfate groups plays a critical role in attracting positively charged radionuclides. Optimal results for heavy metal removal were generally obtained in mildly acidic conditions, which balance proton competition with metal ion binding. Carrageenan, a purified derivative of red algal biomass, offers advantages over whole algal biomass by enabling processing into hydrogels, films, and composite adsorbents, facilitating easier handling, regeneration, and integration into engineered systems such as membrane-adsorbent hybrids or peelable coatings. The benefits of using carrageenan in biosorption-based radioactive ion recovery include its renewable marine origin, low production cost, chemical tunability, and environmental compatibility. Moreover, its compatibility with inorganic or nanostructured additives enhances mechanical stability and sorption performance, bridging the gap between raw biomass applications and advanced nuclear waste treatment technologies. This review underscores the strategic importance of carrageenan and other marine polysaccharides as foundational materials for next-generation, sustainable radionuclide recovery systems.

## 9. Pectin-based composite for radioactive ion removal

The adsorption of radionuclides onto advanced composite materials is governed by several simultaneous mechanisms, including ion exchange, electrostatic attraction, surface complexation, coordination interactions, pore confinement, and, in some cases, redox-assisted immobilization. Although many studies report high adsorption capacities and rapid kinetics, the relative contribution of these mechanisms strongly depends on the physicochemical properties of both the adsorbent and radionuclide species, as well as the complexity of the surrounding aqueous environment.

The previous literature focuses primarily on pectin-based composites for removing heavy metal ions (Cu^2+^, Pb^2+^, Cr(iii)) and organic dyes from wastewater. Only one study tangentially addresses radioactive ions: A. Kadam *et al.,* developed pectin-stabilized magnetic graphene oxide Prussian blue nanocomposites achieving 1.609 mmol g^−1^ adsorption capacity for cesium (a radioactive isotope), but this was not the primary research focus.

Pectin, primarily extracted from citrus peels and apple pomace, is a galacturonic acid-rich heteropolysaccharide whose adsorption behavior is strongly governed by the abundance of carboxylic groups (–COOH, p*K*_a_ ∼3.5) and the degree of methoxylation (DM). High-methoxyl pectin (HMP, DM > 50%) forms gels mainly through hydrogen bonding under acidic and high-sugar conditions, but its metal-binding performance is reduced because many carboxyl sites are blocked by methyl ester groups. In contrast, low-methoxyl pectin (LMP, DM < 50%) contains a larger fraction of free carboxylate groups (–COO^−^), enabling stronger coordination with radionuclides such as Cs^+^, Sr^2+^, and UO_2_^2+^. LMP additionally forms stable Ca^2+^-crosslinked hydrogels through the well-known “egg-box” mechanism, similar to alginate, which promotes cooperative binding of divalent ions while avoiding the use of organic crosslinking solvents. Although pectin shares several structural and functional similarities with alginate, it has received comparatively less attention in radionuclide remediation research, mainly due to its higher cost, lower abundance, and relatively weaker gel strength. Nevertheless, pectin possesses unique advantages in specific nuclear decontamination applications, particularly in the fabrication of strippable coatings for radioactive surface decontamination and in the preparation of Prussian blue-based composites for selective cesium removal.

Kadam *et al.*^238^ reported the facile synthesis of pectin-stabilized magnetic graphene oxide Prussian blue (PSMGPB) nanocomposites for the selective removal of radioactive cesium from aqueous solutions. In this work, pectin served as a stabilizing agent to effectively separate graphene oxide (GO) sheets and to improve the distribution of magnetite nanoparticles on their surfaces, thereby enhancing overall adsorption performance. The developed PSMGPB nanocomposites achieved a maximum cesium adsorption capacity of 1.609 mmol g^−1^, which was significantly higher than that of magnetic graphene oxide Prussian blue (1.230 mmol g^−1^), magnetic pectin Prussian blue (0.901 mmol g^−1^), and magnetic Prussian blue (0.330 mmol g^−1^) nanocomposites. The enhanced performance was attributed to the synergistic effect of pectin stabilization, which prevented GO sheet agglomeration and provided more accessible adsorption sites. Scanning electron microscopy confirmed the effective dispersion of GO sheets due to pectin incorporation. Adsorption experiments revealed that the optimal conditions for cesium removal were pH 7.0 and 30 °C. Thermodynamic analyses indicated that the adsorption process was spontaneous and exothermic. Equilibrium data were evaluated using isotherm models, and the non-linear regression results showed that the Langmuir isotherm provided the best fit, suggesting monolayer adsorption of cesium ions onto homogeneous active sites.

This study demonstrated that PSMGPB nanocomposites, by combining the high surface area and functional properties of graphene oxide, the strong cesium-binding affinity of Prussian blue, and the stabilizing effect of pectin, offer a promising and efficient material for the treatment of cesium-contaminated wastewater, especially in the context of nuclear accidents or radioactive waste management.

**Fig. 5:**
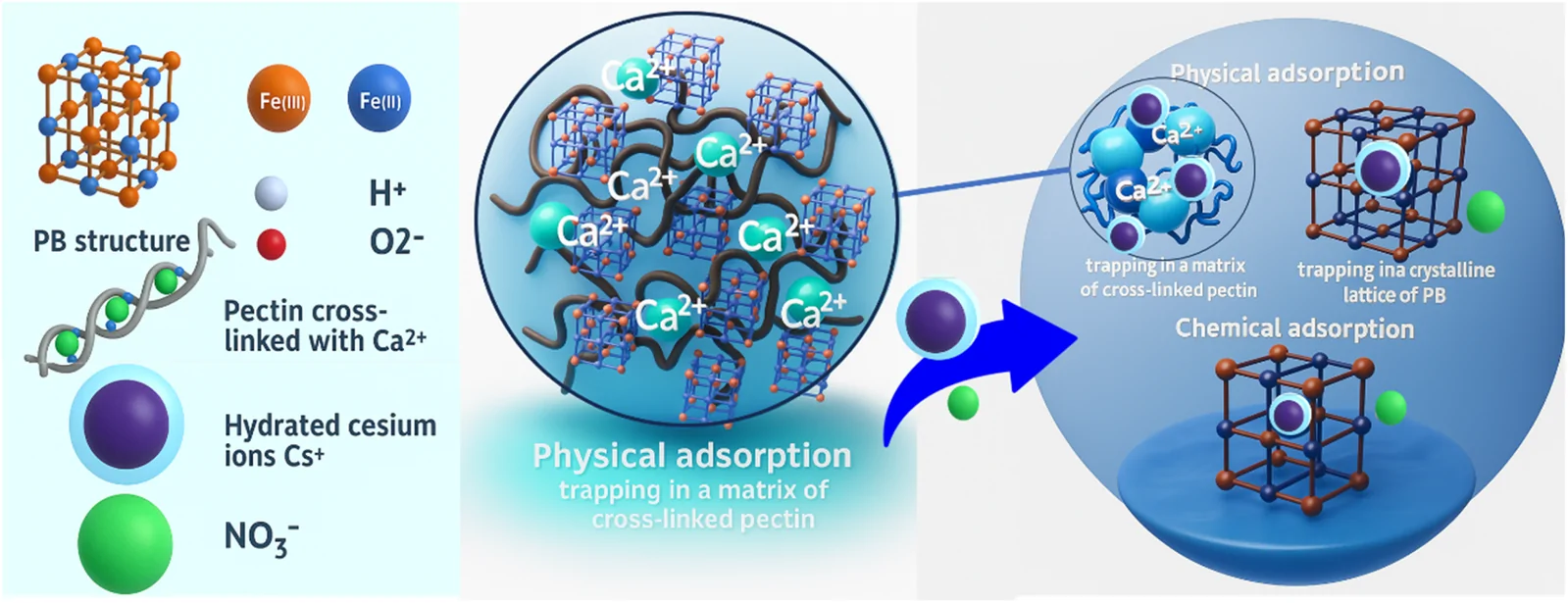
Schematic representation of cesium ion adsorption mechanisms in hybrid pectin/Prussian Blue composite beads. The left panel illustrates the overall bead structure showing Prussian Blue particles embedded within a calcium-crosslinked pectin matrix. The right panel details three distinct adsorption pathways: physical trapping within the pectin network, physical adsorption in Prussian Blue crystal lattice interstices, and chemical adsorption at lattice defects involving proton elimination from coordination water. The diagram includes a comprehensive legend identifying all ionic species and structural components, with color-coded spheres representing different atoms and ions within the system, this figure has been reproduced from ref. 239 with permission from Elsevier, copyright 2022.

Fig. 5 provides a comprehensive illustration of the cesium ion (Cs^+^) adsorption mechanisms occurring within hybrid pectin/Prussian Blue (PB) composite beads. This schematic illustrates the sophisticated multi-component system in which cesium ions are captured *via* multiple pathways, accounting for the enhanced sorption performance observed in these hybrid materials. The diagram is divided into two main sections: the left panel shows the overall structural organization of the hybrid bead, and the right panel details the specific molecular-level adsorption mechanisms.

The structural foundation of these hybrid sorbents consists of a three-dimensional pectin matrix cross-linked with calcium ions (Ca^2+^), within which discrete Prussian Blue particles are uniformly embedded. The pectin component forms a flexible, hydrogel-like network that serves both as a structural support and as an active sorption medium. Meanwhile, the Prussian Blue particles retain their characteristic face-centered cubic crystal structure, with alternating Fe(ii) and Fe(iii) centers connected by cyanide bridges. This dual-component architecture creates a heterogeneous environment with multiple types of binding sites, each contributing to the overall cesium uptake capacity.

The first adsorption mechanism illustrated involves physical trapping within the cross-linked pectin matrix. In this process, hydrated cesium ions become physically entrapped within the three-dimensional network formed by the calcium-crosslinked pectin chains. The binding occurs primarily through weak van der Waals forces and electrostatic interactions between the cesium cations and the negatively charged functional groups present in the pectin structure. This mechanism provides a relatively rapid, yet reversible, means of cesium capture, contributing to the sorbent system's initial uptake kinetics.

The second mechanism demonstrates physical adsorption within the crystalline lattice of Prussian Blue particles. Cesium ions are accommodated within the interstitial spaces and channels of the PB crystal structure, where they fit optimally due to their ionic radius being well-matched to the cavity dimensions. The Fe(ii)–CN–Fe(iii) framework creates a rigid three-dimensional network with specific sites that exhibit high selectivity for cesium ions over other competing cations. This physical incorporation into the crystal lattice represents the primary mechanism responsible for the high selectivity and capacity observed in PB-based sorbents.

The third and most chemically significant mechanism involves chemical adsorption at crystal lattice defects, accompanied by proton elimination from coordination water molecules. In this process, cesium ions undergo a substitution reaction, replacing protons associated with water molecules coordinated to the iron centers, particularly at defect sites within the PB crystal structure. This chemical exchange results in stronger bonds than simple physical adsorption, making cesium incorporation more thermodynamically favorable and less readily reversible. The release of protons during this process can be represented by the general reaction: Cs^+^ + H_2_O (coordinated) → Cs^+^ (incorporated) + H^+^ (released).

Fig. 5 clearly identifies the system's components. Orange spheres represent Fe(iii) centers, while blue spheres indicate Fe(ii) centers within the PB crystal structure. Purple spheres denote the hydrated cesium ions that are being adsorbed, green spheres represent nitrate anions present in the solution, and the light blue and red spheres correspond to protons and oxide ions, respectively. The black network structure illustrates the pectin chains cross-linked with calcium ions, while the cubic wireframe structures represent the Prussian Blue crystal lattice.

The synergistic effects observed in these hybrid systems arise from the complementary nature of the two components. The pectin matrix provides mechanical stability, prevents agglomeration of PB particles, and offers additional binding sites through its functional groups. Simultaneously, the embedded PB particles retain their inherent high selectivity and capacity for cesium ions while benefiting from improved accessibility afforded by their dispersion within the pectin network. This synergy results in sorption capacities that exceed those expected from a simple additive effect of the individual components, as evidenced by experimental data showing capacities of 38.7, 42.0, and 63.8 mg g^−1^ for the S1, S2, and S3 formulations, respectively.

The practical implications of this multi-mechanism approach are significant for real-world applications. The combination of rapid physical trapping in the pectin matrix, high-capacity, selective binding in PB crystals, and chemical adsorption at defect sites creates a robust, efficient cesium-removal system. The bead morphology (approximately 1–2 mm in the dry state, expanding to 4 mm when swollen) eliminates the handling and safety concerns associated with fine-powdered sorbents while maintaining high sorption performance. This makes hybrid sorbents particularly suitable for dynamic flow systems and practical remediation applications where both performance and operational safety are critical.

The gel-forming mechanism of pectin-based strippable coatings is fundamentally based on the unique structural properties of pectin, an anionic polysaccharide derived from plant cell walls. As documented in recent studies, pectin forms three-dimensional hydrophilic polymer networks, endowing hydrogels with softness, flexibility, and biocompatibility, and conferring exceptional attributes such as rapid gelation, higher melting points, and efficient carrier capabilities.

The primary mechanism underlying pectin gel formation involves the creation of junction zones through intermolecular associations. In low-methoxyl pectin systems, gelation occurs through ionic crosslinking where calcium ions or other divalent cations create bridges between negatively charged carboxyl groups on adjacent pectin chains. This ionic gelation mechanism is pH-dependent and requires sufficiently high concentrations of crosslinking ions to form stable gel networks.

High-methoxyl pectin systems utilize a different gelation mechanism based on hydrogen bonding and hydrophobic interactions. Under acidic conditions (pH < 3.5) and in the presence of co-solutes like sugars, the methyl ester groups on pectin chains interact through hydrogen bonds while the polymer backbone forms junction zones stabilized by hydrophobic associations. This mechanism allows gel formation without the need for divalent cations, making it suitable for applications where metal-ion interference must be minimized.

The formation of strippable coatings requires a careful balance between gel strength and removability. The crosslinking density must be sufficient to form a coherent film that can encapsulate contaminants while remaining flexible enough to peel off as an intact sheet. Optimization of pectin concentration, crosslinking agent levels, pH conditions, and drying parameters determines the final mechanical properties of the coating.

## 10. Polysaccharide-based strippable coatings for radioactive decontamination

Polysaccharide-based strippable coatings have emerged as an innovative and eco-friendly solution for decontaminating metal surfaces contaminated with radioactive materials. Starch is used for surface decontamination primarily because of its non-toxic, cost-effective, and biodegradable properties, making it an environmentally friendly option for managing radioactive contamination. In the study, a starch-based magnetic strippable hydrogel was developed to address the challenges of removing radioactive uranium from contaminated surfaces. The hydrogel was synthesized by incorporating Fe_3_O_4_ nanoparticles into a starch matrix through a one-pot polymerization method.^189^ This combination leverages starch's natural adsorption capacity for uranium and the magnetic properties of Fe_3_O_4_ for easy recovery of the decontamination material. The starch hydrogel forms a film when applied to contaminated surfaces. As the hydrogel dries, it becomes brittle and fractures, allowing it to be easily peeled or magnetically recovered, along with the adsorbed radioactive contaminants. The study demonstrated that the starch/Fe_3_O_4_ hydrogel achieved high decontamination efficiencies across various substrates, such as ceramic (89.62%), glass (81.33%), and steel (83.93%). However, its performance was lower on porous materials such as concrete (11.82%) due to deeper penetration of contamination. The hydrogel's shear-thinning behavior enabled easy spray application, while its magnetic properties facilitated efficient retrieval of the dried film, minimizing secondary waste. Starch's effectiveness in this application stems from its hydroxyl and carbonyl functional groups, which chemically interact with uranium ions, enabling their adsorption and subsequent removal. The study highlights starch's versatility and potential as a sustainable material for nuclear decontamination, offering a balance of high adsorption capacity, ease of application, and environmentally friendly recovery.

**Fig. 6:**
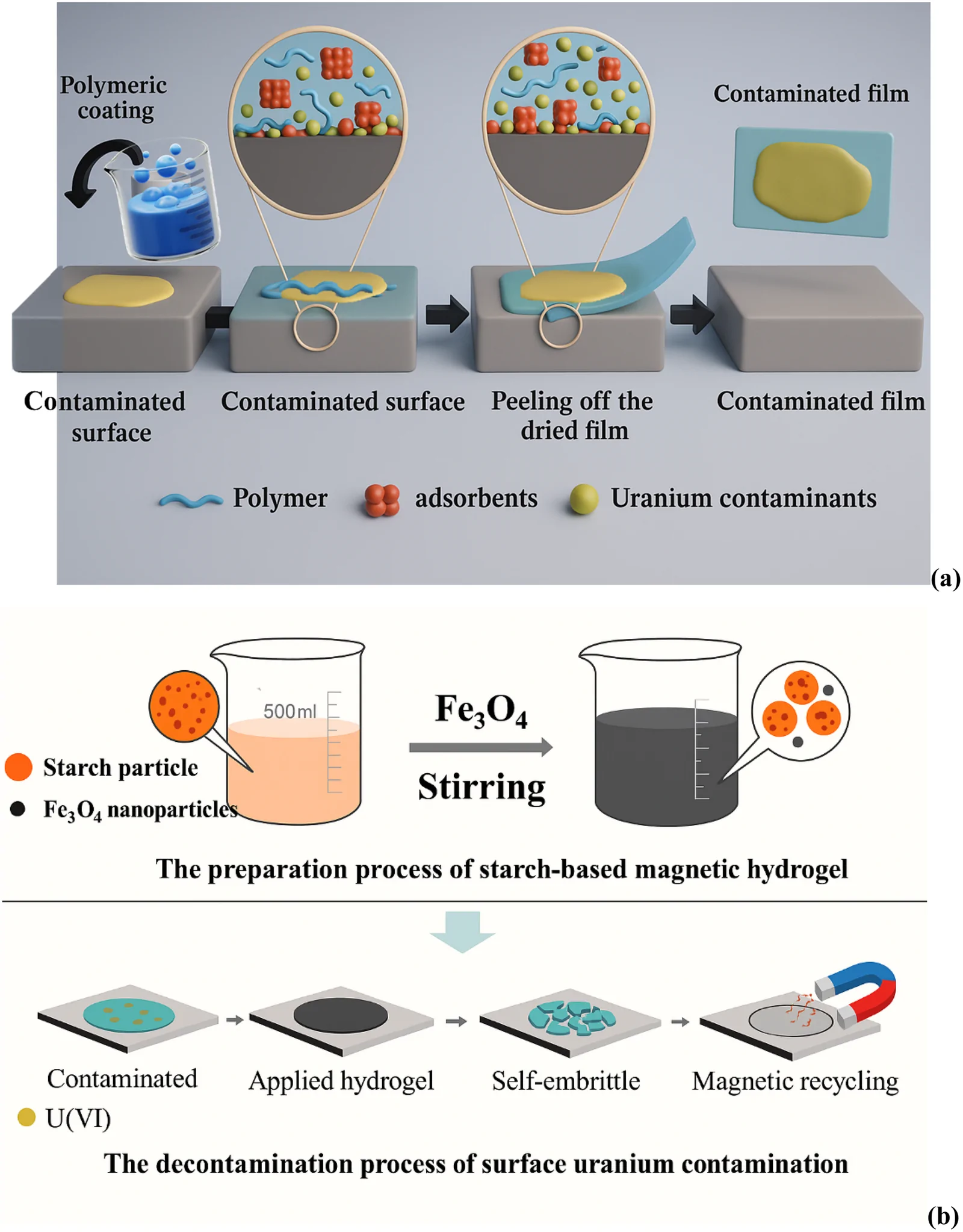
(a) Schematic illustration of the decontamination process of polymeric coating of strippable coating system for uranium removal from contaminated surfaces, this figure has been reproduced from ref. 240 with permission from MDPI, copyright 2022. (b) Schematic representation of starch-based magnetic hydrogel preparation and uranium decontamination process, this figure has been reproduced from ref. 189 with permission from Elsevier, copyright 2025.

Fig. 6a provides a detailed schematic illustration of the decontamination process utilizing an adsorbent-incorporated polyvinyl alcohol (PVA)/starch strippable coating system for uranium removal from contaminated surfaces. The process begins with the application of a viscous polymer solution containing dispersed adsorbent particles onto the uranium-contaminated substrate. The coating formulation is carefully engineered to achieve appropriate rheological properties, ensuring uniform coverage and intimate contact with the contaminated surface.^189^

The decontamination mechanism operates through multiple interaction pathways as the coating transitions from liquid to solid state. During the 12-hours ambient drying period, uranium contaminants become progressively incorporated into the polymer matrix through synergistic interactions with the PVA/starch polymer chains, embedded nano-fillers, and chelating agents present in the formulation. This multi-component approach ensures comprehensive capture of uranium species through various binding mechanisms including physical adsorption, chemical complexation, and polymer entanglement.

The final dried film represents a consolidated waste form that encapsulates the uranium contaminants within a stable polymer matrix. This strippable coating technology offers a particularly elegant solution for nuclear decontamination applications, as it transforms a surface contamination problem into a manageable solid waste form that can be easily removed and processed. The process is especially valuable for complex geometries or sensitive surfaces where traditional decontamination methods might be unsuitable or destructive.

In addition, Fig. 6b illustrates a two-stage process for radioactive decontamination using starch-based magnetic hydrogels. The upper portion demonstrates the hydrogel preparation methodology, in which starch particles in a 500 mL solution are combined with Fe_3_O_4_ nanoparticles under mechanical stirring, yielding a dark composite hydrogel containing both starch matrices and embedded magnetic nanoparticles. The lower section depicts the practical application of this magnetic hydrogel system for uranium surface decontamination, showing a sequential four-step process: initial surface contamination with U(vi) ions, application of the prepared magnetic hydrogel onto the contaminated surface, self-embrittlement of the hydrogel as it captures and immobilizes the radioactive contaminants, and finally magnetic recycling where an external magnet is used to collect and remove the brittle hydrogel pieces containing the concentrated uranium contamination. This innovative approach combines the biocompatible adsorption properties of starch with the magnetic recoverability of Fe_3_O_4_ nanoparticles, enabling efficient radioactive decontamination while facilitating easy collection and disposal of the contaminated material through magnetic separation, thereby minimizing secondary waste generation and exposure risks during cleanup operations.^189^

**Fig. 7:**
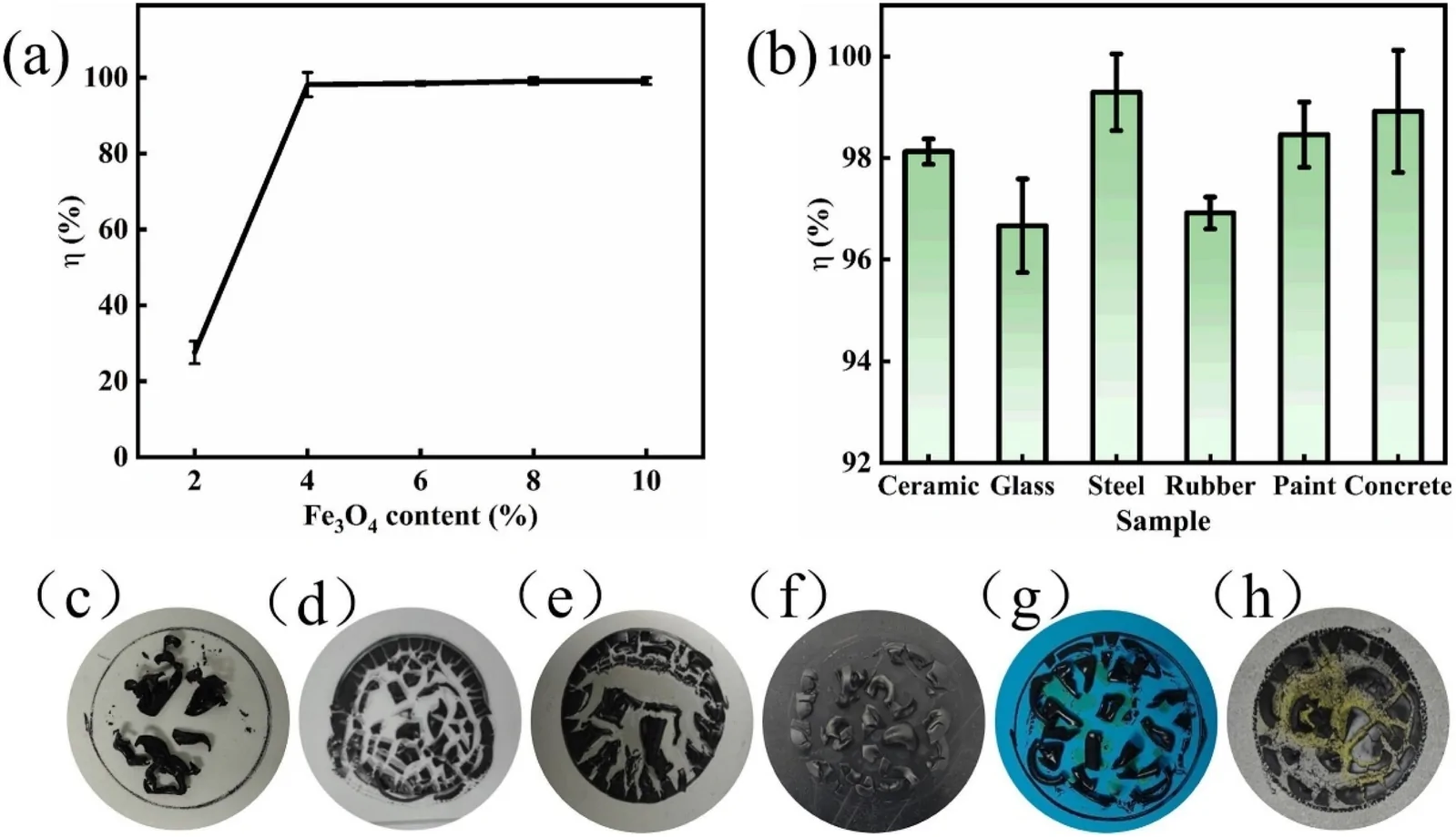
(a) Effect of Fe_3_O_4_ content on the recovery efficiency of dried starch/Fe_3_O_4_ hydrogel films. (b) Recovery efficiency of starch/Fe_3_O_4_-4% hydrogel on different substrates. (c–h) Brittle fracture behavior of starch/Fe_3_O_4_-4% hydrogel after drying on ceramic (c), glass (d), steel (e), rubber (f), paint (g), and concrete (h) surfaces, this figure has been adapted from ref. 189 with permission from Elsevier, copyright 2025.

Fig. 7a illustrates the effect of Fe_3_O_4_ content on the recovery efficiency of the dried starch/Fe_3_O_4_ hydrogel films. The results show that increasing the Fe_3_O_4_ loading enhances magnetic recovery, with efficiencies exceeding 96% at Fe_3_O_4_ contents of 4 wt% or higher. This confirms that incorporating Fe_3_O_4_ nanoparticles is crucial for enabling magnet-assisted retrieval of the brittle films after decontamination.

Fig. 7b presents the recovery efficiency of the starch/Fe_3_O_4_-4% hydrogel across different substrate materials, including ceramic, glass, steel, rubber, paint, and concrete. The recovery rates remain consistently high (above 96%) across all tested surfaces, demonstrating the hydrogel versatility in adhering to and detaching from various contaminated materials. Fig. 7c–h visually depict the brittle fracture behavior of the starch/Fe_3_O_4_-4% hydrogel after drying on different substrates. The images show that the hydrogel forms a uniform, crack-prone film upon dehydration, which facilitates easy detachment. The film's brittle nature allows it to fragment into pieces that can be efficiently collected with a magnet, as demonstrated in the application trials. This property ensures minimal surface residue after decontamination, reducing secondary waste.

Polysaccharide-based strippable coatings have emerged as an innovative and eco-friendly solution for decontaminating metal surfaces contaminated with radioactive materials. These coatings are derived from natural biopolymers such as pectin, sodium alginate, gum Arabic, and xanthan gum, which are abundant, biodegradable, and non-toxic. Their ability to form easily removable films makes them particularly attractive for nuclear industry applications, where minimizing secondary waste and reducing personnel radiation exposure are critical priorities.^136^ The decontamination process involves applying polysaccharide-based gels to contaminated surfaces. As the gel dries, it forms a peelable coating that encapsulates radioactive contaminants, which can then be safely removed and disposed of.

One of the key advantages of polysaccharide-based coatings is their ability to complex with radioactive cations *via* functional groups such as carboxyl (–COOH) and hydroxyl (–OH) groups. For example, pectin-based gels have demonstrated exceptional decontamination factors (DFs) for radionuclides like Co-58, Fe-59, and Nd-147, with DFs exceeding 1000 in some cases.^136^ The high efficiency of pectin is attributed to its low methoxyl content, which provides more free carboxyl groups for binding radioactive cations. Similarly, sodium alginate, another anionic polysaccharide, has shown promising results due to its high affinity for divalent and trivalent metal ions, making it suitable for decontaminating surfaces contaminated with fission and activation products.^136^

These coatings are versatile and can be applied to various surfaces, including stainless steel, aluminum, Zircaloy, and Inconel. For instance, pectin-based gels with propionic acid achieved DFs of ∼1800 for Nd-147 on stainless steel, while gum Arabic and xanthan gum formulations were effective for Co-58 and Sr-85.^136^ The choice of polysaccharide and additives (*e.g.*, antimicrobial agents such as nitric or propionic acid) can be tailored to target specific radionuclides or surface types, thereby enhancing decontamination efficiency. Moreover, the viscosity of the gel plays a crucial role in its application; pseudoplastic gels, such as xanthan gum, are ideal for vertical surfaces, while Newtonian fluids, such as gum Arabic, are better suited for spraying.^136^

**Fig. 8:**
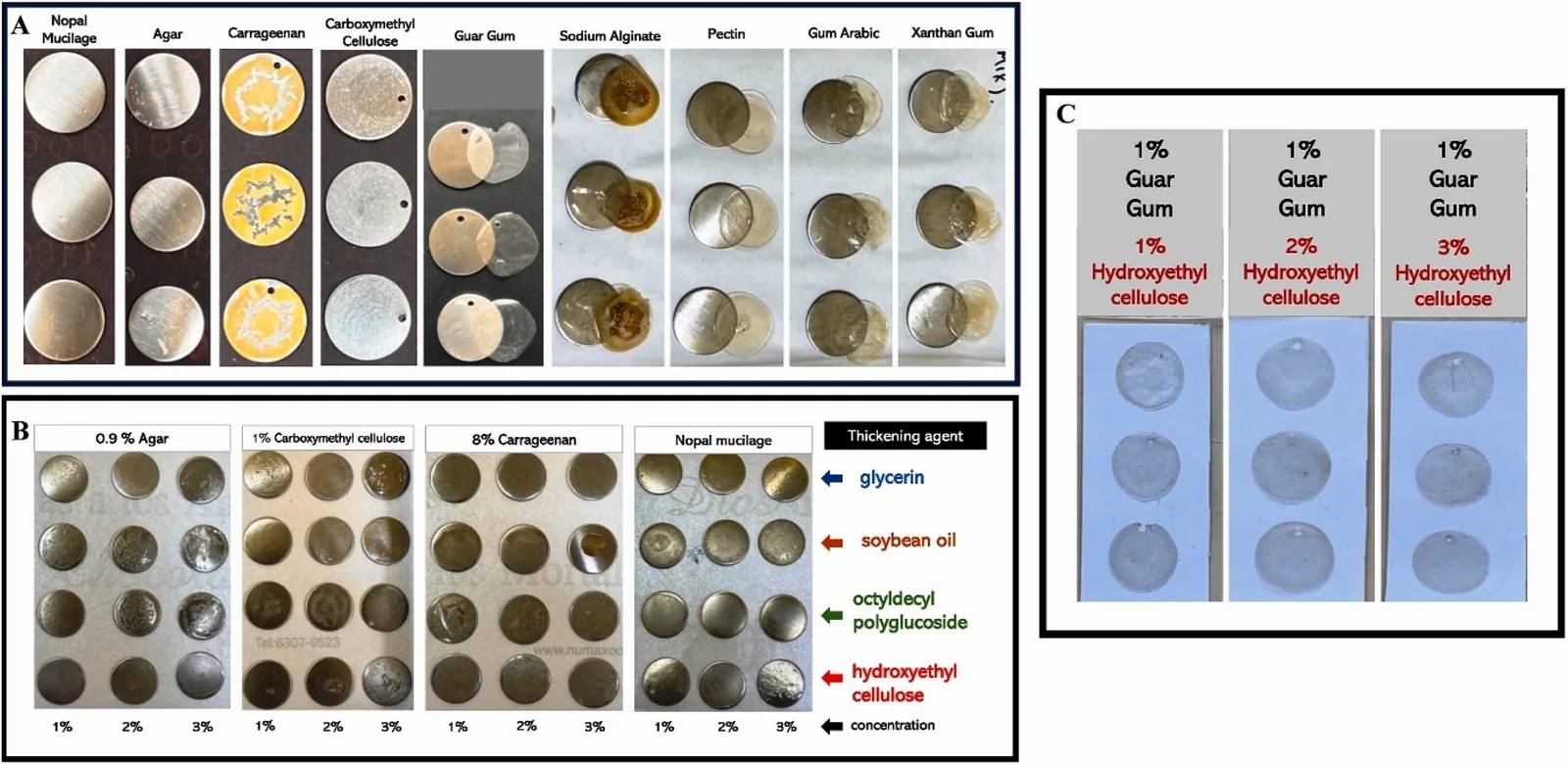
(A) Chemical structures of representative polysaccharides used in strippable coatings for radioactive decontamination pectin, sodium alginate, and xanthan gum highlighting functional groups (carboxyl, hydroxyl, pyruvyl, and acetyl) responsible for metal ion binding. (B) Schematic illustration of the decontamination mechanism showing gel application on a contaminated metal surface, radionuclide diffusion into the polysaccharide matrix, complex formation *via* carboxyl/hydroxyl groups, and peeling of the dried film to remove immobilized contaminants. (C) Comparative decontamination factors (DFs) of pectin, sodium alginate, gum Arabic, and xanthan gum coatings for radionuclides (Co-58, Fe-59, Sr-85, Nd-147) on stainless steel surfaces, with pectin-propionic acid formulation showing the highest efficiency for Nd-147, this figure has been adapted from ref. 136 with permission from Elsevier, copyright 2025.

Fig. 8a illustrates the molecular structures of three representative polysaccharides pectin, sodium alginate, and xanthan gum used in strippable coatings for radioactive decontamination. Pectin, a galacturonic acid-rich heteropolysaccharide, contains abundant carboxyl (–COOH) and hydroxyl (–OH) groups, enabling strong complexation with cationic radionuclides. Low-methoxyl pectin (LMP) offers superior performance due to its more accessible free carboxyl groups, which facilitate electrostatic attraction and coordination bonding. Sodium alginate, composed of β-d-mannuronate (M) and α-l-guluronate (G) units, possesses carboxylate (–COO^−^) moieties that selectively bind divalent and trivalent ions through an “egg-box” gel network. Xanthan gum, a branched polysaccharide, incorporates pyruvyl and acetyl substituents, enhancing its chelation capacity, though steric hindrance can limit ion accessibility. The presence, density, and accessibility of these functional groups are critical determinants of metal ion affinity and ultimately define the coating decontamination efficiency.^136,221^

Fig. 8b schematically represents the decontamination pathway for polysaccharide-based strippable coatings. The process begins with applying a gel to a contaminated metallic surface, enabling radionuclide diffusion into the hydrophilic polymer matrix. Functional groups primarily carboxyl (–COOH) and hydroxyl (–OH) deprotonate above their p*K*_a_ (*e.g.*, pectin p*K*_a_ ≈ 3.5), producing negatively charged sites (R-COO^−^) that electrostatically attract cationic species such as Co^2+^, Sr^2+^, and Nd^3+^. Simultaneously, coordination bonds form between the polysaccharide oxygen atoms and the metal ions, enhancing stability of the complexes. Surface penetration is facilitated by the gel wettability, allowing access to radionuclides embedded within oxide layers (*e.g.*, Cr_2_O_3_ on stainless steel). Once bound, the radionuclides remain immobilized within the drying polymer film. The final peeling step physically removes the contaminants, yielding a clean surface without the need for abrasive or corrosive treatments.^136^

Fig. 8c compares the decontamination factors (DFs) achieved by various polysaccharide-based coatings for radionuclides on stainless steel. Pectin modified with propionic acid (PRPA) demonstrates the highest DF (∼1800 for Nd-147), owing to its high density of ionized carboxyl groups at mildly acidic pH (∼4), which enhances complexation efficiency. Gum Arabic exhibits moderate efficiency for transition metals like Co-58 (DF ∼600), but often requires nitric acid additives to improve oxidative removal, potentially increasing the risk of substrate corrosion. Xanthan gum, despite its strong chelation potential, yields lower DFs (∼100 for Co-58) due to steric hindrance from its branched structure, which limits ion diffusion into the gel. Sodium alginate, applied in strongly alkaline solution (pH 14), provides moderate DFs (∼300 for U) through its “egg-box” mechanism but may damage sensitive metal surfaces under high pH conditions. These performance differences highlight the importance of molecular structure, functional group availability, and formulation chemistry in optimizing coating efficiency.^136^

**Fig. 9:**
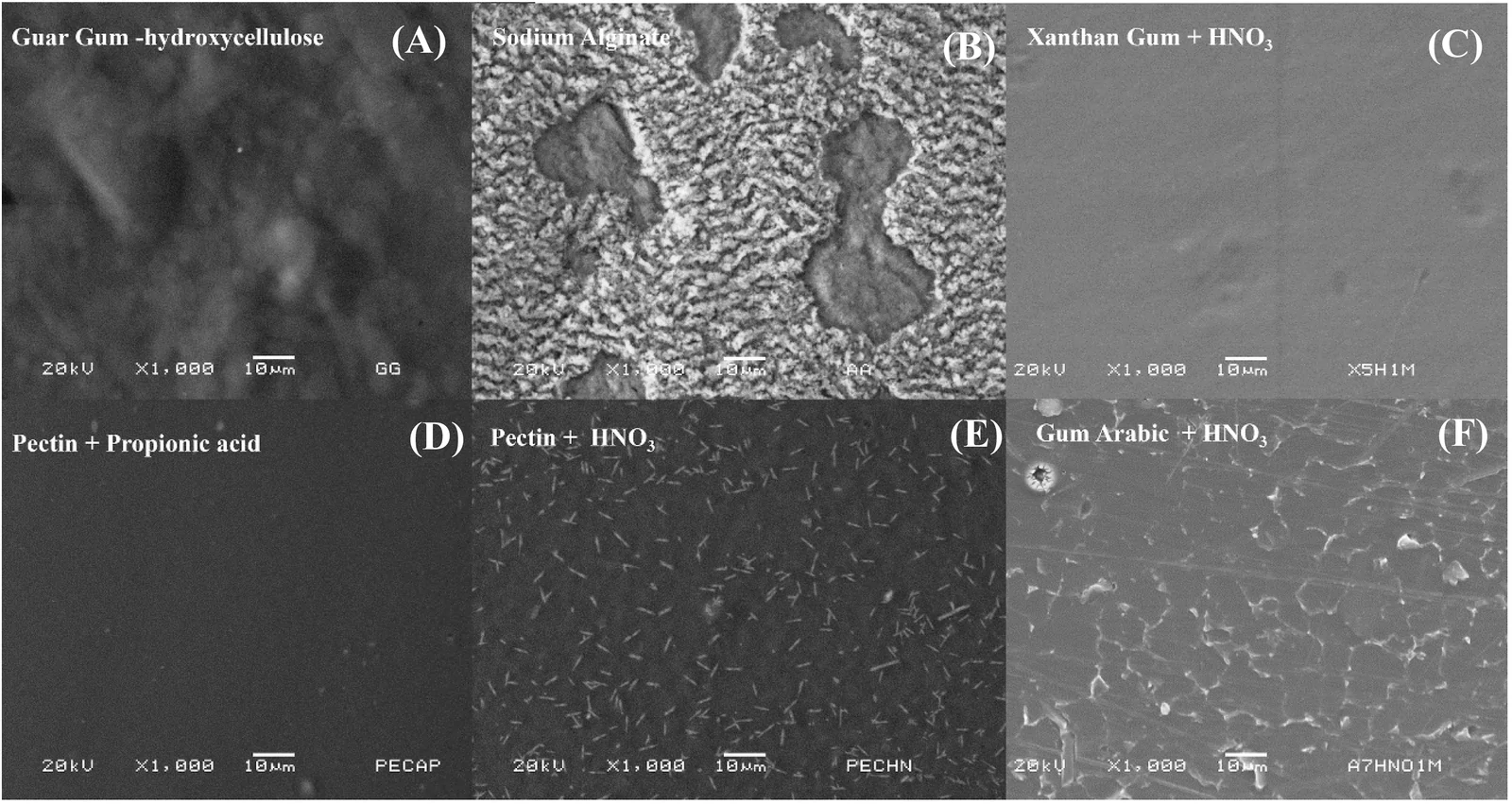
Optical micrographs of dried polysaccharide-based gel coatings illustrating distinct surface morphologies and their implications for decontamination performance: (A) 1% guar gum + 3% hydroxyethyl cellulose (GG/HEC) with phase-separated domains; (B) 6% sodium alginate (SAL) showing crystalline NaOH deposits within the matrix; (C) 5% xanthan gum + HNO_3_ (XGNA) exhibiting rough, heterogeneous surfaces from acid-induced hydrolysis; (D) 3% reagent-grade pectin + propionic acid (PRPA) displaying a smooth, homogeneous morphology with no visible precipitates or cracks. Scale bars: 50 µm; (E) 3% reagent-grade pectin + HNO_3_ (PRNA) containing needle-like NaNO_3_ crystals; (F) 7% gum Arabic + HNO_3_ (GANA) presenting micro-cracks from rapid drying and polymer degradation, this figure has been adapted from ref. 136 with permission from Elsevier, copyright 2025.

Fig. 9A displays the dried morphology of a 1% guar gum and 3% hydroxyethyl cellulose (GG/HEC) blend, showing clear phase separation. The darker regions correspond to the guar gum matrix, while the lighter granules are discrete HEC domains, indicating incomplete miscibility between the two polymers. This heterogeneity limits the uniform distribution of functional groups, reducing the surface area available for radionuclide complexation. As a result, the decontamination factor remains moderate (DF ∼200 for Co-58). HEC enhances the viscosity and pseudoplastic behavior of the gel, improving vertical surface coverage during application. However, it does not provide additional active binding sites, meaning radionuclide capture relies primarily on guar gum limited carboxyl and hydroxyl functionalities. The lack of homogeneous morphology leads to inconsistent performance, especially on rough surfaces. While GG/HEC blends are mechanically stable and easy to apply, their adsorption efficiency could be improved through chemical compatibilization or co-crosslinking strategies.^136^

Fig. 9B shows the microstructure of dried 6% sodium alginate (SAL) gel containing crystalline NaOH deposits embedded within the polymer matrix. The bright, crystalline regions indicate phase separation, with NaOH precipitating during drying. This high alkalinity (pH 14) facilitates the hydrolysis of surface oxides, such as Cr_2_O_3_ on stainless steel, enabling the release of adsorbed radionuclides. However, the poor cohesion between the crystalline NaOH and alginate network results in fragile films that peel unevenly, lowering decontamination efficiency (DF ∼300 for uranium). The residual NaOH poses a corrosion risk, especially for acid-sensitive substrates like aluminum. While the “egg-box” structure of alginate carboxylates can bind multivalent cations effectively, excessive NaOH compromises both film integrity and mechanical flexibility. Thus, although SAL offers strong chemical reactivity, its practical application in decontamination is limited by substrate compatibility and film cohesiveness.^136^

Fig. 9C reveals the dried microstructure of a 5% xanthan gum gel modified with nitric acid (XGNA), exhibiting a rough, heterogeneous surface and irregular aggregates. The strong oxidative environment of HNO_3_ partially hydrolyzes the polysaccharide backbone, producing fragmented domains and increasing surface porosity. While porosity may enhance contaminant entrapment, the branched structure of xanthan gum inherently restricts radionuclide diffusion to its carboxyl groups, reducing binding efficiency. Consequently, the decontamination factor is relatively low (DF ∼100 for Co-58). High viscosity enables stable application on vertical or inclined surfaces, but the rough texture weakens peelability, leaving residues upon removal. Additionally, the oxidative degradation from HNO_3_ can damage the polymer functional groups, further lowering ion-exchange capacity. Overall, while XGNA adheres well to various substrates, its structural limitations and chemical instability in strong acids limit its suitability for high-efficiency decontamination compared to smoother, chemically milder formulations.^221^

Fig. 9D illustrates the smooth, uniform surface of a 3% reagent-grade pectin gel modified with propionic acid (PRPA). Unlike acid formulations containing strong oxidizers, PRPA preserves the polymer integrity while maintaining optimal carboxyl group ionization (pH ∼4), enabling high-efficiency radionuclide complexation. This homogeneous, crack-free morphology maximizes contact between functional groups and contaminants, resulting in the highest recorded decontamination factor (∼1800 for Nd-147). The absence of crystalline deposits or phase separation contributes to excellent mechanical peelability, ensuring complete removal of the coating and immobilized radionuclides without residue. Propionic acid serves as a mild proton donor, enhancing adhesion to metal surfaces without degrading pectin structure, thereby balancing chemical effectiveness and physical robustness. As a result, PRPA is considered the optimal formulation for polysaccharide-based decontamination coatings, combining high chemical binding efficiency with reliable mechanical performance.^136^

Fig. 9E shows the dried morphology of a 3% reagent-grade pectin gel prepared with nitric acid (PRNA), revealing needle-like NaNO_3_ crystals embedded in the pectin matrix. These crystals form when sodium ions react with nitric acid during gel formulation. While the crystalline domains contribute to oxidative decontamination, enhancing removal of radionuclides such as Co-58 (DF ∼600), they also increase brittleness, making the coating prone to fragmentation during peeling. Despite this, the carboxyl groups of pectin remain largely intact, allowing effective cation binding through coordination and electrostatic interactions. The crystalline morphology can potentially enhance micro-scale contact with contaminants but risks generating loose fragments that may require secondary cleanup. Therefore, PRNA offers a balance of chemical reactivity and adsorption capacity but is less mechanically stable than smoother formulations, making it better suited for applications where oxidative decontamination is prioritized over peelability.^221^

Fig. 9F depicts the microstructure of a 7% gum Arabic gel prepared with nitric acid (GANA), characterized by a cracked surface morphology. These micro-cracks form during rapid drying, driven by polymer shrinkage and HNO_3_-induced chain degradation. While gum Arabic contains arabinogalactan backbones with potential carboxyl groups, nitric acid hydrolysis, as confirmed by FT-IR, reduces the number of available binding sites, thereby diminishing radionuclide capture capacity. As a result, decontamination performance is inconsistent, with DFs around ∼90 for uranium. The cracks also expose portions of the underlying contaminated surface, leading to incomplete removal during peeling. The strong oxidative environment of HNO_3_ can help break down certain surface contaminants but simultaneously compromises polymer cohesion. Improving GANA performance may require using milder acids or incorporating plasticizers, such as glycerol, to reduce brittleness. Without these modifications, GANA remains limited in applications demanding both high adhesion and mechanical robustness.^136^

The application of pectin-based strippable coatings in nuclear decontamination addresses several critical challenges in the industry. Traditional decontamination agents often involve harsh chemicals that pose environmental and health risks, generate secondary waste streams, and may damage underlying surfaces. Yang *et al.*^241^ addressed these concerns by developing novel biodegradable strippable coatings using new generation complexing agents, demonstrating that eight different types of complexing agents were employed for the development of new biodegradable decontamination solutions that can form strippable coatings after they are dispersed and allowed to dry on a variety of surfaces contaminated with 60Co, 133Ba, 137Cs, and 241Am.

The versatility of pectin-based coatings extends beyond nuclear applications to general heavy metal decontamination. Xue *et al.*^242^ developed aqueous strippable coatings based on adsorbent/polyvinyl alcohol polymer systems that demonstrated high performance and removability, flexibility, and compatibility with various substrates. Their work showed greater decontamination efficiency than commercial products such as DeconGel, achieving 87.2% at 5 g L^−1^ uranium and 95.5% at 22.5 g L^−1^ uranium.

## 11. Removal efficiency for various radionuclides

The decontamination efficiency of pectin-based strippable coatings varies significantly depending on the specific radionuclide, surface type, contamination level, and formulation parameters. Extensive research has demonstrated the effectiveness of these systems against a broad spectrum of radioactive contaminants commonly encountered in nuclear facilities and environmental remediation scenarios.

For cesium isotopes (137Cs), pectin-based coatings have shown remarkable performance due to the polysaccharide matrix's natural affinity for alkali metals. Yang *et al.*^241^ reported impressive results, achieving up to 95% decontamination factors for 137Cs on various surfaces using their biodegradable strippable coating formulations. The high efficiency of cesium removal is attributed to ionic interactions between cesium cations and the negatively charged carboxyl groups in the pectin matrix, with decontamination factors ranging from 10 to 100 depending on contact time, coating thickness, and the presence of competing ions.

Cobalt-60 removal has been successfully demonstrated using pectin coatings modified with specific chelating agents. Yang *et al.*^241^ showed that their new generation eco-friendly chelators achieved up to 95% decontamination factors for 60Co, demonstrating superior performance while maintaining biodegradability. Complementing these findings, Wang *et al.*^243^ developed polymeric gels that achieved decontamination efficiencies of up to 95% for both radionuclides (137Cs and 60Co) after 24 h of contact with the contaminated surface, suggesting that the decontamination process operates through combined chemical and physical mechanisms.

Uranium decontamination represents another significant application area for pectin-based systems. Xue *et al.*^242^ demonstrated exceptional uranium removal performance with their aqueous strippable coatings, achieving 87.2% and 95.5% removal efficiencies at 5 g L^−1^ and 22.5 g L^−1^, respectively, from stainless steel surfaces. The high efficiency of uranium is attributed to strong complexation between carboxyl groups in pectin and hexavalent uranium species, forming stable chelate complexes that are effectively removed during coating stripping.

Strontium and barium isotopes pose unique challenges because of their chemical similarity to calcium, which can interfere with calcium-crosslinked pectin systems. However, Yang *et al.*^241^ successfully addressed these challenges, achieving up to 95% decontamination factors for 133Ba using modified formulations with biodegradable strippable coating systems. Their work demonstrated that alternative crosslinking mechanisms or the incorporation of selective extractants can achieve effective removal of these alkaline earth metals.

Americium-241 and other transuranics are the most challenging contaminants due to their strong surface binding and complex chemical behavior. Palacios *et al.*^244^ expanded the scope of application by exploring peelable films derived from chitosan gels and chitosan/magnetite nanoparticle composites for the decontamination of metallic surfaces from radioactive contamination. Their work demonstrated that films of chitosan-based gels were evaluated on steel stainless and aluminum, clean and corroded, and weathered iron test pieces, showing the versatility of polysaccharide-based systems across different surface conditions and metal types.

## 12. Real-world applicability and life-cycle assessment

Polysaccharide adsorbents face critical radiation stability challenges that remain inadequately characterized for real-world nuclear waste deployment. While laboratory studies demonstrate high adsorption capacities, the radiation stability of polysaccharide-based adsorbents under actual nuclear waste conditions represents a significant gap between promise and field application. Bleyen Nele *et al.*^245,246^ provide the most direct evidence, exposing cellulosic tissues to gamma irradiation up to 1.4 MGy under both oxic and anoxic conditions. Their findings demonstrate radiation-induced chain scission causing decreased polymerization degree, cellulose amorphization at doses ≥0.8 MGy, and critically, accelerated alkaline degradation of pre-irradiated materials with isosaccharinic acid production rates far exceeding those from alkaline degradation alone. However, these studies focus on cellulose specifically rather than broader polysaccharide adsorbents, and systematic data across the full dose-rate spectrum (10^−3^ to 10^6^ Gy h^−1^) for different waste classes remains limited.^247^

### 12.1. Radiation stability of polysaccharide adsorbents under nuclear waste conditions

The deployment of polysaccharide-based adsorbents for nuclear waste treatment faces a fundamental and largely unaddressed challenge: the lack of comprehensive radiation stability data under realistic nuclear waste conditions. While extensive research has demonstrated impressive radionuclide adsorption capacities for modified polysaccharides under controlled laboratory conditions, the critical question of whether these materials can maintain their binding performance when exposed to the intense radiation fields present in actual nuclear waste streams remains largely unanswered.

L. An, *et al.*^248^ provides crucial context for understanding radiation-induced polysaccharide modification mechanisms. The review identifies that radiation technology can be used for controlled molecular modification of polysaccharides including chitosan, starch, carrageenan, sodium alginate, and cellulose. However, the distinction between controlled modification for beneficial purposes and uncontrolled degradation in nuclear waste environments is critical. While controlled radiation can sometimes improve certain properties, the uncontrolled, high-dose radiation in nuclear waste streams is more likely to cause detrimental effects. The mechanisms identified in the research question main-chain scission, crosslinking, and functional group destruction represent the primary pathways through which radiation compromises polysaccharide adsorbent performance. Main-chain scission cleaves glycosidic bonds, reducing molecular weight and mechanical strength while potentially causing physical disintegration of adsorbent beads or membranes. Crosslinking, while sometimes beneficial at low doses, can increase brittleness at high doses and may reduce the accessibility of binding sites. Functional group destruction directly attacks the chemical moieties responsible for radionuclide binding, such as amino groups in chitosan, sulfate groups in carrageenan, or carboxyl groups in alginate and pectin.

A. Bhaskarapillai, *et al.*^249^ documents the significant potential of chitosan-based sorbents in nuclear industry applications, highlighting the versatile binding properties and ease of modification that make chitosan attractive for radioactive and non-radioactive metal ion removal. However, this comprehensive review of chitosan applications in the nuclear industry notably lacks radiation stability data, exemplifying the knowledge gap identified in the research question. The review focuses extensively on adsorption capacities, selectivity, and chemical modifications, but does not address how these properties might change under radiation exposure. This omission is particularly significant given that chitosan's primary binding mechanism relies on amino groups that are susceptible to deamination under radiation. If a phosphorylated chitosan bead achieving 667 mg g^−1^ U(vi) capacity loses substantial binding sites due to radiation-induced deamination, its practical utility in nuclear waste treatment becomes questionable regardless of its initial performance. V. Luca & J. Veliscek-Carolan,^250^ provides valuable comparative insights through their study of metal(IV) phosphonate hybrid adsorbent materials. Their research demonstrated that zirconium phosphonate coordination polymers (Zr-ATMP) maintained highly efficient sorption from strongly acidic solutions even after receiving gamma radiation doses up to 2.9 MGy. Importantly, they used solid-state ^31^P MAS-NMR to probe radiolytic degradation mechanisms, finding that polyphosphonate-functionalized hybrids were more stable than monophosphonate hybrids due to stronger surface attachment. While these materials are inorganic-organic hybrids rather than pure polysaccharides, the study demonstrates the critical importance of testing actual sorption performance post-irradiation rather than merely measuring physical or chemical changes to the material structure. This approach represents exactly what is missing from polysaccharide adsorbent research direct measurement of functional performance retention after radiation exposure. H. Bonin *et al.*, *et al.*^251^ investigated polymer radiation resistance for radioactive waste disposal applications, studying thermoplastic polymers including polypropylene, nylon 66, polycarbonate, and polyurethane under mixed radiation fields up to 3.0 MGy. Their findings showed that these synthetic polymers could adequately resist radiation through predominant crosslinking mechanisms, with observed increases in density and Young's modulus but decreased strain at break. However, these synthetic polymers have fundamentally different structures and radiation response mechanisms compared to polysaccharides, limiting direct applicability of their findings. The evidence clearly indicates that the radiation stability of polysaccharide-based adsorbents represents the most significant barrier to their practical deployment in nuclear waste treatment. Future research must prioritize direct measurement of radionuclide binding capacity retention after radiation exposure at doses relevant to target waste streams. Studies should expose adsorbents to gamma doses of 10–1000 kGy, measure capacity retention post-irradiation, and test under simultaneous irradiation and adsorption conditions to capture *in situ* radiolysis effects. Additionally, research into mitigation strategies shows promise. The incorporation of radical scavengers or embedding polysaccharides within protective inorganic matrices could potentially extend their useful lifetime in radiation environments. Alternatively, polysaccharide adsorbents may find optimal application in low-level waste streams where total absorbed doses remain below critical degradation thresholds.

### 12.2. Techno-economic and life-cycle assessment gaps

The economic viability and environmental sustainability of polysaccharide-based adsorbents for nuclear waste treatment remain poorly characterized, with critical data gaps that prevent definitive conclusions about their competitiveness against conventional technologies. While the research question outlines theoretical cost calculations, the available literature reveals that comprehensive techno-economic analyses and life-cycle assessments for nuclear waste applications are essentially absent. Moxi Wang & X. You,^252^ provides the most relevant economic analysis of polysaccharide-based adsorbents, though focused on general water treatment rather than nuclear waste applications. Their comprehensive review of magnetic polysaccharide-based adsorbents addresses cost-effectiveness from a full life-cycle perspective, concluding that despite high raw material costs, these adsorbents can be economical compared to traditional materials due to their high performance, high recovery rate, and excellent reproducibility. The authors emphasize that regeneration and reuse performance may compensate for adverse environmental impacts, directly addressing one of the key economic concerns raised in the research question. However, their analysis reveals a critical limitation that applies to nuclear waste applications: regeneration performance of polysaccharide adsorbents typically achieves only 5–10 cycles, which is substantially lower than the 50–100 cycles achievable with synthetic ion-exchange resins. This limitation significantly impacts the economic viability calculations presented in the research question, as the cost per gram of radionuclide removed must be multiplied by the reduced regeneration capacity.

G. Díaz Bukvic *et al.*,^253^ reinforces the economic challenges by highlighting that native polysaccharides often require extensive modification to achieve suitable adsorption performance. Their review emphasizes that while polysaccharides offer abundance, biodegradability, and biocompatibility as economic advantages, the chemical modifications necessary for effective radionuclide binding substantially increase costs. This directly supports the research question's assertion that chemical modification can increase polysaccharide costs by 5–50 times, making modified versions significantly more expensive than activated carbon.

The absence of comprehensive life-cycle assessment data represents a fundamental knowledge gap. M. Nasrollahzadeh *et al.*,^254^ discusses polysaccharide-derived materials as “greener, sustainable, and eco-friendly” based on their renewable origins and biodegradability, but provides no quantitative life-cycle data such as global warming potential, water usage, or energy consumption. Their review covers starch, cellulose, pectin, alginate, chitin, and chitosan applications in water treatment, emphasizing sustainability benefits, but lacks the specific metrics needed for meaningful comparison with conventional adsorbents.

H. Al-Hazmi *et al.*,^255^ addresses environmental considerations more comprehensively, noting that successful implementation of polysaccharide-based adsorbents depends on ongoing research to optimize their application and evaluate potential environmental impacts. They specifically mention that implementing eco-friendly adsorbents on a large scale requires careful evaluation of environmental impacts, but acknowledge that such evaluations are currently lacking. Their review identifies a major concern regarding antibiotic waste discharge and notes that polysaccharide-mediated bio-adsorbents can address this issue, but again provides no quantitative life-cycle data. Nazila Biglari & E. Salehi,^256^ identifies critical challenges that directly impact industrial deployment economics. Their review highlights that inherent hydrophilicity and poor mechanical properties limit broader application of polysaccharides, necessitating nanoparticle incorporation to achieve enhanced surface area, tunable porous networks, and improved chemical and mechanical resistance. These modifications add complexity and cost to manufacturing processes, supporting the research question's assertion that high-performing polysaccharide adsorbents cost 10–50 times more than activated carbon.

The authors also discuss critical challenges including optimization of nanoparticle dispersion and environmental impacts of nanocomposite biodegradation. In nuclear waste contexts, the biodegradation concern becomes particularly acute, as noted in the research question—biodegradation may be undesirable if it releases previously adsorbed radionuclides.

Moxi Wang & X. You,^252^ provides crucial insights into regeneration economics, identifying chemical elution with suitable desorbents as the most economical, effective, and convenient approach for regenerating magnetic polysaccharide-based adsorbents. However, they acknowledge critical challenges in desorption kinetics and mechanisms that remain poorly understood. This knowledge gap directly impacts the economic calculations in the research question, as regeneration efficiency affects the total cost per gram of radionuclide removed over the adsorbent's operational lifetime. The authors note that while polysaccharide adsorbents show promise for high recovery rates, their regeneration performance over extended cycles has not been demonstrated beyond 5–10 cycles in most studies. This limitation is particularly problematic for nuclear waste applications where long-term operational stability is essential for economic viability.

The available literature supports the research question's assertion that polysaccharide adsorbents face significant economic disadvantages compared to conventional technologies. G. Díaz Bukvic *et al.*, ^253^ acknowledges that native polysaccharides often have low adsorption capacities and unsuitable physicochemical properties, requiring modification that increases costs. While they emphasize the potential for rational design of polysaccharide-based adsorbents, they provide no economic data comparing modified polysaccharides to activated carbon or synthetic resins.

## 13. Practical challenges of advanced hybrid adsorbents for radionuclide removal

Across recent advances in radionuclide adsorption, a clear convergence is observed toward engineered hybrid materials that balance selectivity, capacity, and structural stability, although important trade-offs persist between these parameters.

Table 4 Performance of advanced adsorbent materials for the removal and recovery of radionuclides from different nuclear waste streams, highlighting target ions, material types, adsorption capacities or efficiencies, mechanistic advantages, and representative alternative technologies. In Table 4 the uranium-targeted systems such as phosphorylated chitosan biochar, amidoxime-modified chitosan/bentonite, DNA-based hydrogels, and polyvinyl alcohol/chitosan/phytic acid aerogels demonstrate that high U(vi) uptake is primarily governed by strong coordination chemistry involving phosphate, amidoxime, hydroxyl, and nucleobase functionalities, often accompanied by chemisorption and, in some cases, partial reduction mechanisms. Among these, DNA-functionalized and phosphate-rich systems in Table 4 offer superior selectivity, while amidoxime-based materials provide more predictable Langmuir-type monolayer adsorption with better regeneration stability, and biochar composites achieve very high removal efficiencies but with more complex and less reversible binding pathways. In parallel, aerogel systems improve acid resistance and mechanical integrity but often lack fully comparable adsorption capacity metrics, highlighting a persistent challenge in standardization. For non-uranium radionuclides such as Cs^+^ and Sr^2+^, titanate nanotubes and alginate-based composites rely mainly on ion-exchange and electrostatic interactions, delivering fast kinetics but suffering from reduced capacity when immobilized in polymer matrices due to partial blocking of active sites. Similarly, Co^2+^ and Eu^3+^ removal using alginate–bentonite hybrids shows that while composite formation enhances handling and recyclability, it can dilute adsorption efficiency compared to pristine nanomaterials. Overall, despite impressive laboratory-scale performances, most systems still face common limitations including competition from coexisting ions, reduced accessibility of active sites in real wastewater, and incomplete evaluation under true nuclear effluent conditions. Consequently, future progress should focus on integrating high-affinity functional groups into mechanically robust, hierarchically porous architectures while ensuring scalability, regeneration efficiency, and standardized performance assessment under realistic environmental conditions.

**Table 4:** Comparative performance of advanced adsorbent materials for the removal and recovery of radionuclides from different nuclear waste streams, highlighting target ions, material types, adsorption capacities or efficiencies, mechanistic advantages, and representative alternative technologies

Nuclear waste stream	Target radionuclide(s)	Recommended material	Capacity (mg g^−1^)	Why this choice	Alternatives	Ref.
Uranium-containing wastewater (UCW)	U(vi)	Polyvinyl alcohol/chitosan/phytic acid aerogel (PCPA)	Not specified (96.3% removal efficiency)	High acid stability, strong selectivity, fast adsorption kinetics, excellent ion-interference resistance, and reusable (>80% after 5 cycles). PVA enhances mechanical stability and –OH functionality improves selectivity for U(vi)	Ion-exchange resins, ferrocyanides, zeolites, clay minerals	257
Radioactive wastewater (mixed radionuclides)	Cs^+^, Sr^2+^	Titanate nanotubes embedded in alginate (TNTs/T-G beads)	Not specified (Cs ∼90%, Sr ∼97% removal)	Very fast kinetics (15–30 min), strong ion-exchange capability, high affinity for Sr^2+^, good reusability (>85–90%), and easy solid–liquid separation *via* alginate matrix	Zeolites, ammonium molybdophosphate, ferrocyanides, ion-exchange resins	88
Radioactive wastewater	^60^Co(ii), ^152^+^154^Eu(iii)	Nanobentonite@alginate@oleylamine (Nbent@Alg@OA)	Eu: 65.62 mg g; Co: 47.35 mg g^−1^	High adsorption capacity, chemisorption-driven uptake, multilayer adsorption behavior, good thermal stability, and efficient regeneration over multiple cycles	Clay minerals, zeolites, inorganic ion exchangers	258
Uranium-contaminated water	U(vi)	Phosphorylated chitosan-functionalized biochar (PCTS)	Not explicitly stated (98.85% removal)	Dual mechanism: strong complexation *via* phosphate groups and partial reduction of U(vi) to U(iv); multilayer adsorption improves efficiency	Activated carbon, unmodified biochar, metal oxides	85
Uranium in seawater	U(vi)	Amidoxime-modified chitosan/bentonite composite	49.09 mg g^−1^	High affinity amidoxime groups, chemisorption-based Langmuir monolayer adsorption, strong performance in seawater conditions, good regeneration (6 cycles)	Ferrocyanides, functionalized resins, polymer adsorbents	83
Uranium in seawater	UO_2_^2+^	SA-DNA hydrogel microspheres	189.5 mg g^−1^	Ultra-high selectivity (43.6× *vs.* V ions), highly porous structure improves mass transfer, DNA provides specific coordination sites for uranyl binding	Alginate beads, ion-imprinted polymers, chitosan hydrogels	259

## 14. Future respective and conclusion

The future of polysaccharide-based adsorbents for radioactive waste management lies in addressing current limitations while expanding their applicability in real-world scenarios. One key direction is the development of multifunctional composites that integrate polysaccharides with advanced materials, such as graphene oxide, metal–organic frameworks (MOFs), or nanoparticles, to enhance selectivity, mechanical stability, and radiation resistance. Additionally, process intensification through hybrid systems such as membrane filtration, electrosorption, or photocatalytic degradation could improve efficiency in large-scale wastewater treatment. Another critical area is the optimization of regeneration and reusability, ensuring that adsorbents maintain high performance across multiple cycles while minimizing secondary waste. Computational modeling and artificial intelligence could further aid in the design of tailored adsorbents with optimized binding affinities for specific radionuclides. Finally, life-cycle assessments (LCAs) and techno-economic analyses are needed to evaluate the environmental footprint and cost-effectiveness of these materials compared to conventional methods, ensuring their feasibility for industrial adoption.

Polysaccharide-based adsorbents represent a sustainable and efficient solution for radioactive waste remediation, offering advantages such as biodegradability, low cost, and tunable functionalization. Research has demonstrated their exceptional performance in removing key radionuclides (*e.g.*, U(vi), Cs^+^, Sr^2+^, and TcO_4_^−^) *via* mechanisms such as ion exchange, chelation, and electrostatic interactions. Chemical modifications, including cross-linking, grafting, and hybridization, have significantly enhanced their adsorption capacity and selectivity. However, challenges remain regarding stability under extreme conditions (*e.g.*, high radiation levels, acidic/alkaline environments) and competition from coexisting ions in complex waste streams. Moving forward, interdisciplinary efforts combining materials science, environmental engineering, and computational design will be crucial in advancing these biosorbents toward practical implementation. By addressing scalability, reusability, and economic viability, polysaccharide-based materials can play a pivotal role in achieving sustainable nuclear waste management and environmental protection.

## Conflicts of interest

The authors declare that they have no conflict of interest.

## Abbreviations

AC: Activated carbon
AC@Alg: Activated carbon derived from waste masks grafted with alginate
AC@CS: Activated carbon grafted with chitosan
Alg: Alginate
Am: Americium
AMCS: Amine-modified chitosan
AMP: Ammonium molybdophosphate
AO-Alg: Amidoxime-functionalized alginate beads
AO-ChB: Amidoxime-functionalized chitosan beads
AO-CS/GO: Amidoxime-modified chitosan/graphene oxide composites
AO-CNF: Amidoxime-functionalized cellulose nanofibers
AOCE: Amidoxime-functionalized cellulose derivative
BET: Brunauer–Emmett–Teller (surface area analysis)
BMIM-BF_4_: 1-Butyl-3-methylimidazolium tetrafluoroborate (ionic liquid)
Bn-CTS: Bentonite-chitosan beads
Ca^2+^: Calcium ion
CBA: Chitosan-bentonite-silver hydrogel
CCD: Charge-coupled device
CD: Carbon dots
Ce-Alg: Cerium-alginate beads
CG-PAM: κ-Carrageenan/polyacrylamide semi-IPN hydrogel
Ch/PVA-HA: Chitosan/polyvinyl alcohol-humic acid composite
Ch-VL: Chitosan-vermiculite-lignin composite
CL/MB: Cellulose/ball-milled bone char composite
CLM: l-cysteine bridged chitosan/Mg–Al hydrotalcite foam
CMC: Carboxymethyl cellulose
CMS: Carboxymethyl starch
CNC: Cellulose nanocrystal
CNF: Cellulose nanofiber
COF: Covalent organic framework
CPL: Clinoptilolite
C-PGCB: Phosphorylated chitosan/graphene oxide gel bead
CQDs: Carbon quantum dots
CS: Chitosan
CSA: Chitosan aerogel
CSP: Phosphorylated chitosan
CST: Crystalline silicotitanate
Cs^+^: Cesium ion
CuHCF: Copper hexacyanoferrate
Cu-PBA: Potassium copper hexacyanoferrate(II) (Prussian blue analogue)
CV: Cyclic voltammetry
DFT: Density functional theory
DNOA-GO-CS: Tertiary amine-grafted graphene oxide-chitosan aerogel
DOTA: 1,4,7,10-Tetraazacyclododecane-1,4,7,10-tetraacetic acid
DTPA: Diethylenetriaminepentaacetic acid
D–R: Dubinin–Radushkevich (isotherm model)
EDTA: Ethylenediaminetetraacetic acid
EDS/EDX: Energy-dispersive X-ray spectroscopy
EXAFS: Extended X-ray absorption fine structure
Fe_3_O_4_: Magnetite
FTIR: Fourier-transform infrared spectroscopy
GMA: Glycidyl methacrylate
GO: Graphene oxide
GQDs: Graphene quantum dots
HA: Humic acid
HAp: Hydroxyapatite
HDEHP: bis(2-ethylhexyl) phosphoric acid
HNT: Halloysite nanotube
IA-g-St/MnO_2_: Itaconic-grafted starch/MnO_2_ nanocomposite
IDA: Iminodiacetic acid
II-CLSPC: Ion-imprinted starch phosphate porous carbonaceous material
IIP: Ion-imprinted polymer
IIP-Alg/LHT: Ion-imprinted alginate/layered hydrotalcite foam
IIP-CS/LHT: Ion-imprinted chitosan/layered hydrotalcite foam
KCuHCF: Potassium copper hexacyanoferrate
Kd: Distribution coefficient
KGM: Konjac glucomannan
M-ACFBs: Magnetic alginate-chitosan foam beads
MAlg/Z: Magnetic alginate/zeolite nanocomposite
MCC: Microcrystalline cellulose
MCS/Z: Magnetic chitosan/zeolite nanocomposite
MOF: Metal–organic framework
MPCA: Phosphate-functionalized magnetic alginate
MXene@i.Carr@MaMb: MXene functionalized with iota-carrageenan, maleic anhydride, and *N*,*N*′-methylene bis-acrylamide
NMR: Nuclear magnetic resonance
O-CMC: O-carboxymethyl chitosan
PAA: Poly(acrylic acid)
PAFP: Poly(anthranilic acid-formaldehyde-phthalic acid)
PAM: Polyacrylamide
PAN: Polyacrylonitrile
PBA: Prussian blue analogue
PBTCA: 2-Phosphonobutane-1,2,4-tricarboxylic Acid
PCAR: Porous phosphorylated chitosan-amidoxime resin composite
PCT: Polyethyleneimine(PEI)-grafted chitosan/nano-TiO_2_ composite foams
PEI: Polyethylenimine
PEPA: Polyethylene polyamine
PMAA: Polymethacrylic acid
PSMGPB: Pectin-stabilized magnetic graphene oxide Prussian blue nanocomposite
Pu: Plutonium
PVA: Polyvinyl alcohol
PVPA: Poly(vinylphosphonic acid)
*q*_max_
: Maximum adsorption capacity
rGO: Reduced graphene oxide
SANS: Small-angle neutron scattering
SAXS: Small-angle X-ray scattering
SEM: Scanning electron microscopy
sHNT: Silanated halloysite nanotubes
SP: Starch phosphate
Sr^2+^: Strontium ion
TEPA: Tetraethylenepentamine
TGA: Thermogravimetric analysis
Th: Thorium
TMC: Thiourea-modified chitosan membranes
TOCNF: TEMPO-oxidized cellulose nanofibrils
TU-Starch/GO: Thiourea-modified starch-graphene oxide composite
U-AOCE: Uranyl ion-imprinted amidoxime-functionalized cellulose Derivative
U-APCS: Uranyl ion-imprinted amino-phenolic functionalized chitosan
VSM: Vibrating sample magnetometry
XPS: X-ray photoelectron spectroscopy
XRD: X-ray diffraction

## Data Availability

No primary research results, software or code have been included and no new data were generated or analysed as part of this review.
